# Individual-, family- and school-based interventions to prevent multiple risk behaviours relating to alcohol, tobacco and drug use in young people aged 8-25 years: a systematic review and meta-analysis

**DOI:** 10.1186/s12889-022-13072-5

**Published:** 2022-06-03

**Authors:** Laura Tinner, Jennifer C Palmer, E. Caitlin Lloyd, Deborah M Caldwell, Georgie J MacArthur, Kaiseree Dias, Rebecca Langford, James Redmore, Linda Wittkop, Sarah Holmes Watkins, Matthew Hickman, Rona Campbell

**Affiliations:** 1 Bristol Medical School, Population Health Sciences, University of Bristol, 39 Whatley Road, Bristol, BS8 2PS UK; 2 MRC Integrative Epidemiology Unit at the University of Bristol, Bristol, UK; 3 Department of Psychiatry, Columbia University Irving Medical Center, New York, New York USA; 4 New York State Psychiatric Institute, New York, New York USA; 5 York Teaching Hospitals NHS Foundation Trust, Wiggington Road, York, UK

## Abstract

**Background:**

Engagement in multiple substance use risk behaviours such as tobacco smoking, alcohol and drug use during adolescence can result in adverse health and social outcomes. The impact of interventions that address multiple substance use risk behaviours, and the differential impact of universal versus targeted approaches, is unclear given findings from systematic reviews have been mixed. Our objective was to assess effects of interventions targeting multiple substance use behaviours in adolescents.

**Methods:**

Eight databases were searched to October 2019. Individual and cluster randomised controlled trials were included if they addressed two or more substance use behaviours in individuals aged 8-25 years. Data were pooled in random-effects meta-analyses, reported by intervention and setting. Quality of evidence was assessed using GRADE. Heterogeneity was assessed using between-study variance, τ2 and *Ι*^2^, and the *p*-value of between-study heterogeneity statistic Q. Sensitivity analyses were undertaken using the highest and lowest intra-cluster correlation coefficient (ICC).

**Results:**

Of 66 included studies, most were universal (*n*=52) and school-based (*n*=41). We found moderate quality evidence that universal school-based interventions are likely to have little or no short-term benefit (up to 12 months) in relation to alcohol use (OR 0.94, 95% CI: 0.84, 1.04), tobacco use (OR 0.98, 95% CI: 0.83, 1.15), cannabis use (OR 1.06, 95% CI: 0.86, 1.31) and other illicit drug use (OR 1.09, 95% CI: 0.85, 1.39). For targeted school-level interventions, there was low quality evidence of no or a small short-term benefit: alcohol use (OR 0.90, 95% CI: 0.74-1.09), tobacco use (OR 0.86, 95% CI: 0.66, 1.11), cannabis use (OR 0.84, 95% CI: 0.66-1.07) and other illicit drug use (OR 0.79, 95% CI 0.62-1.02). There were too few family-level (*n*=4), individual-level (*n*=2) and combination level (*n*=5) studies to draw confident conclusions. Sensitivity analyses of ICC did not change results.

**Conclusions:**

There is low to moderate quality evidence that universal and targeted school-level interventions have no or a small beneficial effect for preventing substance use multiple risk behaviours in adolescents. Higher quality trials and study reporting would allow better evidence syntheses, which is needed given small benefit of universal interventions can have high public health benefit.

**Trial registration:**

Cochrane Database of Systematic Reviews 2014, Issue 11. Art. No.: CD011374. DOI: 10.1002/14651858.CD011374.

**Supplementary Information:**

The online version contains supplementary material available at 10.1186/s12889-022-13072-5.

## Introduction

Risk behaviours such as tobacco smoking, antisocial behaviour, hazardous alcohol consumption, physical inactivity and unprotected sexual intercourse are commonly initiated in adolescence [1]. These risk behaviours have also been shown to co-occur in adolescence, with engagement in one behaviour increasing the likelihood of engaging in others [2]. Multiple risk behaviour (MRB) refers to the engagement in two or more behaviours directly or indirectly associated with health, well-being and healthy development of personality [3]. There is evidence that MRB is associated with negative outcomes over and above the effects of the individual risk behaviours, including increased risk of premature mortality, morbidity, poor educational attainment, obesity and mental health problems later in life [4–6]. Given that adolescents make up a quarter of the world’s population, youth represents a critical time in the life course to address MRB for the improvement of health and wellbeing [7].

The co-occurrence of risk behaviours in adolescence is of particular importance to substance use behaviours. For instance, tobacco smokers are more likely to consume alcohol and vice versa, with cannabis users being more likely to engage in other drugs [8–10]. A UK study found that adolescents who engaged in hazardous alcohol use were six times more likely to engage in tobacco and drug use [11]. Prevalence estimates of concurrent alcohol, tobacco and drug use among young people range from 4% to 17% [10, 12–16]. Given that engagement in these substance use behaviours may continue from adolescence into early adulthood [17–19] MRB related to substance use is an important area of investigation due to the high public health costs of these behaviours and burden to society [20].

Recognising the co-occurrence of substance use risk behaviours and shared antecedents [21–23] has led to recommendations for interventions that address multiple, rather than single, risk behaviours in an efficient approach that can provide a cost-effective means of prevention [24, 25]. However, there are few examples of reviews that examine the effectiveness of interventions targeting substance related MRB. Typically, reviews focus on interventions that target behaviours such as alcohol use [26, 27], tobacco use [28] and illicit drug use [29] in isolation. One review assessed interventions targeting MRB and found that a large proportion of identified interventions targeted substance use behaviours [30]. The authors found prevention programmes to be effective, with small effect sizes but comparative to single risk behaviour interventions [30]. A more recent review assessed combined student- and parent-based programs and found that nine programmes had promise in preventing alcohol and other drug use among adolescents [31]. There has also been an overview of systematic reviews showing small but consistent positive effects of school-based prevention programmes targeting alcohol and drug misuse in 12-18 year olds [32]. Finally, other systematic reviews have assessed MRB including substance use behaviours, but either set out to undertake a narrative synthesis or due to heterogeneity of studies were unable to conduct quantitative meta-analyses [33, 34]. Our review differs from these studies as we include a wider variety of intervention settings, include any combination of substance use behaviours and assess interventions delivered at the individual-, family- and school-level.

### Objectives

The primary objective of this review was to assess the effects of interventions at the individual-, family- and school-level that target multiple substance use risk behaviours (two or more from alcohol, tobacco, cannabis and other substance use) for the primary or secondary prevention of MRB and related harms in children and young people aged 8-25 years. It is the partner review to MacArthur et al. [24], which examined interventions that targeted two or more risk behaviours with at least one being a non-substance use risk behaviour (such as risky sexual behaviour or anti-social behaviour).

## Methods

### Search strategy and study selection

A peer-reviewed, published protocol is available for this review [35]. This review was originally part of a Cochrane registered review addressing a wide range of risk behaviours. However, due to the identification of many studies that addressed a combination of substance use risk behaviours without addressing other risk behaviours, we decided to narrow the scope of this review. Thus, this review focuses on interventions targeting a combination of at least two or more substance use risk behaviours only. Searches were conducted in three stages, reflecting initial and update searches. Initial searches were conducted prior to the reviews being separated [24, 35]. Seventeen electronic databases were searched, from inception to May 2012, including websites and databases for ongoing or unpublished studies, without language restriction. The reference lists of relevant articles were hand searched and experts in the field contacted to identify ongoing research. Following MacArthur et al [24], database selection was streamlined to eight databases, and search terms refined to improve specificity of update searches in 2018 and 2019. Further details on the approach and search strategies for Medline are provided in Additional file 1.

The title and abstracts of the first 500 records from the May 2012 searches were independently screened by two authors, and a further 10% were randomly selected and double-screened for quality assurance. Kappa statistics showed a high level of agreement (>0.8). The remainder of the title and abstract screening was conducted by a single reviewer. Full text screening was conducted independently by two reviewers. For the 2018 and 2019 update searches, after the reviews had been separated [35, 36], two independent reviewers screened all records for inclusion, at both title and full-text screening stages. Disagreements were resolved by discussion, or by a third reviewer if necessary.

### Eligibility criteria

Studies were eligible for inclusion if they were randomised controlled trials (RCTs), including clustered RCTs, aimed at addressing at least two substance use behaviours of interest, including: tobacco smoking, alcohol consumption, cannabis use and other substance use e.g. illicit drugs, legal highs, solvents, aerosols, or inhalants. Eligible participants were children and young people aged 8-25 years of age. Interventions targeting parents, carers, peers, teachers and others were eligible if the intended impact of the intervention was to prevent or reduce engagement in MRBs among children and young people aged up to 18 years (at study baseline, but up to 25 years at follow-up). Studies were included if they had a minimum follow up period of six months from the start of the intervention. Interventions could be provided universally (without regard of the young person’s level of risk) or targeted towards young people or families thought to be high risk. Interventions could be delivered in a wide variety of individual-, family-, or school-settings, and across the prenatal, antenatal, nursery, preschool, primary and secondary school ages. Delivery could include a variety of methods and individuals such as trained staff members, nurses, teachers, parents, peers and the police or via electronic equipment. Eligible comparator interventions included usual practice (as defined by the study author), no intervention or placebo.

### Exclusion criteria

Interventions that addressed additional risk behaviours not classified as substance use were excluded as they were assessed in the companion Cochrane review [24]. Interventions delivered at a community- or population-level, such as media campaigns or policy, regulatory or legislative were excluded. Interventions with a follow-up period of less than six months (including the intervention period) were excluded. Interventions aimed at individuals with clinically diagnosed disorders or interventions where the majority of the adolescent participants were 18 years at baseline assessment were also excluded. Details of excluded and ongoing studies can be found in Additional file 2.

### Data extraction

Data were extracted directly into a piloted form in Microsoft Excel. Details on study characteristics and primary and secondary outcome data extracted are in Table 1. Data were extracted by one reviewer and checked by a second with disputes resolved between them or by involving a third reviewer. Study authors were contacted for further information if there were missing or unclear data in the full text publications. No automation tools were used. The full list of extracted data is reported in the protocol [35].

**Table 1:** Data extracted on characteristics, primary and secondary outcomes

**Study characteristics**	**Primary outcome data**	**Secondary outcome data**
Authors, design, location, setting, duration, substance use behaviours addressed, intervention content and activities, theoretical underpinning, types of participants, duration of follow-up, statistical details (including whether clustering was accounted for)	All data relating to use of alcohol (primary outcomes: alcohol use or frequency, and binge drinking), tobacco (primary outcomes: tobacco use or frequency, and heavy smoking) and other drug use (primary outcomes: cannabis use or frequency, and other illicit drug use^1^). Outcome data were extracted as reported by the study authors for all follow-up timepoints. This included raw dichotomous and continuous data at baseline and follow-up (e.g. number of events and participants, or mean and standard deviations), effect sizes and model estimates (with uncertainty)	Secondary outcomes included: education and employment (e.g. educational qualifications, employment, not being in education, employment or training (NEET), receipt of government benefits); crime; long term addictive behaviours; health outcomes (e.g. teenage pregnancy or parenthood, mental health outcomes, morbidity, premature mortality); dependence or harmful substance use; adverse events as a result of the intervention; and cost effectiveness of the intervention

Legend: The table lists the data extracted related to study characteristics, primary and secondary outcome data. ^1^“Other illicit drug use” could consist of prescription drug, poly illicit drug, inhalant, cocaine, meth/amphetamine, or ecstasy use. Some of the poly illicit drug use outcomes included cannabis

### Quality assessment

Risk of bias was assessed by two review authors using the Cochrane Risk of Bias 1.0 tool [37], with disagreements resolved through discussion and inclusion of a third reviewer if needed. Study quality was assessed by one reviewer using Grades of Recommendation, Assessment, Development and Evaluation (GRADE) criteria [38].

### Data handling and preparation

Following data extraction, some studies could not be included in the synthesis because they either did not report substance use outcome data or reported a combined substance use outcome (such as a combined alcohol, tobacco and drug use score) which was not of interest for this review. A list of these studies can be found in Additional file 3 [39–47].

We anticipated outcome data would be inconsistently reported and summarised across studies [35]. The main data transformations and assumptions made are described here and in more detail in Additional file 4. To maximise the number of studies available for meta-analysis, we converted all outcome data to a log odds ratio scale [40, 41]. Where cluster RCTs had ignored clustering in their analyses, or where it was not clear, we inflated the SE to account for intra-cluster correlations and to avoid a unit-of-analysis error [37] (See Additional file 3). Where the number of participants in each study arm was unclear, but where total number of participants was provided and attrition was comparable between each arm, we assumed equal group sizes. In multi-arm trials, intervention groups were combined where they were deemed similar enough. The calculations and formulae used for combining were dependent on data type and are described in Additional file 4. Where studies did not provide sufficient information for meta-analysis, or data transformations were not possible, studies were omitted from analysis. A list of studies, with reason for omission, is provided in Additional file 3.

### Substance-use outcomes

Although the interventions in this review address multiple substance use behaviours, the majority of studies report substance use outcomes separately (not as a composite MRB score). Therefore, as specified in the review protocol to allow for meta-analysis and to align with the partner review [24] we have reported the behaviour outcomes separately, and our intentions outlined in the protocol [35]. Where a study reported multiple measures per outcome, a pre-specified hierarchy was applied: [1] the ‘most harmful’ measure from a frequency and public health perspective (more recent and/or more regular use) and [2] the measure which contained more information were preferred for meta-analysis. For example, a recent, regular measure of drinking frequency (e.g. average number of drinks per week) was preferred to a longer-term binary measure of drinking (e.g. drunk alcohol in the past month). Where authors reported multiple ‘heavy use’ outcomes such as ‘binge drinking’, ‘heavy drinking’, ‘being drunk’ and ‘daily cigarette use’, the same hierarchy was applied.

After applying the above criteria, the outcome measures included are detailed in Table 2. Further detail on outcomes contributing to the meta-analyses is reported in Additional file 5.

**Table 2:** Included substance use outcome measures

**Substance-use outcome**	**Included measures**
Alcohol use	Alcohol use (yes/no) in the past week, month or year; number of occasions of alcohol use in the past month; mean alcohol units per day/week or in the last month; ever used alcohol
Tobacco use	Tobacco use (yes/no) in the past week, month or year; number of occasions of tobacco use in the past month; mean cigarettes per day/week or in the last month; ever used tobacco
Cannabis use	Cannabis use (yes/no) in the past week, month or year; number of occasions of cannabis use in the past month; mean joints per day/week or in the last month; ever used cannabis
Heavy alcohol use	Binge drinking or drunkenness (yes/no) in the past month; 3 or more episodes of drunkenness (yes/no) in the past month; heavy drinking in the past month (as defined by study authors)
Other illicit drug use	Hard drug/cocaine/inhalant-use in the past month; number of occasions of prescription drug/poly-drug/hard drug/other drug/inhalant-use in the past month; ever used meth/amphetamine/ecstasy/cocaine/illicit drugs (some included cannabis) or sniffed glue

Legend: The table outlines the included measures for each type of substance use behaviour

### Data synthesis and subgroup analysis

We used the metafor package in R 4.0.2 [48], to conduct random effects meta-analyses allowing for between study variation in intervention effects (i.e. different studies could estimate different, yet related, intervention effects) [49]. Separate analyses were conducted by outcome and by short-term (closest to 12 months) and longer term (longest available) follow-up periods. Results are summarised as odds ratios (ORs) with 95% confidence intervals (CIs). Heterogeneity was evaluated using the between-study variance, τ2, and Ι^2^ measure, as well as the p-value of between-study heterogeneity statistic Q (based on the random effects model). We planned to investigate heterogeneity using subgroup analyses, and the full list of planned analyses is available in the protocol and Additional file 6. However, with the exception of “intervention type” (study setting and intervention focus) there were insufficient numbers of studies for meaningful subgroup analysis. Following MacArthur [24], and for ease of presentation, our results are presented on a single forest plot grouped by intervention type and setting.

### Small-study effects and sensitivity analyses

Where there was a sufficient number of studies (minimum of 10), we used funnel plots to examine the study effect size against the sample size to look for publication bias or small-study effects [50]. For the study outcomes that required us to estimate an ICC, we carried out sensitivity analyses by using the highest and lowest reported ICC to calculate design effects and inflated SEs for each outcome. We ran meta-analyses for each group using these robust SEs to calculate best- and worst-case scenarios. Planned sensitivity analyses focusing on studies at low risk of bias were not conducted due to insufficient numbers (Additional file 6).

## Results

### Search results

**Fig. 1:**
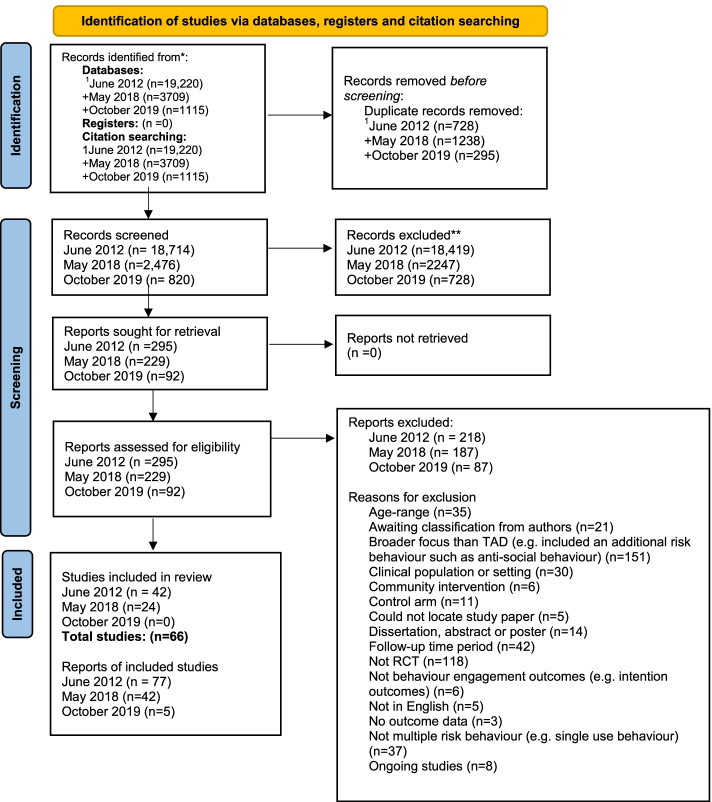
Flow diagram detailing the systematic review screening process. Shows the systematic review process included the number of studies that were retrieved at the different search points, the number of studies excluded at each stage and the final sample of intervention studies

From a total of 24,263 titles, we obtained 141 full text articles and included 66 studies in the systematic review (Fig. 1).

### Study characteristics

A summary of study characteristics is provided in Additional file 7. Sixty-three studies were conducted in high-income countries (Australia *n*=4; Germany *n*=1; Netherlands *n*=1; Multi-country *n*=2; Spain *n*=3; Sweden *n*=1; Taiwan *n*=2; UK *n*=4; USA *n*=45) and three studies were conducted in middle-income countries (Thailand, Mexico, Brazil). Studies were either RCTs (*n*=13) or cluster RCTs (*n*=53). Most studies adopted a universal prevention approach (*n*=53), of which 41 were implemented in school settings, five in family settings, five in a combination of settings and two focussed on individuals. Thirteen studies were targeted at high-risk groups, for example, as determined by behaviour scores (indicated) or by ethnic or socioeconomic factors (selective). These targeted interventions were at the school level (*n*=8); at the family-level (n=1), at the individual level (*n*=1) and at a combination of the levels (*n*=3).

Studies that reported using a theoretical framework (*n*=45) varied in terms of type of theory, number of theories and combinations. The most commonly used theories were Social Influence Model (*n*=14), Social Learning Theory (*n*=11) and Health Belief Model (*n*=6). Most studies used a sociopsychological, cognitive or behavioural theory or model as the framework, with many taking inspiration from several models instead of strictly following one. There was also a range of activities used within the interventions. All studies used a curriculum to guide intervention activities (*n*=45), with reported details of the curriculum varying. Studies that reported what the curriculum involved mentioned elements such as interactive activities, workbooks, quizzes, teacher-led classes and demonstrations. Other key components (reported separately to the curriculum, but could have been embedded within it) included role play (*n*=33), homework (*n*=17), group discussion (*n*=27), parental involvement (*n*=13) and didactic teaching (*n*=16). Theoretical frameworks and components were spread across the different study types and levels.

Intervention duration varied across studies. The most common intervention duration was <3 months (*n*=28), with the remainder lasting 3-6 months (*n*=10), 6-12 months (*n*=4), 12-24 months (*n*=10) and more than 24 months (*n*=6). Few studies explicitly reported intervention length and for eight (12%) it could not be determined at all. Studies targeted either two (*n*=16), three (*n*=28) or four (*n*=22) substance use behaviours concurrently. Thirty five studies reported the number of randomised individuals, either in total or by intervention/control arm. Total sample size ranged from 70 to 19,529 individuals. Most studies stated the control group received no intervention or usual practice (*n*=49). Eleven studies stated the control group received an alternative intervention. Six studies did not describe what the control group received. Most studies were two-armed studies (intervention/control) (*n*=41), with the remainder either not stating the number of arms or being three and four armed studies including comparing multiple interventions.

Participant age at baseline was inconsistently reported and a variety of summaries were used. For example, mean age (with and without standard deviation), age range, proportions, approximate age ‘average’, school grade or school grade range. Participant age at baseline could not be determined in 10 studies. Of those studies reporting an age summary (*n*=56), we cautiously estimated that most addressed early adolescents between 11 and 14 years (*n*=36) with the remainder (*n*=20) including older adolescents (up to 18 years). No studies indicated participants were under 10 years at baseline. Most studies reported gender of participants at baseline (*n*=60). Four studies only included female participants, the remainder were mixed with the majority an approximate equal split of male and female, with two studies having over 60% female participants [39, 51] and two studies having 31-33% female participants [52, 53]. Thirty studies reported a baseline socioeconomic measure, with a wide variety of summaries including free school meal eligibility, parental occupational status and household income at either an individual or school level. Forty studies reported ethnicity at baseline. Two studies were targeted at one ethnic minority group [54, 55]. For the remainder of studies there were varying levels of detail around ethnicity (e.g. only reporting the majority ethnic group) and whether reported at individual or school level.

### Quality of evidence

Here we report the risk of bias assessments for the domains of randomised sequence generation and allocation concealment only, with results for all domains reported in the final column of Additional file 7. Six studies were at low risk of bias for both domains, with a further seven at low risk for just randomised sequence generation. Forty-five studies were at unclear risk of bias for both randomised sequence generation and allocation concealment. The remaining studies were at high risk of bias for at least one domain. Using GRADE criteria, the quality of the evidence was rated as being of moderate or low certainty for most outcomes. This quality of evidence was primarily due to risk of performance bias and unexplained heterogeneity between studies.

### Meta-analyses of substance-use outcomes

Short- and long-term effects of interventions on each substance-use outcome were meta-analysed separately. Heterogeneity across all subgroups was substantial due to differences in population targeted and intervention type (*I*^*2*^ ranged from 0% to 71%, for short-term effects and 97% to 47%, for long-term effects). Therefore, we analysed pre-planned subgroups according to population targeted (universal and targeted) and intervention type (individual, family, school and combination). For full transparency we also report results from fixed effect analyses and the overall pooled summary effects for both random and fixed effects models in Additional file 8.

### Short-term intervention effects (one year post-intervention)

#### Alcohol use and binge drinking

**Fig. 2:**
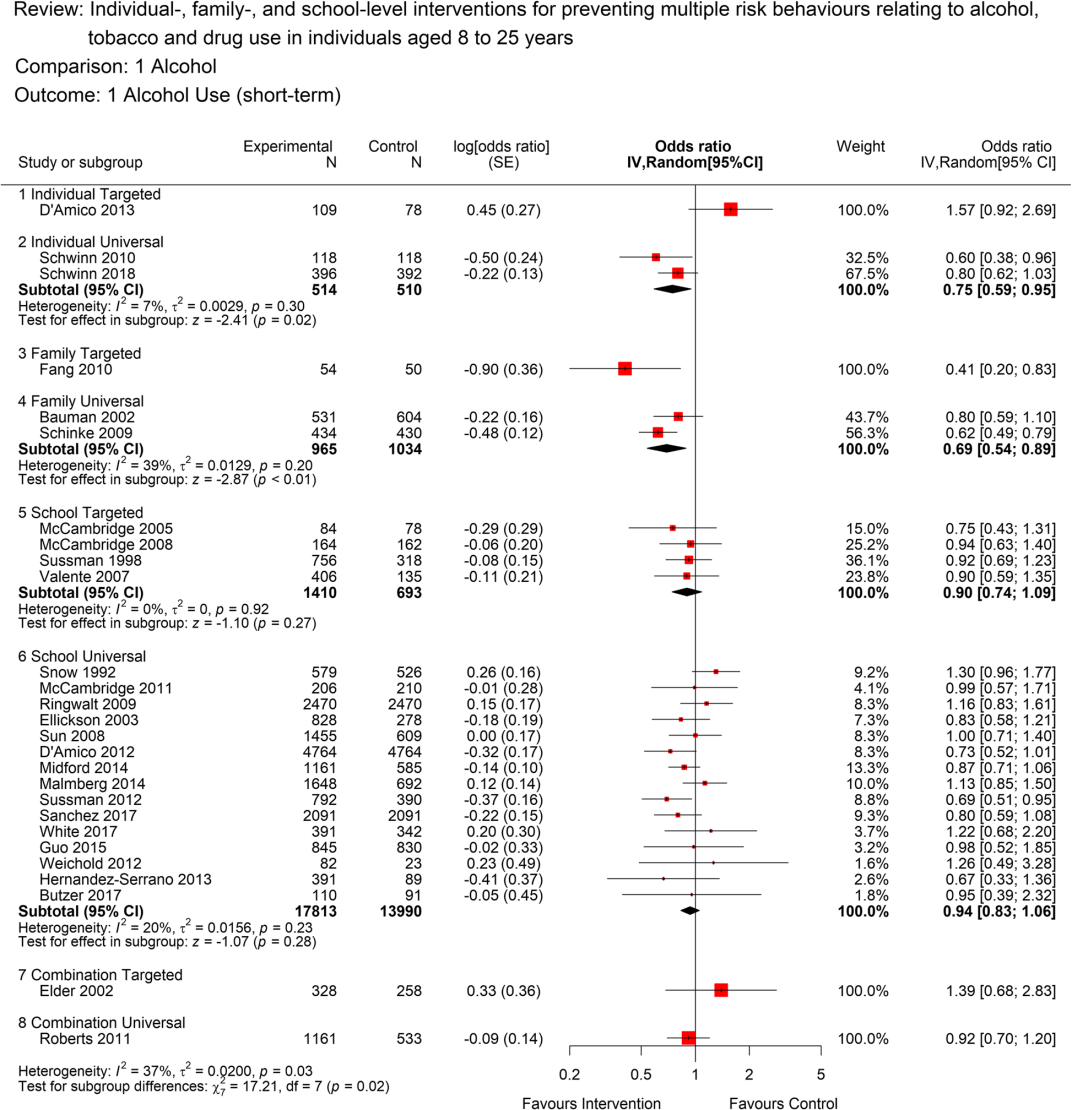
Forest plot for short term alcohol use (<12 months) by intervention level. The plot shows the data meta-analysed in intervention-level subgroups (school, family, individual, combination and universal or targeted) for the short-term (as close to 12 months post-intervention end as possible) alcohol use outcome. Red boxes represent point estimate (odds ratio) and horizontal lines the 95% confidence intervals. Black diamonds are pooled estimates for each subgroup. Estimates above 1 (on the right-hand side of the y-axis labelled ‘favours control’) suggests a lower alcohol use in the control group compared to the intervention group, while estimates below 1 (on the left-hand side of the y-axis labelled ‘favours intervention’ suggests lower alcohol use in the intervention group compared to the control group. The overall pooled estimate was not calculated as the subgroups differ by design and a decision was made a priori to report subgroups separately

Twenty-seven studies contributed to the analyses for alcohol use (Fig. 2). There was moderate quality evidence that universal, school-based interventions have no or a small beneficial effect in relation to short-term alcohol use (OR 0.94, [95% CI: 0.84 to 1.04; 15 studies; 31,803 participants; *I*^*2*^=20%). There was moderate-quality evidence, that universal, family-level interventions (OR 0.69, [95% CI: 0.54 to 0.89]; 2 studies; 1999 participants; *I*^*2*^=39%) and universal, individual-level interventions (OR 0.75, [95% CI: 0.59 to 0.95]; 2 studies; 1024 participants; *I*^*2*^=7%) were effective for preventing short-term alcohol use.

For targeted school-based interventions, there was low quality evidence of no or a small beneficial effect of the intervention in the short term (OR 0.90, [95% CI: 0.74 to 1.09]; 4 studies; 2103 participants; *I*^*2*^=0%). Other intervention and setting groups contained only one study each but are reported in Fig. 2 to enable a visual comparison.

**Fig. 3:**
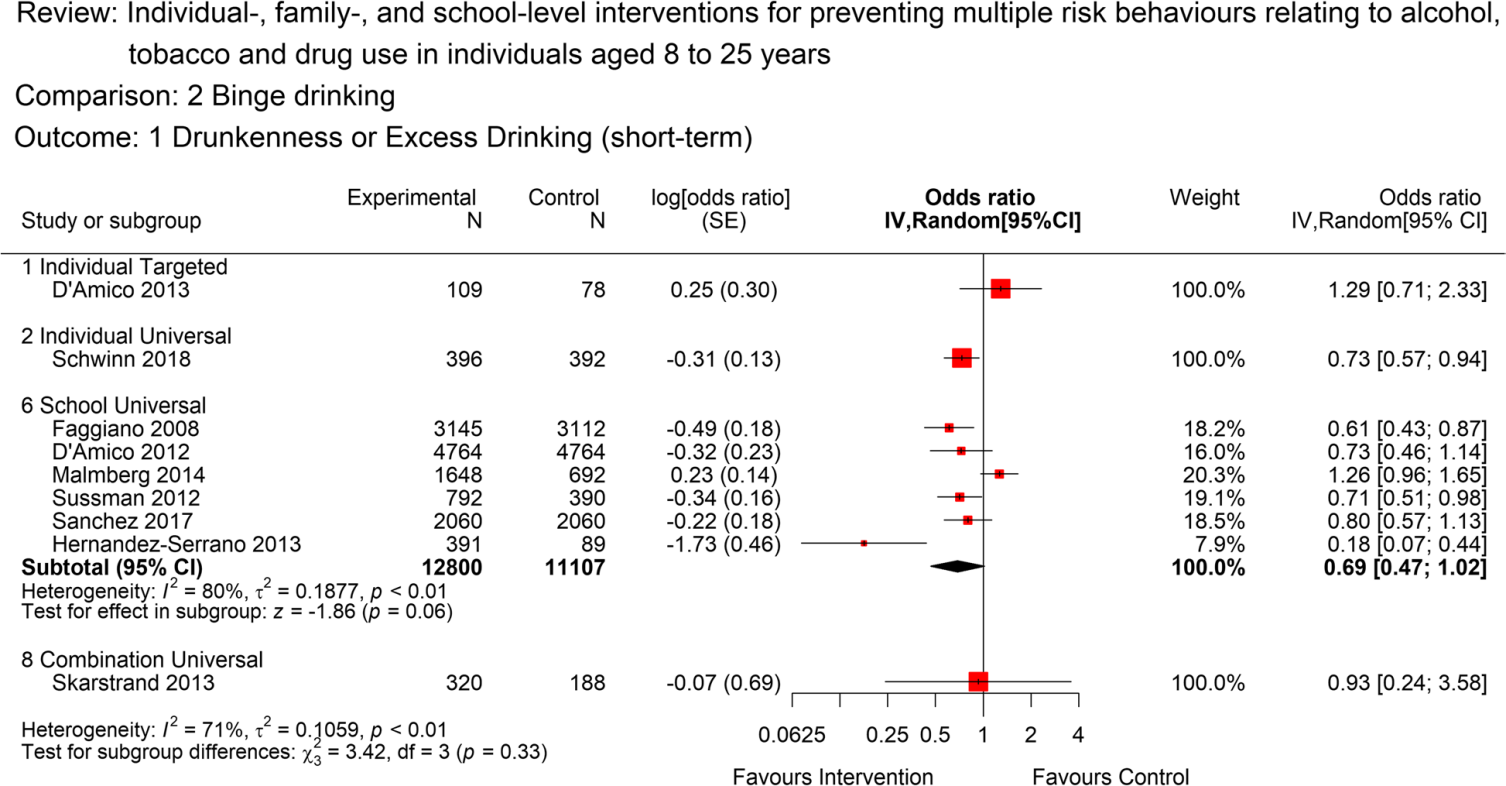
Forest plot for short term heavy alcohol use (<12 months) by intervention level.The plot shows the data meta-analysed in intervention-level subgroups (school, family, individual, combination and universal or targeted) for the short-term (as close to 12 months post-intervention end as possible) heavy alcohol use outcome. Red boxes represent point estimate (odds ratio) and horizontal lines the 95% confidence intervals. Black diamonds are pooled estimates for each subgroup. Estimates above 1 (on the right-hand side of the y-axis labelled ‘favours control’) suggests a lower heavy alcohol use in the control group compared to the intervention group, while estimates below 1 (on the left-hand side of the y-axis labelled ‘favours intervention’ suggests lower heavy alcohol use in the intervention group compared to the control group. The overall pooled estimate was not calculated as the subgroups differ by design and a decision was made a priori to report subgroups separately

There was moderate quality evidence from six studies for a short-term benefit in relation to heavy alcohol use (binge drinking) from school-based universal interventions (OR 0.70, [95% CI: 0.50 to 0.99]; 6 studies; 23,907 participants; *I*^*2*^*=*80%). There was substantial statistical heterogeneity, largely driven by two studies [56] and [57], Fig. 3. It was not possible to meta-analyse other intervention and setting groups, however individual studies are reported where possible in Fig. 3.

### Tobacco use and heavy use

**Fig. 4:**
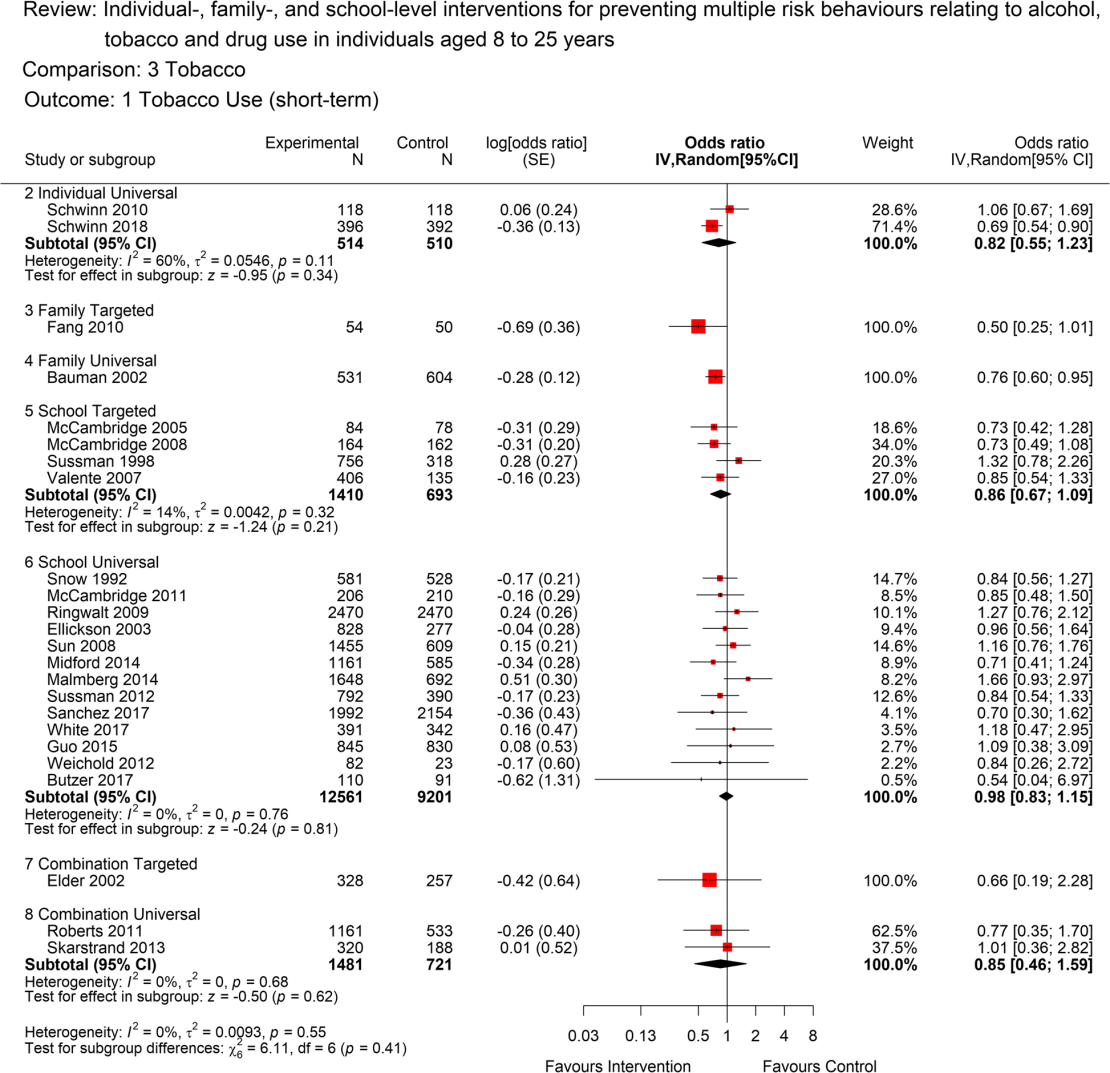
Forest plot for short term tobacco use(<12 months) by intervention level. The plot shows the data meta-analysed in intervention-level subgroups (school, family, individual, combination and universal or targeted) for the short-term (as close to 12 months post-intervention end as possible) tobacco use outcome. Red boxes represent point estimate (odds ratio) and horizontal lines the 95% confidence intervals. Black diamonds are pooled estimates for each subgroup. Estimates above 1 (on the right-hand side of the y-axis labelled ‘favours control’) suggests a lower tobacco use in the control group compared to the intervention group, while estimates below 1 (on the left-hand side of the y-axis labelled ‘favours intervention’ suggests lower tobacco use in the intervention group compared to the control group. The overall pooled estimate was not calculated as the subgroups differ by design and a decision was made a priori to report subgroups separately

Twenty-four studies contributed to the analyses for short term tobacco use (Fig. 4). There was moderate quality evidence that universal school based MRB interventions are likely to have no short-term benefit in relation to tobacco use (OR 0.98, [95% CI: 0.83 to 1.15]; 13 studies; 21,762 participants; *I*^2^=0%). The quality of evidence was rated as low for universal, individual-level (OR 0.82, 95% CI: 0.55 to 1.23]; 2 studies; 1024 participants; *I*^*2*^=60%) and universal, combined-setting interventions (OR 0.85, [95% CI: 0.46 to 1.59]; 2 studies; 2202 participants; *I*^*2*^=0%), and findings are consistent with both a beneficial, null and harmful effect of the intervention in the short term.

For targeted school-level interventions, there was moderate quality evidence from four studies of no, or a small beneficial effect for short-term tobacco use (OR 0.86, [95% CI: 0.66 to 1.11]; 4 studies; 2103 participants; *I*^*2*^=14%). There was only one study each in universal, family-level and targeted, family-level and combination groups, so these could not be meta-analysed but are shown on the forest plot. Not enough studies were available to be able to evaluate heavy tobacco use.

### Cannabis and other illicit drug use

**Fig. 5:**
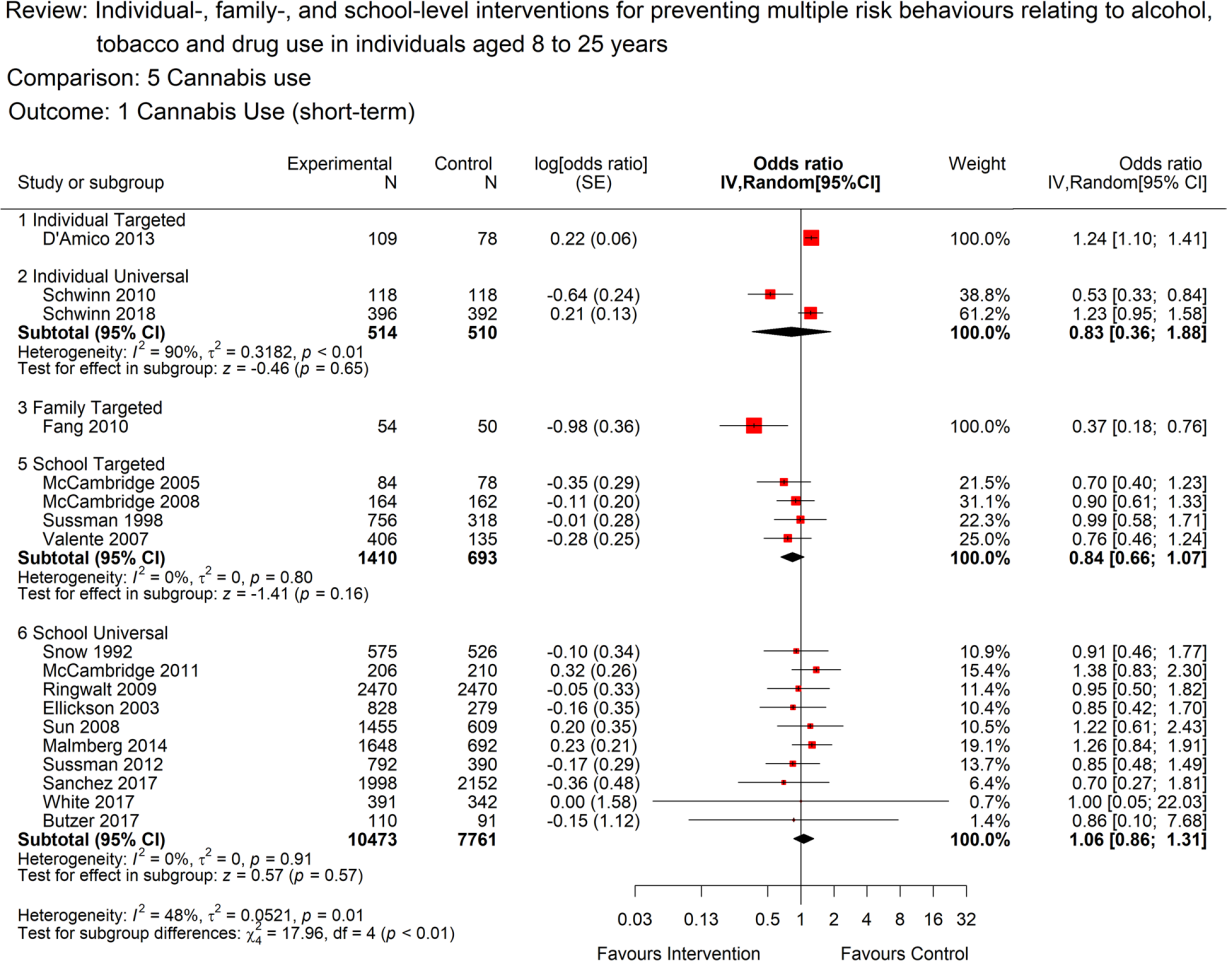
Forest plot for short term cannabis use (<12 months) by intervention level.The plot shows the data meta-analysed in intervention-level subgroups (school, family, individual, combination and universal or targeted) for the short-term (as close to 12 months post-intervention end as possible) cannabis use outcome. Red boxes represent point estimate (odds ratio) and horizontal lines the 95% confidence intervals. Black diamonds are pooled estimates for each subgroup. . Estimates above 1 (on the right-hand side of the y-axis labelled ‘favours control’) suggests a lower cannabis use in the control group compared to the intervention group, while estimates below 1 (on the left-hand side of the y-axis labelled ‘favours intervention’ suggests lower cannabis use in the intervention group compared to the control group. The overall pooled estimate was not calculated as the subgroups differ by design and a decision was made a priori to report subgroups separately

A total of 18 studies contributed to the analyses for cannabis use (Fig. 5). There was moderate quality evidence from universal, school-based interventions, to suggest little or no short-term benefit in relation to cannabis use (OR 1.06, [95% CI: 0.86 to 1.31]; 10 studies; 18,234 participants; *I*^2^=0%). There was high heterogeneity arising from two conflicting studies of universal, individual-based interventions (OR 0.83 [95% CI: 0.36 to 1.88]; 2 studies, 1024 participants; *I*^*2*^=90%). For targeted, school-level interventions, there was low quality evidence to support a short-term benefit for cannabis use (OR 0.84, [95% CI: 0.66-1.07]; 4 studies, 2103 participants; *I*^*2*^=0%). There was only one study in the targeted, individual-based and family-based (and none in the remaining groups), so these could not be meta-analysed but are included on the forest plot for visual comparison.

**Fig. 6:**
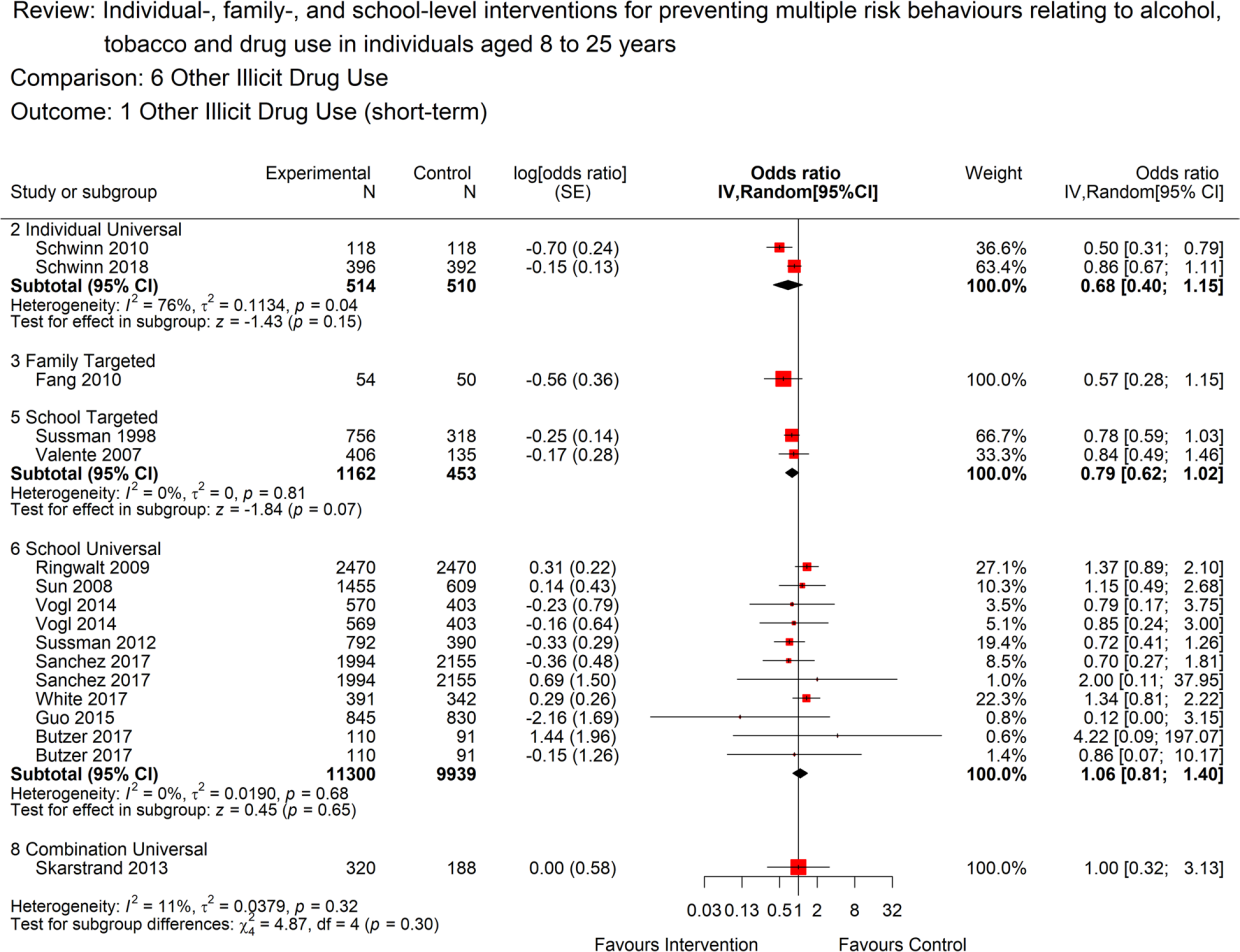
Forest plot for short term illicit drug use(<12 months) by intervention level.The plot shows the data meta-analysed in intervention-level subgroups (school, family, individual, combination and universal or targeted) for the short-term (as close to 12 months post-intervention end as possible) illicit drug use outcome. Red boxes represent point estimate (odds ratio) and horizontal lines the 95% confidence intervals. Black diamonds are pooled estimates for each subgroup. Estimates above 1 (on the right-hand side of the y-axis labelled ‘favours control’) suggests a lower illicit drug use in the control group compared to the intervention group, while estimates below 1 (on the left-hand side of the y-axis labelled ‘favours intervention’ suggests lower illicit use in the intervention group compared to the control group. The overall pooled estimate was not calculated as the subgroups differ by design and a decision was made a priori to report subgroups separately

Seventeen studies were included in the forest plot for other illicit drug use (Fig. 6). There was moderate quality evidence to suggest that universal, school-based interventions had little or no short-term benefit for other illicit drug use (OR 1.09, [95% CI: 0.85 to 1.39]; 11 studies; 21,239 participants; I^2^=0%). There is a suggestion of a benefit of universal, individual-level interventions in relation to other illicit drug use, however, this is based on only two studies judged to provide very low certainty evidence (OR 0.68, [95% CI: 0.40 to 1.15]; 2 studies; *n*=1024 participants; I^2^=76%). Two targeted, school-level studies also provided low quality evidence of a potential short-term benefit for other illicit drugs (OR 0.79, [95% CI: 0.62 to 1.02]; 2 studies; 1615 participants; I^2^=0%). There was only one study in universal, combination and targeted, family-level subgroups (and no studies in the remaining subgroups) so these could not be meta-analysed but are included on the forest plot.

### Longer-term intervention effects (longest follow-up available over 1 year)

Fewer studies included longer term follow-ups and statistical heterogeneity within groups was greater than observed for short-term outcomes. Pooled results suggest there was no benefit, on average, for universal, school-based interventions in relation to alcohol use (OR 1.01, 95% CI: 0.84 to 1.23]; n=6 studies; n=28,840 participants,; I^2^=65%) or tobacco use (OR 1.00, [95% CI 0.75 to 1.35]; n=5 studies, n=28,030 participants; I^2^=65%), and potential benefit in relation to cannabis use (OR 0.68, [95% CI 0.42 to 1.09]; studies; n=23,228 participants; I^2^=45%). Only two studies reported universal, school-based interventions that measured longer-term binge drinking, with suggestion of a beneficial effect (OR 0.77, [95% CI: 0.47 to 1.27]; 2 studies; 9529 participants; *I*^*2*^=82%). Only two studies [58, 59] investigated targeted, school-based interventions and there was an absence of evidence of effectiveness, due to wide confidence intervals, across all substance-use outcomes (see Additional file 9 for complete statistics). Other subgroups with single studies (or no studies) could not be meta-analysed but are included on the forest plots reported in Additional file 9.

### Sensitivity analyses and assessment of small study effects

Sensitivity analyses exploring the impact of larger or smaller imputed ICCs did not alter the findings reported above (Additional file 8). Funnel plots are reported for universal, school-based interventions for short-term alcohol use (*n*=15), tobacco use (*n*=13) and illicit drug use (*n*=11) outcomes. There was no evidence to suggest possible small study or publication bias, however, we note the small number of studies included (Additional file 10).

### Secondary outcomes

Secondary outcomes were sparsely and inconsistently reported, and meta-analysis was not possible. Additional file 11 contains a table showing all the relevant secondary outcomes reported for each study, as defined by study author.

## Discussion

Overall, we found insufficient evidence to support the effectiveness of multi-substance use interventions to prevent substance use-specific behaviours. In most analyses, we observed the point estimates were in the direction of a small beneficial effect, but with most confidence intervals crossing null.

Universal, school-level interventions were the most common intervention type included (*n*=41 across all substance use outcomes). However, there was evidence of no or only very small benefit of effectiveness on substance use outcomes, whether measured at either short- or long-term follow-up. There was some evidence of benefit for universal, family- and individual-level interventions, however with only two studies contributing to the meta-analysis of each substance use outcome, and the considerable between study heterogeneity observed in the universal, individual-level analyses, caution should be exercised when drawing conclusions here.

Targeted, school-level interventions were the next most reported intervention type (*n*=8 across all substance use outcomes). However, there was also inconclusive evidence of an effect across each substance use outcome, at both short- and longer-term follow-ups. There were not enough individual-, family- or combination- interventions on targeted populations to be able to evaluate these groups.

This review contributes to the mixed evidence base observed from other reviews examining the impact of multi-substance use interventions. For instance, our findings support a meta-analysis evaluating universal school-based interventions (*n*=53), which showed mixed evidence of effectiveness in relation to alcohol and drug use outcomes [60]. An overview of 21 systematic reviews, found a small but consistent positive effect of school-based prevention programmes for alcohol and drug use [32]. Further, a systematic review of universal school-based interventions (*n*=13) that combined student and parent based programmes, found there was potential efficacy of these types of interventions in preventing and reducing alcohol and drug use among adolescents [31]. Finally, in our companion Cochrane review assessing a wider range of risk behaviours [24], colleagues found that universal school-based programmes targeting substance use in combination with other MRB were more beneficial than we found in the present review, in relation to tobacco use and alcohol use, and that such interventions may be effective for illicit drug use [24]. Although the direction of intervention effects in our analyses is consistent with those observed in the Cochrane review, our summary effect estimates are smaller, with confidence intervals often spanning no intervention effect. The meta-analytic approach taken is similar across both reviews, however the present review includes almost twice the number of studies and three times as many participants. An unanswered question, in either review, is whether interventions that address prevention of non-substance and substance-use behaviours concurrently have greater benefit for individual outcomes. In both reviews, MRB outcomes were analyzed separately, ignoring the correlation that may be present between outcomes within studies and at a population level [61]. Future work, exploiting advances in multivariate meta-analysis, might address whether interventions which prevent one MRB (e.g. smoking) are effective for preventing another MRB (e.g. anti-social behaviour).

Notably, none of these reviews included all studies by intervention level and population, with the exception of the companion Cochrane review [24]. Our review contributes to the evidence base here as, in public health terms, there are unique assets and challenges to universal and targeted approaches [62]. We know from the Rose hypothesis [63] that while targeted approaches may have greater individual benefit to those in the high risk group, as ‘risk’ is normally distributed, universal approaches are likely to be more effective on a population level. Further, Rose’s prevention paradox [63] means these universal approaches are likely to show little individual benefit. Adopting the prevention paradox means that despite the effect being small for universal interventions, this may still be the preferable health prevention approach. Having conducted separate meta-analyses for intervention and setting means we can begin to assess what types of intervention are likely to be effective for adolescent substance use behaviours. The present review contributes to an emerging evidence base that universal interventions hold the greatest promise for prevention of MRBs [24, 64]. However, the effect sizes observed across all analyses in our review were small and, on balance, between-study heterogeneity was moderate, especially for the longer term timepoints. As such, we were unable to pinpoint specific intervention features that might be of interest. Using advanced synthesis methods [65], further work to formally evaluate the MRB intervention characteristics associated with larger effect sizes is needed. For example, a component network meta-analysis approach could be used to understand which intervention elements could be driving the effects of universal interventions [66, 67].

Most of the studies included in the review did not report their findings by characteristics known to be associated with equity or wider determinants of health. Using the studies included in the present review and the companion Cochrane review, we undertook a subgroup analysis to investigate whether intervention effects differed by socioeconomic status of participants [68]. However, there were too few studies that measured the appropriate demographic characteristics and therefore the study lacked power. There is a need for routine reporting of equity data so that stronger evidence of effect by SES and race/ethnicity can be demonstrated and that interventions can be evaluated for their impact on health inequalities [68, 69]. Moreover, given most studies were conducted in high income countries, predominantly the USA, there is a need for further research in different cultural, geographic and socioeconomic contexts. For instance, there is a distinct lack of studies conducted in the Middle East, Africa and South America, which is the case for both adolescent and adult populations [70]. This is of particular relevance to adolescent substance use behaviours, given that the majority of the world’s young people aged 10-24 years live in low- and middle-income countries, and there are high rates of tobacco-, drug- and alcohol-use within these regions [71–74]. It is critical we consider the global context related to adolescent health and wellbeing to develop and assess context-appropriate multiple risk behaviour interventions in areas with the greatest need.

### Strengths and Limitations

This systematic review is among the first to quantitatively assess interventions targeting multiple substance use behaviours in young people. This review involved a comprehensive search and review process, across an extensive number of databases. We included universal and targeted populations and a wide range of interventions, delivered at individual-, family-, school- and combination-levels, yet analysed separately by population and setting to reflect the heterogeneity in design.

The findings are limited by the large proportion of studies that were classified as unclear or high risk of bias due to the lack of blinding and lack of clarity surrounding allocation concealment or reporting. Within some of our intervention subgroups (family targeted, and universal and targeted individual), there were too few studies with useable data and therefore findings are restricted. We were also unable to meta-analyse the results by other potentially meaningful distinctions aside from intervention level. For example, it was often unclear whether studies were adopting a purely prevention approach (to young people uninitiated to any substance use behaviours), a harm reduction approach (to those already engaging in substance use behaviours), or both. With greater consistency of reporting across individual studies, future reviews will be able to interrogate the results more comprehensively and compare different approaches.

Our review defined young people within a wide age range of 8-25 years in an attempt to capture the adolescent period, with some allowance for interventions that started earlier but ran into adolescence and studies that followed up participants into their mid-twenties. This broad age range presents a challenge in interpreting our data as young, middle and older adolescents have been viewed as occupying different developmental and social life stages [75]. Older and younger adolescents will have very different experiences with substance use behaviours as well as differing levels of independence, peer influence and access to adult privileges (e.g. driving, legal alcohol consumption) [75, 76], suggesting they may respond to interventions differently. Although the majority of included studies focused on adolescents aged 11-14 years, our meta-analyses include some older and younger adolescents. With more detailed reporting of demographic characteristics in the individual studies, in future work we would be able to separate the studies into more focused age groups.

The majority of included studies had a cluster randomisation design, however a large proportion did not transparently report adjustment for clustering in their analyses. Not accounting for correlation within clusters can lead to inflated and over-precise estimates of effect. Our retrospective estimation of ICC and use of robust standard errors for meta-analyses is likely to have provided more conservative precision estimates, which in turn will have contributed to wider confidence intervals. We note however, that the sensitivity analyses did not change our conclusions. Our most recent search was undertaken in October 2019, so there may be relevant studies missing from our analysis. The COVID-19 pandemic has affected capacity to update the searches.

Between-study heterogeneity was substantial in some analyses, but surprisingly low in others. The challenges we experienced relating to heterogeneity are common when reviewing public health interventions of this nature, which highlights the debate surrounding whether RCTs and systematic reviews are the most appropriate methods for measuring the impact of complex public health interventions, particularly if multiple components are involved [77]. There are also limitations to using GRADE to assess certainty of evidence from complex public health interventions, which have been debated [78–80]. We would not expect complex interventions to have a rating of 'high' certainty, due to the non-standardised nature of such interventions and heterogeneity [78].

## Conclusion

Our systematic review and meta-analysis suggests universal and targeted interventions of different types of setting have no or small benefit in preventing multiple risk behaviours relating to alcohol, tobacco and drug use in young people aged 8-25 years. However, small benefits of universal interventions in trials can have high public health benefit, so universal interventions warrant further investigation. The heterogeneity within some subgroups suggests future work could explore combinations of interventions and components that may be more effective than others. Methodologically robust trials and improved and more consistent reporting within individual studies are needed to allow for better evidence syntheses.

## Supplementary Information

**Additional file 1. **Search strategy.

**Additional file 2. **Details of excluded and ongoing studies.

**Additional file 3. **Excluded studies from meta-analysis.

**Additional file 4. **Details of data transformations.

**Additional file 5. **Outcome measures.

**Additional file 6. **Planned subgroup analysis.

**Additional file 7.** Table of characteristics.

**Additional file 8. **Sensitivity analyses.

**Additional file 9. **Forest plots for long term substance use outcomes.

**Additional file 10. **Funnel plots.

**Additional file 11.** Secondary outcomes.

## Data Availability

The datasets used and/or analysed during the current study are available from the corresponding author on reasonable request.

## Abbreviations

MRB: Multiple Risk Behaviour
PRISMA: Preferred Reporting Items for Systematic Reviews and Meta-Analyses
N: Number
OR: Odds Ratio
CI: Confidence Interval
RCT: Randomised Controlled Trial
GRADE: Grades of Recommendation, Assessment, Development and Evaluation
SMD: Standard Mean Difference
SE: Standard Error
logOR: log Odds Ratio
ICC: Intracluster Correlation Coefficient
