# Supplementary material for: Individual-, family- and school-based interventions to prevent multiple risk behaviours relating to alcohol, tobacco and drug use in young people aged 8-25 years: a systematic review and meta-analysis
Source: BMC Public Health. 2022 Jun 3;22:1111. doi: 10.1186/s12889-022-13072-5 (PMC9165543; doi:10.1186/s12889-022-13072-5)
Supplement: Supplementary file 1 — Additional file 1. Search strategy. [file 12889_2022_13072_MOESM1_ESM.docx]

**Additional File 1 – Search strategy**

The following databases were searched between May 6^th^ and 15^th^ in 2012 for the full range of dates available in each database (no date restrictions were imposed):

- Australian Education Index (ProQuest) ‐ 1979 to current.
- Bibliomap ‐ database of health promotion research (<http://eppi.ioe.ac.uk/webdatabases/Search.aspx>).
- British Education Index (ProQuest) ‐ 1975 to current.
- Campbell Library (<http://www.campbellcollaboration.org/lib/>).
- Cumulative Index to Nursing and Allied Health Literature (CINAHL) (Ovid) ‐ 1950 to present.
- Clinicaltrials.gov (<https://clinicaltrials.gov/>).
- Cochrane Central Register of Controlled Trials (CENTRAL) ‐ 1950 to 2015.
- Dissertation Express ‐ cutdown versions of dissertation abstracts (<http://dissexpress.umi.com/dxweb/search.html>).
- Database of Promoting Health Effectiveness Reviews (DoPHER) (<http://eppi.ioe.ac.uk/webdatabases4/Search.aspx>).
- Embase (Ovid) ‐ 1974 to 2015, week 16.
- Education Resources Information Centre (ERIC; ProQuest) ‐ 1966 to current.
- EThOS – British Library electronic theses online (<http://ethos.bl.uk/AdvancedSearch.do?new=1>).
- International Bibliography of the Social Sciences: Politics & Economics (ProQuest). 1950 to 2015.
- MEDLINE (Ovid) ‐ 1950 to 6 May 2015.
- PsycINFO (Ovid) ‐ 1806 to 2015, week 17.
- Sociological Abstracts (CSA) ‐ 1952 to current

Following MacArthur et al [1], and in line with Cochrane guidance [2], we reduced the number of databases for the update searches to those which had returned the most relevant studies in the first search iteration and those which returned studies already returned by other databases (i.e. duplicates). For the update searches in 2018 and 2019 the database selection was streamlined to the following eight databases:

- Australian Education Index (ProQuest): 1/1/2012 to 26/6/2018
- British Education Index (EBSCOhost): 1/1/2012 to 26/6/2018
- Cumulative Index to Nursing and Allied Health Literature (CINAHL) (Ovid): 1/1/2012 to 3/7/2018
- Cochrane Central Register of Controlled Trials (CENTRAL): 1/1/2012 to 20/6/18
- Embase (Ovid): 1/1/2012 to 19/6/18
- Education Resources Information Centre (EBSCOhost): 1/1/2012 to 3/7/18
- MEDLINE (Ovid): 1/1/2012 to 2/5/18
- PsycINFO (Ovid): 1/1/2012 to 13/6/18

NB: the 2019 searches were conducted between 24/09/19 and 30/10/19.

For the update searches, we also worked with an information specialist to refine the terms and optimise the search strategy. The specialist noted the specificity of the original search strategy meant that 400 papers had been screened for each study eventually included in the MacArthur review [1]. The specialist has specific expertise in evidence synthesis and systematic review and made two suggestions for search optimisation: (1) we use a specific RCT search filter to improve the accuracy and recall of RCT studies and (2) remove redundant terms from the strategy. For comparison, the original 2012 Medline search retrieved 3609 references across all years (inception to 8^th^ May 2018) and the 2018 optimised search retrieved 2228 references across all years. This is a 38% decrease in references to screen. The sensitivity of the new search strategy was then also tested on the 94 studies initially identified for inclusion across the present review and the sibling Cochrane review, in 2012. Of these 94 studies, 76 were indexed on Medline at the time of search optimisation (8/5/18). The specialist compared the recall rate of the 2012 (original) and 2018 (optimised) strategy in Medline. Of these 76 references, the original 2012 search retrieved 60 records (79% recall) and the optimised 2018 search retrieved 68 (89% recall). It was concluded that fewer references were retrieved by the optimised search (increased specificity) and with greater recall (increased sensitivity). The complete 2012 and 2018 search strategies are reproduced here for Medline only. The strategies for other databases are available from the corresponding author.

## 2018: optimised Medline search strategy

Ovid MEDLINE(R) Epub Ahead of Print, In-Process & Other Non-Indexed Citations, Ovid MEDLINE(R)

Daily and Ovid MEDLINE(R) <1946 to 2-May-2018>

1 controlled clinical trial.pt. (92378)

2 randomized controlled trial.pt. (460041)

3 (randomi#ed or randomi#ation or randomi#ing).ti,ab,kf. (539175)

4 trial.ab,ti,kf. (505256)

5 (RCT or "at random" or (random* adj3 (administ* or allocat* or assign* or class* or cluster* or

control* or crossover or cross-over or design* or determine* or divide* or division or distribut* or

expose* or fashion or number* or place* or recruit* or subsitut* or treat*))).ti,ab,kf. (484102)

6 clinical trials as topic.sh. (183493)

7 or/1-6 (1249982)

8 exp animals/ not humans.sh. (4452141)

9 7 not 8 (1154969)

10 CHILD/ (1560505)

11 ADOLESCENT/ (1859979)

12 SCHOOLS/ (32590)

13 SCHOOL HEALTH SERVICES/ (15951)

14 STUDENTS/ or UNIVERSITIES/ (70987)

15 (child* or boy* or girl* or kids or juvenil* or minors or paediatric* or pediatric* or adolesc* or

preadolesc* or pre-adolesc* or pubert* or pubescen* or prepube* or pre-pube* or teen* or (young adj

(adult* or people or patient* or men* or women* or male or female or survivor* or offender* or

minorit*)) or youth* or student* or undergrad*).ti,ab,kf. (2062470)

16 (child* or adolesc*).jw. (166029)

17 or/10-16 (3594798)

18 DRINKING BEHAVIOR/ (6509)

19 ALCOHOL DRINKING/ or BINGE DRINKING/ or ALCOHOL DRINKING in COLLEGE/ or UNDERAGE

DRINKING/ (62480)

20 ((binge or binging or problem*) adj2 drink*).ti,ab,kf. (8534)

21 (alcohol or beer or cider or wine or spirit? or alcopop?).mp. (297122)

22 or/18-21 (304555)

23 exp SMOKING/ (135781)

24 "Tobacco Use Disorder"/ (10282)

25 Tobacco/ or Tobacco Products/ (30666)

26 (smoking or smokers or tobacco or cigarette* or nicotine).mp. (343264)

27 or/23-26 (343267)

28 exp SUBSTANCE RELATED DISORDERS/ (257242)

29 MARIJUANA SMOKING/ (4052)

30 MARIJUANA ABUSE/ (5564)

31 DRUG-SEEKING BEHAVIOR/ (967)

32 (drug? or substance? or cannabi* or marijuana or marihuana or hash or hashish or skunk or

ganja or sinsemilia or aerosol* or inhalant? or solvent? or stimulant? or legal high? or barbituat* or

benzodiazepin* or opioid? or opiate? or narcotic? or psychoactive* or psychostimulant? or

hallucinogen* or psychedelic* or ecstasy or painkiller? or pain killer?).mp. (3210734)

33 or/28-32 (3299521)

34 (22 and 27) or (22 and 33) or (27 and 33) (209652)

35 prevention & control.fs. (1177785)

36 (deterr* or prevent*).ti,kf. (296496)

37 HEALTH EDUCATION/ (57559)

38 HEALTH PROMOTION/ (66221)

39 PRIMARY PREVENTION/ (16710)

40 SECONDARY PREVENTION/ (17948)

41 ((primary or secondary or universal or select*) adj2 prevention).ti,ab,kf. (34295)

42 ((universal or selective) adj2 (intervention or program or project)).ti,ab,kf. (1125)

43 PILOT PROJECTS/ (106152)

44 PROGRAM EVALUATION/ (56419)

45 FAMILY THERAPY/ (8456)

46 (((life or decision making or refusal) adj skills) or ((assertion or skills) adj training) or goal

setting).ti,ab,kf. (10132)

47 ((communit* or family or families or mentor* or peer or peers or ((school or classroom) adj

based)) adj3 (intervention or program or project)).ti,ab,kf. (27252)

48 capacity building.ti,ab,kf. (3514)

49 (mental health adj3 promot*).ti,ab,kf. (1931)

50 PROBLEM SOLVING/ (23393)

51 (preventi* adj3 (curriculum or intervention or program* or project)).ti,ab,kf. (42547)

52 ((parent* or guardian? or peer) adj2 (intervention or program* or project)).ti,ab,kf. (5981)

53 ((education* or psychoeducat*) adj2 (intervention or program* or project)).ti,ab,kf. (49423)

54 (brief intervention or single session).ti,ab,kf. (6951)

55 ALCOHOL DRINKING/**pc** (4041)

56 SMOKING PREVENTION/ or SMOKING CESSATION/ or “TOBACCO USE CESSATION"/ (37534)

57 exp SUBSTANCE-RELATED DISORDERS/**pc** (20638)

58 MARIJUANA SMOKING/**pc** (243) *[Note to self, these lines are redundant, see line 35]*

59 (((substance or drug?) adj (use* or abuse) adj2 prevention) or drug? education).ti,ab,kf.

(2010)

60 or/35-59 (1648101)

61 *SUBSTANCE-RELATED DISORDERS/pc (4773)

62 9 and 17 and 34 and 60 (2064)

63 9 and 17 and 61 (386)

64 62 or 63 (2228)

## 2012: original Medline search strategy

Search strategy for Ovid MEDLINE (OvidSP), 1950 to 24 May 2012.

1 ("Health risk behavio?r*" or "multiple risk behavio?r*" or "high risk behavio?r*" or "multiple risk factor*" or "behavio?r* risk factor*").mp. [mp=title, abstract, original title, name of substance word, subject heading word, protocol supplementary concept, rare disease supplementary concept, unique identifier]

2 Dangerous Behavior/

3 Risk‐Taking/

4. 1 or 2 or 3

5 "Tobacco Use Disorder"/

6 Smoking/

7 smoking.mp. [mp=title, abstract, original title, name of substance word, subject heading word, protocol supplementary concept, rare disease supplementary concept, unique identifier]

8 ((tobacco or cigarette* or nicotine) adj3 (addict* or use* or usage or using or intake or consum*)).mp. [mp=title, abstract, original title, name of substance word, subject heading word, protocol supplementary concept, rare disease supplementary concept, unique identifier]

9. 5 or 6 or 7 or 8

10 exp Drinking Behavior/

11 exp Alcohol‐Related Disorders/

12 ((alcohol* or ethanol or beer or cider or wine or spirit* or alcopop*) adj3 (use* or usage* or using or intake or consum* or drink* or misus* or abus*)).mp. [mp=title, abstract, original title, name of substance word, subject heading word, protocol supplementary concept, rare disease supplementary concept, unique identifier]

13 ((alcohol* or drink* or ethanol) adj3 (excess* or binge* or binging or intoxicat* or poison* or risk* or depend*)).mp. [mp=title, abstract, original title, name of substance word, subject heading word, protocol supplementary concept, rare disease supplementary concept, unique identifier]

14. 10 or 11 or 12 or 13

15 cannabis/ or exp street drugs/ or marijuana smoking/

16 Drug‐Seeking Behavior/

17 Substance‐Related Disorders/

18 ((marijuana or cannabis or recreational drug* or class c or white widow*) adj2 (abus* or use* or using or usage or misus* or smok* or addict* or depend*)).mp. [mp=title, abstract, original title, name of substance word, subject heading word, protocol supplementary concept, rare disease supplementary concept, unique identifier]

19 substance abuse, intravenous/

20 (class c adj2 (abus* or addict* or depend* or misus* or use* or usage or using)).mp. [mp=title, abstract, original title, name of substance word, subject heading word, protocol supplementary concept, rare disease supplementary concept, unique identifier]

21 (substance* adj2 (abus* or addict* or depend* or inject* or intravenous or misus* or use* or usage or using)).mp. [mp=title, abstract, original title, name of substance word, subject heading word, protocol supplementary concept, rare disease supplementary concept, unique identifier]

22 ((Class a or class b or drug* or cocaine or ecstasy or mdma or glue or gas or aerosol* or solvent* or magic mushroom* or crack or white widow* or ketamine or heroin or morphine or narcotic* or opiat* or opioid* or popper* or lsd or methamphetamine* or amphetamine*) adj2 (abus* or addict* or depend* or inhal* or misus* or sniff* or use* or using or usage)).mp. [mp=title, abstract, original title, name of substance word, subject heading word, protocol supplementary concept, rare disease supplementary concept, unique identifier]

23 (inhal?nt* adj2 (abus* or addict* or depend* or misus* or sniff* or use* or using or usage)).mp. [mp=title, abstract, original title, name of substance word, subject heading word, protocol supplementary concept, rare disease supplementary concept, unique identifier]

24. 15 or 16 or 17 or 18 or 19 or 20 or 21 or 22 or 23

25 (gambl* or betting).mp. [mp=title, abstract, original title, name of substance word, subject heading word, protocol supplementary concept, rare disease supplementary concept, unique identifier]

26 Safe sex/ or unsafe sex/ or sexual behavior/ or sexual abstinence/ or exp contraceptive devices/ or contraceptive agents/ or exp contraception/ or exp reproductive behavior/ or sexual partners/

27 (risky sex* or unsafe sex* or safe* sex* or sexual intercourse or reproductive behavio?r* or sexual behavio?r* or sexual health).mp. [mp=title, abstract, original title, name of substance word, subject heading word, protocol supplementary concept, rare disease supplementary concept, unique identifier]

28 (contracept* or condom? or morning after pill*).mp. [mp=title, abstract, original title, name of substance word, subject heading word, protocol supplementary concept, rare disease supplementary concept, unique identifier]

29. 26 or 27 or 28

30 exp crime/ or juvenile delinquency/ or social behaviour disorders/

31 violence/ or exp aggression/ or wounds, stab/

32 (delinquen* or offen* or reoffend* or violen* or theft* or robbery or burglary* or steal* or criminal damage or joyrid* or joy‐rid* or assault* or (sell* adj drug*) or devian* or anti‐social behavio?r* or antisocial behavio?r* or graffiti or racist abuse or index crime* or (breaking adj entering) or strong‐arming or pan‐handling or panhandling or disorderly conduct or prostitut* or (carry* adj2 weapon*) or (buy* adj2 stolen) or criminal behavio?r* or (noisy adj1 rude) or (nuisance* adj2 neighbour*) or fight* or stab? or stabbing or stabbed or stabwound* or wound* or aggress* or weapon* or knife* or knives or gun* or firearm* or murder*).mp. [mp=title, abstract, original title, name of substance word, subject heading word, protocol supplementary concept, rare disease supplementary concept, unique identifier]

33 ((youth* or street or criminal* or adolescen* or juvenile* or teen*) adj2 gang?).mp. [mp=title, abstract, original title, name of substance word, subject heading word, protocol supplementary concept, rare disease supplementary concept, unique identifier]

34 ((adolescen* or youth* or juvenile* or delinquen* or teen* or gang? or school* or college* or sixth form*) adj2 (crim* or offen* or violen* or fight*)).mp. [mp=title, abstract, original title, name of substance word, subject heading word, protocol supplementary concept, rare disease supplementary concept, unique identifier]

35. 30 or 31 or 32 or 33 or 34

36 Head protective devices/ or exp accident prevention/ or risk reduction behavior/

37 ((bicyc* or cycl* or bik* or motorbike* or motorcycl*) adj2 ((helmet* or protect* or risk reduc* or head gear or head protection) adj3 (lack* or no? or without or absen* or wear*))).mp. [mp=title, abstract, original title, name of substance word, subject heading word, protocol supplementary concept, rare disease supplementary concept, unique identifier]

38 Seat Belts/

39 ((Use* or using or usage or wear* or wore) adj2 (seat‐belt* or seatbelt* or safety belt*)).mp. [mp=title, abstract, original title, name of substance word, subject heading word, protocol supplementary concept, rare disease supplementary concept, unique identifier]

40 (injur* adj2 behav*).mp. [mp=title, abstract, original title, name of substance word, subject heading word, protocol supplementary concept, rare disease supplementary concept, unique identifier]

41 ((alcohol* or intoxica* or dr?nk*) adj2 (driv* or vehicle* or motor* or car? or van? or automobile* or auto mobile*)).mp.

42. 36 or 37 or 38 or 39 or 40 or 41

43 suicide, attempted/

44 ((suicid* or harm* or injur* or hurt*) adj2 (gesture* or behavio?r*)).mp. [mp=title, abstract, original title, name of substance word, subject heading word, protocol supplementary concept, rare disease supplementary concept, unique identifier]

45 (para‐suicid* or parasuicid* or attempt* suicid* or suicid* attempt*).mp. [mp=title, abstract, original title, name of substance word, subject heading word, protocol supplementary concept, rare disease supplementary concept, unique identifier]

46 ((non fatal or nonfatal) adj2 (suicid* or harm*)).mp. [mp=title, abstract, original title, name of substance word, subject heading word, protocol supplementary concept, rare disease supplementary concept, unique identifier]

47 Self‐injurious behavior/ or self mutilation/

48 ((injur* or mutil* or harm* or wound* or hurt*) adj2 (self or themsel* or yoursel*)).mp. [mp=title, abstract, original title, name of substance word, subject heading word, protocol supplementary concept, rare disease supplementary concept, unique identifier]

49 dsh.tw. or selfinflict*.mp. or self inflict*.mp. or selfinjur*.mp. or selfharm*.mp. or selfmutilat*.mp. or self destructive behavio?r*.mp. [mp=title, abstract, original title, name of substance word, subject heading word, protocol supplementary concept, rare disease supplementary concept, unique identifier]

50 43 or 44 or 45 or 46 or 47 or 48 or 49

51 exp diet/ or hyperphagia/ or dietary fats/

52 ((calori* or fat? or fatty or fizz* or soft* or carbonated* or sweetened or salt* or sugar* or fruit* or veg? or vegetable* or fibre* or fiber* or 5‐a‐day or five a day or go for 2&5) adj2 (intake or food* or diet* or consum* or meal* or eat* or nutrition or drink* or snack*)).mp. [mp=title, abstract, original title, name of substance word, subject heading word, protocol supplementary concept, rare disease supplementary concept, unique identifier]

53 ((poor or over* or unhealthy or health*) adj3 (nutrition or diet* or eat* or meal* or food* or snack* or drink*)).mp. [mp=title, abstract, original title, name of substance word, subject heading word, protocol supplementary concept, rare disease supplementary concept, unique identifier]

54 51 or 52 or 53

55 exp exercise/ or physical exertion/ or physical fitness/ or physical endurance/ or fitness/

56 Baseball/ or basketball/ or bicycling/ or boxing/ or dancing/ or football/ or gardening/ or golf/ or gymnastics/ or hockey/ or exp martial arts/ or mountaineering/ or exp racquet sports/ or exp running/ or skating/ or snow sports/ or soccer/ or exp swimming/ or volleyball/ or walking/ or weight lifting/ or wrestling/

57 (Physical activity or fitness or physical* fit* or physical exert* or exercise or aerobic activit* or sport* or aerobic capacity or active lifestyle* or outdoor activit* or gym* or mvpa).mp. [mp=title, abstract, original title, name of substance word, subject heading word, protocol supplementary concept, rare disease supplementary concept, unique identifier]

58 ((fitness or leisure) adj2 (class* or regime* or program* or centre* or center*)).mp. [mp=title, abstract, original title, name of substance word, subject heading word, protocol supplementary concept, rare disease supplementary concept, unique identifier]

59 ((fit* or sport* or activ* or exercise or physical exer*) adj3 (lack* or low or no or absen*)).mp. [mp=title, abstract, original title, name of substance word, subject heading word, protocol supplementary concept, rare disease supplementary concept, unique identifier]

60. 55 or 56 or 57 or 58 or 59

61 Sedentary lifestyle/ or Video games/

62 gaming.mp.

63 ((view* or watch* or play* or game* or gaming or use* or using or usage) adj2 (television or tv or video* or dvd* or screen or comput* or laptop* or media)).mp. [mp=title, abstract, original title, name of substance word, subject heading word, protocol supplementary concept, rare disease supplementary concept, unique identifier]

64 ((screen or sedentary or view*) adj2 (time or hour* or minute*)).mp. [mp=title, abstract, original title, name of substance word, subject heading word, protocol supplementary concept, rare disease supplementary concept, unique identifier]

65 ((inactiv* or seden* or indoor*) adj3 (lifestyle* or activit*)).mp. [mp=title, abstract, original title, name of substance word, subject heading word, protocol supplementary concept, rare disease supplementary concept, unique identifier]

66. 61 or 62 or 63 or 64 or 65

67 (4 and 9) or (4 and 14) or (4 and 24) or (4 and 25) or (4 and 29) or (4 and 35) or (4 and 42) or (4 and 50) or (4 and 54) or (4 and 60) or (4 and 66) or (9 and 14) or (9 and 24) or (9 and 25) or (9 and 29) or (9 and 35) or (9 and 42) or (9 and 50) or (9 and 54) or (9 and 60) or (9 and 66) or (14 and 24) or (14 and 25) or (14 and 29) or (14 and 35) or (14 and 42) or (14 and 50) or (14 and 54) or (14 and 60) or (14 and 66) or (24 and 25) or (24 and 29) or (24 and 35) or (24 and 42) or (24 and 50) or (24 and 54) or (24 and 60) or (24 and 66) or (25 and 29) or (25 and 35) or (25 and 42) or (25 and 50) or (25 and 54) or (25 and 60) or (25 and 66) or (29 and 35) or (29 and 42) or (29 and 50) or (29 and 54) or (29 and 60) or (29 and 66) or (35 and 42) or (35 and 50) or (35 and 54) or (35 and 60) or (35 and 66) or (42 and 50) or (42 and 54) or (42 and 60) or (42 and 66) or (50 and 54) or (50 and 60) or (50 and 66)

68 child/ or adolescent/ or child, preschool/ or infant/

69 (school* or student* or child* or pupil* or infant*).tw.

70 (Adolescen* or teen* or young person or young people or youth* or hooligan* or young adult* or early adult* or juvenile* or minor? or emerging adult* or girl* or boy? or apprentice* or FE college* or young m#n or young wom#n or young male* or young female* or under 18* or sixth‐form* or secondary education or tertiary education or higher education or further education or preschool* or primary education or infan* or kid? or nurser* or playschool* or kindergarten* or prekindergarten*).mp.

71 (teacher* or parent* or guardian* or grandparent* or mother* or father* or mum? or dad? or maternal or paternal or nurse? or childminder* or child care provider* or playworker* or family or families or carer* or midwife or mid wife or midwives or mid wives).mp. [mp=title, abstract, original title, name of substance word, subject heading word, protocol supplementary concept, rare disease supplementary concept, unique identifier]

72. 68 or 69 or 70 or 71

73 (randomized controlled trial or controlled clinical trial).pt.

74 (randomi#ed or placebo* or randomly).ab.

75 trial.ti.

76 clinical trials as topic.sh.

77. 73 or 74 or 75 or 76

78 exp animals/ not humans.sh.

79. 77 not 78

80. 67 and 72 and 79

1. MacArthur, G., et al., *Individual‐, family‐, and school‐level interventions targeting multiple risk behaviours in young people.* Cochrane Database of Systematic Reviews, 2018(10). CD009927

2. Higgins, J.P. and S. Green. *Cochrane Handbook for Systematic Reviews of Interventions Version 5.1.0*. 2011 [cited 4 23/01/2018]; Available from: <www.handbook.cochrane.org>.
