# Supplementary material for: Individual-, family- and school-based interventions to prevent multiple risk behaviours relating to alcohol, tobacco and drug use in young people aged 8-25 years: a systematic review and meta-analysis
Source: BMC Public Health. 2022 Jun 3;22:1111. doi: 10.1186/s12889-022-13072-5 (PMC9165543; doi:10.1186/s12889-022-13072-5)
Supplement: Supplementary file 2 — Additional file 2. Details of excluded and ongoing studies. [file 12889_2022_13072_MOESM2_ESM.docx]

**Additional File 2 – Reasons for exclusions by update year and detail of ongoing and awaiting classification studies**

**Reasons for exclusions by update year**

**June 2012 (excluded studies at full text, n=218)**

Age-range (n=7)

Awaiting classification from authors (n=8)

Broader behaviour focus than tobacco, alcohol and drugs (e.g. included an additional risk behaviour such as anti-social behaviour) (n=80)

Clinical population or setting (n=9)

Community intervention (n=5)

Could not locate study paper (n=5)

Dissertation, abstract or poster (n=11)

Follow-up time period (n=17)

Not RCT (n=45)

Not behaviour engagement outcomes (e.g. intention outcomes) (n=6)

Not in English (n=2)

Not multiple risk behaviour (e.g. single use behaviour) (n=21)

Not outcome data (n=2)

**May 2018 (n=187)**

Age-range (n=16)

Awaiting classification from authors (n=11)

Broader behaviour focus than tobacco, alcohol and drugs (e.g. included an additional risk behaviour such as anti-social behaviour) (n=43)

Clinical population or setting (n=13)

Control arm (n=4)

Community intervention (n=1)

Dissertation, abstract or poster (n=3)

Follow-up time period (n=21)

Not RCT (n=60)

Not in English (n=2)

Not multiple risk behaviour (e.g. single use behaviour) (n=6)

Ongoing studies (n=7)

**October 2019 (n=87)**

Age-range (n=12)

Awaiting classification from authors (n=2)

Broader behaviour focus than tobacco, alcohol and drugs (e.g. included an additional risk behaviour such as anti-social behaviour) (n=28)

Clinical population or setting (n=8)

Control arm (n=7)

Follow-up time period (n=4)

Not RCT (n=13)

Not in English (n=1)

Not multiple risk behaviour (e.g. single use behaviour) (n=10)

Not outcome data (n=1)

Ongoing studies (n=1)

**Ongoing studies**

Two studies were identified as ongoing at the time of our meta-analysis, but which have now completed and would be eligible for inclusion. Below we provide the detail of the citation from our original searches and the subsequent publication:

1. **Original citation:** Johnson, E. J.Dickerson, D. L.Brown, R. A.Klein, D. J.Agniel, D. (2019). Motivational interviewing and culture for urban native American youth: Engagement, retention and RCT outcomes. *Alcoholism: Clinical and Experimental Research* 281A. (Conference abstract)

**Subsequent publication:** Elizabeth J.D'Amico EJ, Dickerson DL, Brown RA et al (2020) Motivational interviewing and culture for urban Native American youth (MICUNAY): A randomized controlled trial. *Journal of Substance Abuse Treatment.* 111; 86-99

1. **Original citation:** No author specified. Linking Families Together Study- A Randomized Trial to Raise Parental Monitoring. [Https://clinicaltrials.gov/show/nct02129153](https://clinicaltrials.gov/show/nct02129153) (trial registration)

**Subsequent publication:** Bergman P, Dudovitz RN, Dosanjh KK, & Wong MD (2019) Engaging Parents to Prevent Adolescent Substance Use: A Randomized Controlled Trial. *AJPH* 109; 1455-61.

**Awaiting classification**

PROSPER is a suite of adolescent multiple risk behaviour interventions, which includes substance use outcomes. Some of the papers listed below suggest the scope of PROSPER is broader than just prevention of substance use and may include other behaviours such as ‘delinquent’ or ‘anti-social’ risk behaviours. We are awaiting clarification from the study authors and in the interim chose to exclude the following papers, as we were unable to make a judgement based on the available information.

- Spoth R; Redmond C, Shin C (2001) Journal *of Consulting & Clinical Psychology.* 69: 627-42.
- Spoth R, Redmond C, Trudeau L, Shin C (2002) *Psychology of Addictive Behaviors.*16: 129-34.
- Spoth R et al (2004) Journal *of Consulting & Clinical Psychology.* 72: 535-42.
- Spoth R, Shin C, Guyll M, Redmond C, Azevedo K (2006). *Prevention Science.* 7: 209-24.
- Spoth R et al (2007) *American Journal of Preventive Medicine.* 32: 395-402.
- Spoth R et al(2008). *Drug and Alcohol Dependence.* 96: 57-68.
- Spoth R et al (2009) *Journal of Consulting & Clinical Psychology*. 77: 620-32.
- Spoth R et al (2011)  *American Journal of Preventive Medicine.*40: 440-447.
- Crowley, D. et al (2014) *Prevention Science.* 15: 246-255
- Crowley, D et al (2014) *Preventive Medicine.* 62: 71-7
- Redmond CS et al (2014) *The Journal of Primary Prevention*: 35 p.295
- Spoth, R. et al (2014) *Prevention Science*: 15; S47-S58
- Spoth, R et al (2012) *Journal of Adolescent Health*. 50; 414-7;
- Spoth, R et al (2013). *Preventive Medicine.* 56; 190-6
- Spoth, R et al (2017). *Psychological Medicine*. 47; 2246-2259
- Spoth, R et al (2013). *American Journal of Public Health*. 103; 665-72
- Spoth, R et al (2014) *Journal of Consulting & Clinical Psychology* 82; 949-63
- Spoth, R et al (2016) *Journal of Consulting & Clinical Psychology*. 84: 913-21
- Vandenbergh DJS et al (2016) *Nicotine & Tobacco Research* 18; 212-20
- Spoth R et al (2019) *Journal of Youth and Adolescence*. 444-458.  10.1007/s10964-018-0946-y
- Zheng Y (2019) *Dissertation Abstracts International: Section B: The Sciences and Engineering.*
