# Supplementary material for: Individual-, family- and school-based interventions to prevent multiple risk behaviours relating to alcohol, tobacco and drug use in young people aged 8-25 years: a systematic review and meta-analysis
Source: BMC Public Health. 2022 Jun 3;22:1111. doi: 10.1186/s12889-022-13072-5 (PMC9165543; doi:10.1186/s12889-022-13072-5)
Supplement: Supplementary file 3 — Additional file 3. Excluded studies from meta-analysis. [file 12889_2022_13072_MOESM3_ESM.docx]

**Additional File 3: Details of data transformations and preparation for analysis**

Conversion of outcome data to log odds ratio and standard error

To maximise the number of studies we could include in the meta-analyses we converted all outcome data to log odds ratios and standard errors, using the methods described in Borenstein 2009 (see Chapter 7) [1]. To enable this, first the means and standard deviations were used to calculate a pooled standard deviation, and then a standardised mean difference, using the Calculator Companion to Practical Meta-analysis [2]. The standardized mean-difference can be computed from means and standard deviations, a t-test, and a one-way ANOVA. Standardised mean difference (SMD) was computed using means and standard errors (SE), where standard deviations were not reported. Where possible and logical, categorical groups were combined to dichotomise. Standard errors for SMDs, ORs or log odds ratios (logORs) were calculated using 95% confidence intervals or p-values where not directly reported.

We note that this is a change from protocol, where we described converting data to a standardised mean difference. In practice, using the log odds ratio required fewer transformations to be conducted and so was preferred.

Cluster randomised trials, intra-cluster correlations and standard error

Some RCTs were cluster RCTs, with the unit of randomisation being a group or ‘cluster’, for example a school. As participants in any given cluster are expected to respond to interventions in a similar way, their data are not independent and therefore the clustering effect must be accounted for in analyses to avoid unit-of-analyses error [3]. Where studies used a clustered design but ignored clustering in their analyses, or where it was not clear, we inflated the SE to account for intra-cluster correlations. Inflated SEs were calculated by multiplying the SE of the effect estimate (logOR) by the square root of the design effect. Design effect = 1 + (M – 1) ICC, where M is the average cluster size in the trial and ICC is the intracluster correlation coefficient. ICCs provide a measure of the relative variability within and between clusters, and where not reported, were derived from other included studies that reported an ICC for the same outcome, to enable the design effect to be calculated. For all analyses, we selected the mean ICC for the outcome. When no ICC was available for that outcome, we used the mean ICC for the substance behaviour as an estimate (see Table below).

**
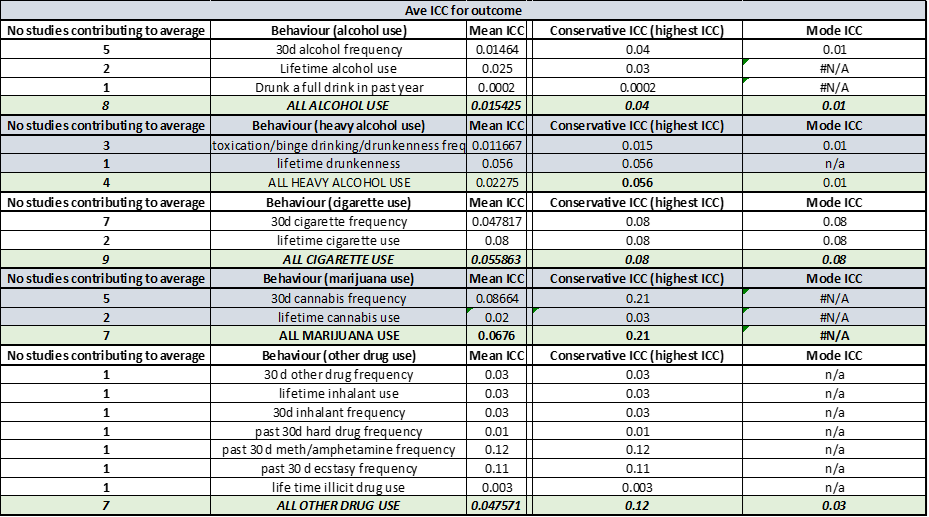
**

**Additional Table 3.1: Average intracluster correlation coefficient for each outcome**

Combining intervention groups

To combine group means and group standard deviations, the following two formulas were used respectively (I think I got these from the Cochrane handbook?): ((N1 X M1) + (N2 X M2)) / (N1 + N2); and SQRT((((N1-1) X (SD1^2))+((N2-1) X (SD2^2))+(((N1 X N2)/(N1+N2)) X ((M1^2)+(M2^2)-(2 X M1 X M2))))/(N1+N2-1)); where N1 and N2 are sample sizes; M1 and M2 are means; and SD1 and SD2 and standard deviations of the two groups.

Where proportions were provided, groups were combined by reconstructing the 3x2 table and combining the numbers in the intervention groups.  For 3-arm studies where only log(OR) and selog(OR) were provided, we had to assumed a control group risk to be able to reconstruct the 3x2 table of events/non-events for each group and combine intervention groups. The studies in question were from a suite of similar studies, in which the same intervention was trialled by the same authors, in similar populations, but in different cities/schools, some of which provided data on control group risk to allow estimation. Using the odds ratios (OR) and assumed control group risk (ACR), the corresponding intervention risk was obtained as (reference section 14.1.5.1 of Cochrane handbook): Corresponding intervention risk per 1000 = 1000 × ((OR × ACR)/(( 1 – ACR) + (OR × ACR))). The combined 2x2 table was used to calculate log(OR) and selog(OR) in the usual way, and cluster-adjusted if required.

One study (White, 2017) had 3 intervention arms: two different types of intervention individually and an arm where both interventions were given. We chose to use the combined intervention arm and exclude the two individual intervention arms.

One study (Ellickson, 2003) administered the intervention and reported separately by sex an adjusted risk. This was used to calculate expected number of events and to combine both sexes into one lager intervention group.

1. Borenstein, M., et al., *Effect sizes for continuous data.* The handbook of research synthesis and meta-analysis, 2009. **2**: p. 221-235

2. Wilson, D. *Practical Meta-Analysis Effect Size Calculator*. January 2021]; Available from: <https://www.campbellcollaboration.org/escalc/html/EffectSizeCalculator-SMD-main.php>.

3. Higgins, J.P. and S. Green. *Cochrane Handbook for Systematic Reviews of Interventions Version 5.1.0*. 2011 [cited 4 23/01/2018]; Available from: [www.handbook.cochrane.org](file:///Users/lauratinner/Downloads/www.handbook.cochrane.org).
