# Supplementary material for: Individual-, family- and school-based interventions to prevent multiple risk behaviours relating to alcohol, tobacco and drug use in young people aged 8-25 years: a systematic review and meta-analysis
Source: BMC Public Health. 2022 Jun 3;22:1111. doi: 10.1186/s12889-022-13072-5 (PMC9165543; doi:10.1186/s12889-022-13072-5)
Supplement: Supplementary file 4 — Additional file 4. Details of data transformations. [file 12889_2022_13072_MOESM4_ESM.docx]

| **Author and Year** | **Trial name** | **Reason** |
| --- | --- | --- |
| Botvin 2015 | Botvin 12 | Polysubstance use |
| Byrnes 2012 | Family matters vs SFP | No substance use |
| Evers 2012 | Your decisions count | No substance use |
| Huang 2013 | Life Skills + TPD Taiwan - Huang 2013 | No substance use |
| Marsiglia 2015 | Keepin it Real (Mexico) | No substance use |
| Moskowitz 1984 | Drug education program 1980 (The Napa Project) | No substance |
| Schaps 1982 | Drug education program | Polysubstance use |
| Seal 2006 | LST - Seal 2006 | Polysubstance use |
| Skeer 2016 | SUPPER | No substance use |
| Vogl 2014 | Climate schools | No substance use |

**Additional File 4: Studies excluded from synthesis and meta-analyses**

Additional Table 4.1: Excluded studies from synthesis

Legend: These studies were excluded from the synthesis because they did not report results for our outcomes of interest. Decisions were made *a priori* and reasons given below:

*No substance use outcome* - no results for outcomes relating to substance use were reported.

*Polysubstance use outcome* - results were in relation to multiple substances combined (alcohol, tobacco and/or drugs) rather than each substance type individually, which was not an outcome of interest for this review.

**Additional Table 4.2: Excluded studies from meta-analyses**

| **Author and Year** | **Trial name** | **Reason** |
| --- | --- | --- |
| Bell 1993 | ALERT Bell | Subgroup analysis only |
| Botvin 1990 | Botvin 10 | Full set of parameters not provided |
| Botvin 1990a | Botvin 56 | Full set of parameters not provided |
| Botvin 2001 | Botvin 29 | Assessment time period unclear |
| Calafat 1989 | Programa Tú decides (Decide yourself program) | Full set of parameters not provided |
| Clark 2010 | Success | Full set of parameters not provided |
| Clayton 1996 | DARE | Growth model coefficients |
| de la Rosa 1995 | Decide yourself program (Programa Tú decides) | Full set of parameters not provided |
| Dent 2001 | Towards no drug abuse E | Full set of parameters not provided |
| Donaldson 1994 | AAPT donaldson | Full set of parameters not provided |
| Eisen 2002 | Lions quest | Initiation (subgroup analysis only) |
| Gilchrist 1987 | Skills enhancement programme | Mean change from baseline |
| Hall 2013 | Too Good for Drugs | Subgroup analysis only |
| Hallfors 2006 | Reconnecting youth | Full set of parameters not provided |
| Hecht 2003 | Keepin it Real | Growth model coefficients |
| Hecht 2008 | Keepin' it Real (Mexico) | Growth model coefficients |
| Horan 1982 | Assertion training | Mean change from baseline |
| Komro 2008 | Project Northland | Growth model coefficients |
| Newton 2009 | Climate schools | Mean change from baseline |
| Okamoto 2016 | Ho'ouna Pono | Full set of parameters not provided |
| Rohrbach 1994 | Midwestern prevention project (Project STAR) | Subgroup analysis only |
| Smith 2004 | LST - Smith | Subgroup analysis only and full set of parameters not provided |

Legend: These studies were excluded from our meta-analyses due to there being insufficient data available for substance use outcome(s). Study authors were contacted if there was missing or unclear data in the first instance. Decisions to not include these types were made *a priori*, and rationale is given below:

*Subgroup analysis only* – where randomisation had been conducted on the whole cohort, yet results for only a subgroup reported, or a subgroup had been censored, randomisation will have been lost leading to high risk of bias in results.

*Initiation* – a type of subgroup analyses, as results only relate to non-users at baseline.

*Mean change from baseline* – results relating to ‘differences in change over time’ are not combinable with endpoint differences in meta-analysis. There were not enough studies reporting ‘differences in change over time’ for any outcomes to be able to perform a separate meta-analyses.

*Growth model coefficients* – are a type of ‘difference in change over time’ measure, so same rationale applies as for ‘mean change from baseline’.

*Full set of parameters not* *provided* – to be able to include study results in meta-analysis, a set of parameters need to be provided, or sufficient data for these parameters to be calculated provided, otherwise it was not possible to include in meta-analyses.
