# Supplementary material for: Individual-, family- and school-based interventions to prevent multiple risk behaviours relating to alcohol, tobacco and drug use in young people aged 8-25 years: a systematic review and meta-analysis
Source: BMC Public Health. 2022 Jun 3;22:1111. doi: 10.1186/s12889-022-13072-5 (PMC9165543; doi:10.1186/s12889-022-13072-5)
Supplement: Supplementary file 5 — Additional file 5. Outcome measures. [file 12889_2022_13072_MOESM5_ESM.docx]

**Additional File 5: Selection of outcome measures**

Where multiple options of data were presented for a substance-use outcome, preference for which data to use was ordered as follows:

1. Raw mean (SD or SE) + ICC
2. Cluster adjusted mean (SE) + ICC
3. Unadjusted OR (SE) + ICC
4. Cluster adjusted OR (SE) + ICC
5. Cluster and covariate adjusted mean (SE) + ICC
6. Cluster and covariate adjusted OR (SE) + ICC

All values used were end-point values to enable comparison of end-point outcomes between intervention groups and control groups. Where only change from baseline were presented (such as growth model coefficients) we did not include in the meta-analysis. This is because change over time values cannot be combined with end point values in meta-analyses.

This hierarchy could only be used if all the information required for meta-analyses were provided. For example, in one study (Sanchez 2017) adjusted odds ratios and 95% confidence intervals used to calculate standard error, rather than unadjusted proportions, since the number of participants in each group was unclear.

The units used for each measure in each study are shown in Tables 5.1-5.6 below.

**Additional Tables 5.1 – 5.6: VARIATION IN OUTCOMES ACROSS STUDIES**

**Additional Table 5.1 Alcohol**

(Follow-up: short-term and long term*)

| **Author** | **Study** | **F-up (from intervention end)** | **Measure** | **Units** | **Type of intervention** |
| --- | --- | --- | --- | --- | --- |
| Faggiano 2008 | EU_DAP | 15 months* | Alcohol use in past week | Binary proportion | U-S |
| Bauman 2002 | Family matters | 12 months | Ever used alcohol | Binary proportion | U-F |
| Snow 1992 | Adolescent Decision Making Programme | Immediate | Alcohol use in past year | Categorical, combined into binary proportion | U-S |
| Elder 2002 | Migrant education | 12 months | Alcohol use in past month | Binary proportion | T-S&F |
| Elder 2002 | Migrant education | 2 years* | Alcohol use in past month | Binary proportion | T-S&F |
| Fang 2010 | Mother-Daughter - Asian-American | 6 months | Number of occasions of alcohol use in past month | Mean, sd | T-F |
| Fang 2010 | Mother-Daughter - Asian-American | 2 year* | Number of occasions of alcohol use in past month | Mean, sd | T-F |
| Schinke 2009 | Mother-Daughter - Non-Specific Population | 1 year | Number of occasions of alcohol use in past month | Mean, sd | U-F |
| Schinke 2009 | Mother-Daughter - Non-Specific Population | 2 year* | Number of occasions of alcohol use in past month | Mean, sd | U-F |
| McCambridge 2005 | Motivational interviewing 2005 | 12 months | Mean alcohol units per week | Mean, 95%CI | T-S |
| McCambridge 2008 | Motivational interviewing 2008 | 6 months | Number of occasions of alcohol use in past month | Mean, sd | T-S |
| McCambridge 2011 | Motivational interviewing 2011 | 12 months | Alcohol use in past month | CluAdjOR, 95%CI | U-S |
| Schwinn 2010 | RealTeen | 6 months | Number of occasions of alcohol use in past month | Adj mean, se | U-I |
| Ringwalt 2009 | ALERT Ringwalt | 1 year | Alcohol use in past month | Binary proportion (assumed equal groups) | U-S |
| St Pierre 2005 | ALERT St Pierre | Approximately 2 years* | Alcohol use in past month | Binary proportion, assumed equal groups | U-S |
| Ellickson 2003 | ALERT + | 1 year | Alcohol use in past week | Adj (expected) binary proportion | U-S |
| Sussman 1998 | Towards no drug abuse A | 1 year | Number of occasions of alcohol use in past month | Mean, sd | T-S |
| Sussman 1998 | Towards no drug abuse A | 4 or 5 years* | Number of occasions of alcohol use in past month | Mean, sd | T-S |
| Sussman 2003 | Towards no drug abuse B | 2 years* | Alcohol use in past month | AdjOR, se estimated by recreating 2x2 table and assuming control group risk | T-S |
| Valente 2007 | Towards no drug abuse C | 1 year | Alcohol use in past month | MLM regression coefficient, se estimated by recreating 2x2 table and assuming control group risk | T-S |
| Sun 2008 | Towards no drug abuse D | 1 year | Alcohol use in past month | Adj OR, 95%CI | U-S |
| Sloboda 2009 | ASAP | 2 years* | Alcohol use in past month | Binary proportion | U-S |
| Roberts 2011 | Aussie optimism | 1 year | Alcohol use in past month | Binary proportion | U-S&F |
| Botvin 1995 | Botvin 6 | 2 years* | Current alcohol users - once a month or more | Binary proportion | U-S |
| D'Amico 2012 | CHOICE | 6 months | Alcohol use in past month | Log(OR), se(LogOR) | U-S |
| Midford 2014 | DEVS | 1 year | Alcohol use in past year | Binary proportion | U-S |
| Malmberg 2014 | Healthy school and drugs | 6 months | Alcohol use in past month | Categorical, combined into binary proportion | U-S |
| Sussman 2012 | Project Towards no Drug Abuse | 12 months | Alcohol use in past month | Binary proportion | U-S |
| Schwinn 2018 | RealTeen | 12 months | Number of occasions of alcohol use in past month | Adj mean, se | U-I |
| Sanchez 2017 | #Tamojunto Drug Prevention Program | 6 months | Alcohol use in past month | Adj OR, 95%CI | U-S |
| Sanchez 2017 | #Tamojunto Drug Prevention Program | 18 months* | Alcohol use in past month | Adj OR, 95%CI | U-S |
| White 2017 | Assist+FRANK | 4 months | Ever used alcohol | Binary proportion | U-S |
| Guo 2015 | Life Skills + TPD Taiwan - Guo 2015 | 12-13 months | Alcohol use in past month | Binary proportion | U-S |
| Weichold 2012 | IPSY | Immediately | Number of occasions of alcohol use in past month | Mean, sd | U-S |
| Weichold 2012 | IPSY | 2 years 10 months* | Number of occasions of alcohol use in past month | Mean, sd | U-S |
| Hernandez-Serrano 2013 | Saluda | 6 months | Alcohol use in past month | Binary proportion | U-S |
| Butzer 2017 | Yoga | 12 months | Ever used alcohol | Binary proportion | U-S |
| D'Amico 2013 | Free Talk | 3 months | Number of occasions of alcohol use in past month | SMD, p | T-I |

*Indicating long term follow-up period

**Additional Table 5.2 Heavy/Binge drinking**

(Follow-up: short-term and long term*)

| **Author** | **Study** | **F-up (from intervention end)** | **Measure** | **Units** | **Type of intervention** |
| --- | --- | --- | --- | --- | --- |
| Faggiano 2008 | EU_DAP | 15 months* | 3 or more episodes of drunkenness in past month | Binary proportion | U-S |
| Faggiano 2008 | EU_DAP | 3 months | 3 or more episodes of drunkenness in past month | Binary proportion | U-S |
| D'Amico 2012 | CHOICE | 6 months | Heavy drinking in past month | Log(OR), se(LogOR) | U-S |
| Malmberg 2014 | Healthy school and drugs | 6 months | Binge drinking in past month | Categorical, combined into binary proportion | U-S |
| Sussman 2012 | Project Towards no Drug Abuse | 12 months | Being drunk in past month | Binary proportion | U-S |
| Schwinn 2018 | RealTeen | 12 months | Binge drinking in past month | Adj mean, se | U-I |
| Sanchez 2017 | #Tamojunto Drug Prevention Program | 6 months | Binge drinking in past month | Adj OR, 95%CI | U-S |
| Sanchez 2017 | #Tamojunto Drug Prevention Program | 18 months* | Binge drinking in past month | Adj OR, 95%CI | U-S |
| Hernandez-Serrano 2013 | Saluda | 6 months | Drunkenness in past month | Binary proportion | U-S |
| D'Amico 2013 | Free Talk | 3 months | Heavy drinking in past month | SMD, p | T-I |
| Skarstrand 2013 | Swedish SFP | 10 months | Drunkenness in past month | Adj OR, 95%CI | U-C(S&F) |
| Skarstrand 2013 | Swedish SFP | 34 months | Drunkenness in past month | Adj OR, 95%CI | U-C(S&F) |

**Additional Table 5.3 Tobacco**

(Follow-up: short-term and long term)

| **Author** | **Study** | **F-up (from intervention end)** | **Measure** | **Units** | **Type of intervention** |
| --- | --- | --- | --- | --- | --- |
| Faggiano 2008 | EU_DAP | 15 months* | Cigarette use in past month | Binary proportion | U-S |
| Bauman 2002 | Family matters | 12 months | Ever used cigarettes | Binary proportion | U-F |
| Snow 1992 | Adolescent Decision Making Programme | Immediate | Used tobacco in past year | Binary proportion | U-S |
| Elder 2002 | Migrant education | 12 months | Cigarette use in past month | Binary proportion | T-S&F |
| Elder 2002 | Migrant education | 2 years* | Cigarette use in past month | Binary proportion | T-S&F |
| Fang 2010 | Mother-Daughter - Asian-American | 6 months | Number of occasions of cigarette use in past month | Mean, sd | T-F |
| Fang 2010 | Mother-Daughter - Asian-American | 2 year* | Number of occasions of cigarette use in past month | Mean, sd | T-F |
| McCambridge 2005 | Motivational interviewing 2005 | 12 months | Mean cigarettes per week in past month | CluAdj Mean, 95%CI | T-S |
| McCambridge 2008 | Motivational interviewing 2008 | 6 months | Mean number of cigarettes per day in past month | Mean, sd | T-S |
| McCambridge 2011 | Motivational interviewing 2011 | 12 months | Cigarette use in past month | CluAdj OR, 95%CI | U-S |
| Schwinn 2010 | RealTeen | 6 months | Number of occasions of cigarette use in the past month | Adj mean, se | U-I |
| Ringwalt 2009 | ALERT Ringwalt | 1 year | Cigarette use in past month | Binary proportion (assumed equal groups) | U-S |
| St Pierre 2005 | ALERT St Pierre | Approximately 2 years* | Cigarette use in past month | Binary proportion (assumed equal groups) | U-S |
| Ellickson 2003 | ALERT + | 1 year | Tobacco use in past week | Adj (expected) binary proportion | U-S |
| Sussman 1998 | Towards no drug abuse A | 1 year | Number of occasions of cigarette use in past month | Mean, sd | T-S |
| Sussman 1998 | Towards no drug abuse A | 4 or 5 years* | Number of occasions of cigarette use in past month | Mean, sd | T-S |
| Sussman 2003 | Towards no drug abuse B | 2 years* | Cigarette use in past month | Adj OR, se estimated by recreating 2x2 table and assuming control group risk | T-S |
| Valente 2007 | Towards no drug abuse C | 1 year | Monthly tobacco use score | MLM regression coefficient, se estimated by recreating 2x2 table and assuming control group risk | T-S |
| Sun 2008 | Towards no drug abuse D | 1 year | Tobacco use in past month | Adj OR, 95%CI | U-S |
| Sloboda 2009 | ASAP | 2 years* | Cigarette use in past month | Binary proportion | U-S |
| Roberts 2011 | Aussie optimism | 1 year | Cigarette use in past month | Binary proportion | U-S&F |
| Midford 2014 | DEVS | 1 year | Cigarette use in past year | Binary proportion | U-S |
| Malmberg 2014 | Healthy school and drugs | 6 months | Cigarette use in past month | Categorical, combined into binary proportion | U-S |
| Sussman 2012 | Project Towards no Drug Abuse | 12 months | Cigarette use in past month | Binary proportion | U-S |
| Schwinn 2018 | RealTeen | 12 months | Number of occasions of cigarette use in past month | Marginal (adj) mean, se | U-I |
| Sanchez 2017 | #Tamojunto Drug Prevention Program | 6 months post intervention | Tobacco use in past month | Adj OR, 95%CI | U-S |
| Sanchez 2017 | #Tamojunto Drug Prevention Program | 18 months post intervention* | Tobacco use in past month | Adj OR, 95%CI | U-S |
| White 2017 | Assist+FRANK | 4 months | Ever tried smoking | Binary proportion | U-S |
| Guo 2015 | Life Skills + TPD Taiwan - Guo 2015 | 12-13 months | Tobacco use in past month | Binary proportion | U-S |
| Weichold 2012 | IPSY | post intervention | Number of occasions of cigarette use in past month | Mean, sd | U-S |
| Weichold 2012 | IPSY | 2 years 10 months* | Number of occasions of cigarette use in past month | Mean, sd | U-S |
| Butzer 2017 | Yoga | 1 year | Ever tried a cigarette | Binary proportion | U-S |
| Skarstrand 2013 | Swedish SFP | 10 months | Ever used cigarettes | Adj OR, 95%CI | U-C(S&F) |
| Skarstrand 2013 | Swedish SFP | 34 months* | Ever used cigarettes | Adj OR, 95%CI | U-C(S&F) |

*Indicating long term follow up period

**Additional Table 5.4 Heavy Tobacco**

(Follow-up: short-term and long term*)

| **Author** | **Study** | **F-up (from intervention end)** | **Measure** | **Units** | **Type of intervention** |
| --- | --- | --- | --- | --- | --- |
| Faggiano 2008 | EU_DAP | 15 months* | Prevalence of daily cigarette use in past month | Binary proportion | U-S |
| White 2017 | Assist+FRANK | 4 months | Prevalence of weekly cigarette use in past month | Binary proportion | U-S |

*****Indicating long term follow up period

**Additional Table 5.5 Cannabis**

(Follow-up: short-term and long term*)

| **Author** | **Study** | **F-up (from intervention end)** | **Measure** | **Units** | **Type of intervention** |
| --- | --- | --- | --- | --- | --- |
| Snow 1992 | Adolescent Decision Making Programme | Immediate | Cannabis use in past year | Binary proportion | U-S |
| Fang 2010 | Mother-Daughter - Asian-American | 6 months | Number of occasions of cannabis use in past month | Mean, sd | T-F |
| Fang 2010 | Mother-Daughter - Asian-American | 2 years* | Number of occasions of cannabis use in past month | Mean, sd | T-F |
| McCambridge 2005 | Motivational interviewing 2005 | 12 months | Mean frequency of cannabis use (number of joints) in past week | Mean, 95%CI | T-S |
| McCambridge 2008 | Motivational interviewing 2008 | 6 months | Mean frequency of cannabis use (number of joints) in past week | Mean, sd | T-S |
| McCambridge 2011 | Motivational interviewing 2011 | 12 months | Prevalence of cannabis use | cluAdj OR | U-S |
| Schwinn 2010 | RealTeen | 6 months | Number of occasions of cannabis use in past month | Adj Mean, se | U-I |
| Ringwalt 2009 | ALERT Ringwalt | 1 year | Number of occasions of cannabis use in past month | Binary proportion (assumed equal groups) | U-S |
| St Pierre 2005 | ALERT St Pierre | Approximately 2 years* | Number of occasions of cannabis use in past month | Binary proportion (assumed equal groups) | U-S |
| Ellickson 2003 | ALERT + | 1 year | Cannabis use in past week | Adj (expected) binary proportion | U-S |
| Sussman 1998 | Towards no drug abuse A | 1 year | Number of occasions of cannabis use in past month | Mean, sd | T-S |
| Sussman 1998 | Towards no drug abuse A | 4 or 5 years* | Number of occasions of cannabis use in past month | Mean, sd | T-S |
| Sussman 2003 | Towards no drug abuse B | 2 years* | Number of occasions of cannabis use in past month | AdjOR, se estimated by recreating 2x2 table and assuming control group risk | T-S |
| Valente 2007 | Towards no drug abuse C | 1 year | Cannabis use in past month | MLM regression coefficient, se estimated by recreating 2x2 table and assuming control group risk | T-S |
| Sun 2008 | Towards no drug abuse D | 1 year | Cannabis use in past month | Adj OR, 95%CI | U-S |
| Sloboda 2009 | ASAP | 2 years* | Cannabis use in past month | Binary proportion | U-S |
| Botvin 1995 | LST - Botvin 6 | 2 years* | Current cannabis experimenters | Binary proportion | U-S |
| Malmberg 2014 | Healthy school and drugs | 6 months | Ever used cannabis | Binary proportion | U-S |
| Sussman 2012 | Project Towards no Drug Abuse | 1 year | Cannabis use in past month | Binary proportion | U-S |
| Schwinn 2018 | RealTeen | 12 months | Number of occasions of cannabis use in past month | Marginal (adj) mean, se | U-I |
| Sanchez 2017 | #Tamojunto Drug Prevention Program | 6 months | Cannabis use in past month | Adj OR, 95%CI | U-S |
| Sanchez 2017 | #Tamojunto Drug Prevention Program | 18 months* | Cannabis use in past month | Adj OR, 95%CI | U-S |
| White 2017 | Assist+FRANK | 4 months | Cannabis use in past month | Binary proportion | U-S |
| Butzer 2017 | Yoga | 1 year | Ever tried cannabis | Binary proportion | U-S |
| D'Amico 2013 | Free Talk | 3 months | Number of occasions of cannabis use in past month | SMD, p | T-I |

*****Indicating long term follow up period

**Additional Table 5.6 Other illicit drugs**

(Follow-up: short-term and long term*)

| **Author** | **Study** | **F-up (from intervention end)** | **Measure** | **Units** | **Type of intervention** |
| --- | --- | --- | --- | --- | --- |
| Fang 2010 | Mother-Daughter - Asian-American | 6 months | Number of occasions of prescription drug use in past month | Mean, sd | T-F |
| Fang 2010 | Mother-Daughter - Asian-American | 2 year* | Number of occasions of prescription drug use in past month | Mean, sd | T-F |
| Schwinn 2010 | RealTeen | 6 months | Number of occasions of poly drug use in past month | Adj mean, se | U-I |
| Ringwalt 2009 | ALERT Ringwalt | 1 year | Number of occasions of inhalant use in past month | Binary proportion (assumed equal groups) | U-S |
| Sussman 1998 | Towards no drug abuse A | 1 year | Number of occasions of hard drug use in past month | Mean, sd | T-S |
| Sussman 1998 | Towards no drug abuse A | 4 or 5 years* | Number of occasions of hard drug use in past month | Mean, sd | T-S |
| Sussman 2003 | Towards no drug abuse B | 2 years* | Hard drug use in past month | AdjOR, se estimated by recreating 2x2 table and assuming control group risk | T-S |
| Valente 2007 | Towards no drug abuse C | 1 year | Cocaine use in past month | MLM regression coefficient, se estimated by recreating 2x2 table and assuming control group risk | T-S |
| Sun 2008 | Towards no drug abuse D | 1 year | Hard drug use in past month | Adj OR, 95%CI | U-S |
| Vogl 2014 | Climate schools | 10 months | Ever used meth/amphetamine | Binary proportion | U-S |
| Vogl 2014 | Climate schools | 10 months | Ever used ecstasy | Binary proportion | U-S |
| Sussman 2012 | Project Towards no Drug Abuse | 1 year | Hard drug use in past month | Binary proportion | U-S |
| Schwinn 2018 | RealTeen | 12 months | Number of occasions of other drug use in past month | Adj mean, se | U-I |
| Sanchez 2017 | #Tamojunto Drug Prevention Program | 6 months post intervention | Inhalant use in past month | Adj OR, 95%CI | U-S |
| Sanchez 2017 | #Tamojunto Drug Prevention Program | 18 months post intervention* | Inhalant use in past month | Adj OR, 95%CI | U-S |
| Sanchez 2017 | #Tamojunto Drug Prevention Program | 6 months post intervention | Cocaine use in past month | Adj OR, 95%CI | U-S |
| White 2017 | Assist+FRANK | 4 months | Ever used illicit drugs | Binary proportion | U-S |
| Guo 2015 | Life Skills + TPD Taiwan | 12-13 months | Illicit drug use (including cannabis) in past month | Binary proportion | U-S |
| Butzer 2017 | Yoga | 1 year | Ever tried cocaine | Binary proportion (with continuity correction of 0.5 added to each cell of 2x2 table) | U-S |
| Butzer 2017 | Yoga | 1 year | Ever sniffed glue | Binary proportion | U-S |
| Skarstrand 2013 | Swedish SFP | 10 months | Ever used illicit drugs (including cannabis) | Adj OR, 95%CI | U-C(S&F) |
| Skarstrand 2013 | Swedish SFP | 34 months* | Ever used illicit drugs (including cannabis) | Adj OR, 95%CI | U-C(S&F) |

*Indicating long term follow up period
