# Supplementary material for: Individual-, family- and school-based interventions to prevent multiple risk behaviours relating to alcohol, tobacco and drug use in young people aged 8-25 years: a systematic review and meta-analysis
Source: BMC Public Health. 2022 Jun 3;22:1111. doi: 10.1186/s12889-022-13072-5 (PMC9165543; doi:10.1186/s12889-022-13072-5)
Supplement: Supplementary file 6 — Additional file 6. Planned subgroup analysis. [file 12889_2022_13072_MOESM6_ESM.docx]

**Additional File 6: List of planned subgroup analyses**

The following analyses to compare subgroups by parameters were planned in the protocol to investigate heterogeneity. Data were either insufficiently reported or there were not enough studies to allow us to complete most of these analyses. We were able to conduct part of subgroup analysis f. as we analyzed the main results by type of intervention (family, school, individual, combination and universal or targeted).

1. Age group at start of intervention
2. Gender
3. Participants (individual, infant, child, adolescent, parent, guardian, carer, grandparent, teacher, nurse)
4. Number of behaviours targeted
5. Duration of intervention
6. Type of intervention (pre- or antenatal, family, preschool, school, friendship group; and whether the intervention was universal or targeted to a high-risk group(s))
7. High-income or low- and middle-income country
