# Supplementary material for: Individual-, family- and school-based interventions to prevent multiple risk behaviours relating to alcohol, tobacco and drug use in young people aged 8-25 years: a systematic review and meta-analysis
Source: BMC Public Health. 2022 Jun 3;22:1111. doi: 10.1186/s12889-022-13072-5 (PMC9165543; doi:10.1186/s12889-022-13072-5)
Supplement: Supplementary file 7 — Additional file 7. Table of characteristics. [file 12889_2022_13072_MOESM7_ESM.docx]

## Additional File 7: Study characteristics and risk of bias

## Table 7.1: Descriptive characteristics of included studies

| **Primary Authors**  ***included in meta-analysis** | **Study name** | **Study Type** | **Country** | **Duration** | **Theory** | **Follow-up (post-intervention)** | **Components** | **Age at start of intervention (as reported)** | **Behaviours targeted** | **Process evaluation** | **Risk of bias ^a^** | | | | | | |
| --- | --- | --- | --- | --- | --- | --- | --- | --- | --- | --- | --- | --- | --- | --- | --- | --- | --- |
| **School Universal studies** | | | | | | | | | | | **1** | **2** | **3** | **4** | **5** | **6** | **7** |
| Bell 1993 [1] | ALERT Bell | School universal | USA | 12-24 months | Health belief model to build skills to resist drugs. Bandura's work on the self-efficacy theory of behavioural change (Social influence model). | 5 years | Role play, psychodrama, written work to practise saying no | Seventh grade | Cigarettes, alcohol and marijuana | Yes, quantitative and qualitative methods. Acceptability, reach and fidelity data. |  |  |  |  |  |  |  |
|  |  |  |  |  |  |  |  |  |  |  |  | | | | | | |
| Botvin 1990 [2] | Botvin 10 | School universal | USA | 12-24 months | N/S | 12 months | Curriculum - 20 session prevention prog in 7th grade, 10 session peer-led booster in 8th grade. | Eighth grade | Tobacco, alcohol, drugs | No |  |  |  |  |  |  |  |
|  |  |  |  |  |  |  |  |  |  |  |  | | | | | | |
| Botvin 1990a [3] | Botvin 56 | School universal | USA | 24+ months | N/S | 13 years | Curriculum - 15 lessons, 10 boosters in 8th grade, 5 boosters in 9th grade | Seventh grade | Tobacco, alcohol, drugs | Yes, quantitative methods only. Fidelity and reach data. |  |  |  |  |  |  |  |
|  |  |  |  |  |  |  |  |  |  |  |  | | | | | | |
| Botvin 1995*[4] | Botvin 6 | School universal with targeted component | USA | 12-24 months | N/S | 2 years | Demonstration, behavioural feedback, reinforcement, curriculum with stories, video, peer leaders, behavioural homework assignments | Mean: 12.7 years | Alcohol and drugs | No |  |  |  |  |  |  |  |
|  |  |  |  |  |  |  |  |  |  |  |  | | | | | | |
| Botvin 2001 [5] | Botvin 29 | School universal | USA | 12-24 months | N/S | 12 months | Group discussion, demonstration,  modelling, behavioural rehearsal, feedback and  reinforcement, and behavioural “homework” assignments  for out-of-class practice | Mean: 12.9 | Tobacco, alcohol, drugs | No |  |  |  |  |  |  |  |
|  |  |  |  |  |  |  |  |  |  |  |  | | | | | | |
| Botvin 2015 [6] | Botvin 12 | School universal | USA | 3-6 months | N/S | 12 months | 10-session curriculum | 71.5% 9th grade, remainder 10th grade.   Mean: 15.2 years | Tobacco, alcohol, drugs | No |  |  |  |  |  |  |  |
|  |  |  |  |  |  |  |  |  |  |  |  | | | | | | |
| Butzer 2017* [7] | Yoga | School universal | USA | 3-6 months | N/S | 12 months | Physical activity, mindfulness, didactic teaching, group discussion | Mean: 12.64 (SD = 0.33) | Substance use (ATOD) | Yes, quantitative and qualitative. Acceptability and reach data. |  |  |  |  |  |  |  |
|  |  |  |  |  |  |  |  |  |  |  |  | | | | | | |
| Calafat 1989 [8] | Programa Tú decides (Decide yourself program) | School universal | Spain | Unclear | N/S | 7 months | Teacher training and curriculum, a comic, a list of questions, hypothetical situations to make decisions and information on drugs. Teachers met with parents to explain the contents of the program and the risk factor of drugs use | N/S | Alcohol, tobacco and cannabis use | No |  |  |  |  |  |  |  |
|  |  |  |  |  |  |  |  |  |  |  |  | | | | | | |
| Clayton 1996 [9] | DARE | School universal | USA | 3-6 months | N/S | 5 years | Didactic and interactive approaches, with role playing and homework | 90% aged 11 or 12 years | Tobacco, alcohol and marijuana | Yes, quantitative methods only. Reach data. |  |  |  |  |  |  |  |
|  |  |  |  |  |  |  |  |  |  |  |  | | | | | | |
| D'Amico 2012* [10] | CHOICE | School universal | USA | <3 months | Social Learning Theory, Decision-Making Theory, Self-Efficacy Theory and a motivational interviewing (MI) approach | 8 months | Curriculum, group discussion, role play | Sixth – eighth grade | Alcohol and substances (particularly marijuana) use | Yes, quantitative methods only. Acceptability, reach and fidelity data. |  |  |  |  |  |  |  |
|  |  |  |  |  |  |  |  |  |  |  |  | | | | | | |
| de la Rosa 1995 [11] | Decide yourself program (Programa Tú decides) | School universal | Spain | <3 months | N/S | 2 years | Curriculum booklet | Aged 13 to 23 years old | Illegal and legal drug use | No |  |  |  |  |  |  |  |
|  |  |  |  |  |  |  |  |  |  |  |  | | | | | | |
| Dent 2001 [12] | Towards no drug abuse E | School universal | USA | <3 months | Motivation-skills-decision making model | 12 months | Curriculum, didactic information and listening | Aged 14 to 17 years | Tobacco, alcohol, marijuana and other drugs | Yes, unclear methods. Reach data. |  |  |  |  |  |  |  |
|  |  |  |  |  |  |  |  |  |  |  |  | | | | | | |
| Donaldson 1994 [13] | AAPT donaldson | School universal | USA | Unclear | Social influence and social inoculation theory | 12 months | Curriculum | N/S | Alcohol, tobacco and marijuana | No |  |  |  |  |  |  |  |
|  |  |  |  |  |  |  |  |  |  |  |  | | | | | | |
| Eisen 2002 [14] | Lions quest | School universal | USA | 6-12 months | Social influence and social cognitive approaches | 12 months | Curriculum and student workbooks | <11 (0.5%) 11 (51.1%) 12 (45%) 13 (2.9%) 14 (0.2%) Missing (0.3%) | Alcohol, tobacco, marijuana, cocaine use and other illegal substances | No |  |  |  |  |  |  |  |
|  |  |  |  |  |  |  |  |  |  |  |  | | | | | | |
| Ellickson* 2003 [15] | ALERT + | School universal | USA | 24+ months | Social influence model of prevention. Made up of health belief model, social learning model and self-efficacy theory of behaviour. | 3 years | Curriculum, interactive teaching methods and home-learning activities (parental involvement). ALERT+ (Intervention 2): Parent/child involvement in planning drug-free parties and didactic teaching | Seventh grade | Cigarettes, alcohol and marijuana | Yes, quantitative methods only. Acceptability, reach and fidelity data. |  |  |  |  |  |  |  |
|  |  |  |  |  |  |  |  |  |  |  |  | | | | | | |
| Evers 2012 [16] | Your decisions count | School universal | USA | <3 months | Transtheoretical stages of change model | 14 months | Computer-based assessment and feedback on current behaviour/progress, personalised advice on how to further progress in relation to changing drug-associated behaviour, movie testimonials from adolescents | N/S | Alcohol, tobacco, marijuana, and other drug use | No |  |  |  |  |  |  |  |
|  |  |  |  |  |  |  |  |  |  |  |  | | | | | | |
| Faggiano* 2008   [17] | EU_DAP | School universal | Austria, Belgium, Germany, Greece, Italy, Spain and Sweden | 3-6 months | Comprehensive social influence approach | 18 months | Interactive lessons involving group work, games, quizzes, role play, presentations, discussions and homework | Aged 12 to 14 years | Use of tobacco, alcohol and other drugs | Yes, quantitative methods only. Acceptability, reach and fidelity data. |  |  |  |  |  |  |  |
|  |  |  |  |  |  |  |  |  |  |  |  | | | | | | |
| Guo 2015* [18] | Life Skills + TPD Taiwan - Guo 2015 | School universal | Taiwan | 6-12 months | Theory of planned behaviour | 18 months | Curriculum, group discussion, roleplay, homework, worksheets, behavioural monitoring | Intervention: mean = 13.39 (SD=.55), Control: mean=13.43(SD=.60) | Illicit drug use | Yes, quantitative methods only. Acceptability, reach and fidelity data. |  |  |  |  |  |  |  |
|  |  |  |  |  |  |  |  |  |  |  |  | | | | | | |
| Hall 2013 [19] | Too Good for Drugs | School universal | USA | <3 months | Social influences-based prevention approach, social development model and social learning theory | 6 months | Curriculum, cooperative learning, role playing, small group activities, interactive games, demonstrations, class discussions, iterative practice, homework | Aged 11-14 years mean. For treatment is 11.32, for control 11.34 | Tobacco, alcohol, marijuana, inhalants and street drug use | Yes, quantitative methods only. Reach and fidelity data. |  |  |  |  |  |  |  |
|  |  |  |  |  |  |  |  |  |  |  |  | | | | | | |
| Hecht 2003 [20] | Keepin it Real | School universal | USA | 12-24 months | Tobler's theory on intervention components, narrative theory, social learning theory, focus theory of norms, and cultural ecology theory. | 14 months | Curriculum, homework assignments, performance-based elements, videotapes, role-plays, television and radio public service advertisements (PSAs) and billboard campaigns. Booster sessions: School assemblies, poster projects, murals, neighbourhood nights outs, essay contests, community service activities and webpage design. Videos of successful drug resistance, examples and exercises using culturally appropriate techniques and scenarios. | Age 11 to 18 years. Mean: 12.53 (SD 0.65) | Tobacco, alcohol and drugs | Yes, quantitative and qualitative methods. Acceptability, reach and fidelity data. |  |  |  |  |  |  |  |
|  |  |  |  |  |  |  |  |  |  |  |  | | | | | | |
| Hecht 2008 [21] | Keepin' it Real Plus () | School universal | USA | <3 months | Communication competence theory | 8 months | Curriculum, roleplay/rehearsal of refusal strategies | Mean: 13.01 (SD=0.44) | Alcohol, tobacco and marijuana use | No |  |  |  |  |  |  |  |
|  |  |  |  |  |  |  |  |  |  |  |  | | | | | | |
| Hernandez-Serrano 2013* [22] | Saluda | School universal | Spain | <3 months | Social learning theory | 6 months | Curriculum, role-play, homework, public commitment | Aged 14 to 16 years. Mean: 14.19 (SD = .74), | Alcohol, marijuana and other illicit drug use | No |  |  |  |  |  |  |  |
|  |  |  |  |  |  |  |  |  |  |  |  | | | | | | |
| Huang 2013 [23] | Life Skills + TPD Taiwan - Huang 2013 | School universal | Taiwan | 3-6 months | Theory of planned behaviour | 6 months | Curriculum, roleplay, homework, group discussion, films, quizzes, games | Mean intervention: 13.1(1.68), conventional control: 13.4(0.5), control: 13.4(1.85) | Drug use | No |  |  |  |  |  |  |  |
|  |  |  |  |  |  |  |  |  |  |  |  | | | | | | |
| Malmberg 2014* [24] | Healthy school and drugs | School universal | Netherlands | 24+ months | Theory of Reasoned Action and the Social Cognitive Theory | 32 months | Interactive tasks, ﬁlms, animations, group discussion, chat forums, curriculum,. Integrated condition: As well as the digital e-learning modules, the participants in the integral condition received three additional intervention components (i.e., parental participation, regulation, and monitoring and counselling). | Aged 11 to 15 years. Mean: 13.01 (SD:0.49) range: | Alcohol, tobacco and marijuana use |  |  |  |  |  |  |  |  |
|  |  |  |  |  |  |  |  |  |  |  |  | | | | | | |
| Marsiglia 2015 [25] | Keepin it Real (Mexico) | School universal | Mexico | <3 months | Communication competence theory | 8 months | Curriculum, roleplay/rehearsal of refusal strategies | Average age 13 years, range 12 to 15 | Alcohol, tobacco and marijuana use | No |  |  |  |  |  |  |  |
|  |  |  |  |  |  |  |  |  |  |  |  | | | | | | |
| McCambridge  2011* [26] | Motivational interviewing 2011 | School universal | UK | <3 months | N/S | 12 months | Discuss hypothetical situations | Mean: Intervention: 17.5 Control: 17.6 | Alcohol, tobacco and cannabis consumption | Yes, quantitative and qualitative methods. Acceptability, reach and fidelity data. |  |  |  |  |  |  |  |
|  |  |  |  |  |  |  |  |  |  |  |  | | | | | | |
| Midford 2014* [27] | DEVS | School universal | Australia | 12-24 months | Social learning theory, poststructuralist subjectivity theory, and cognitive dissonance theory | 33 months | Taught curriculum, roleplay, small group discussion, quizzes, workbooks, homework (to be completed with parent, e.g. discussions) | Average 13 years | Alcohol, tobacco, marijuana and other illicit drug use | No |  |  |  |  |  |  |  |
|  |  |  |  |  |  |  |  |  |  |  |  | | | | | | |
| Moskowitz 1984 [28] | Drug education program 1980 (The Napa Project) | School universal | USA | 3-6 months | N/S | 12 months | Curriculum, group discussion, didactic information, role-play and tasks | N/S | Alcohol, tobacco and marijuana | Yes, quantitative methods only. Acceptability and fidelity data. |  |  |  |  |  |  |  |
|  |  |  |  |  |  |  |  |  |  |  |  | | | | | | |
| Newton 2009 [29] | Climate schools | School universal | Australia | 6-12 months | Social influence approach | 12 months | Classroom activities, internet us, eSchool health curriculum: Internet cartoon-based educational programme and teacher-led activity | Mean: 13.08 years (SD 0.58) | Alcohol and cannabis use | Yes, quantitative methods only. Acceptability and reach data. |  |  |  |  |  |  |  |
|  |  |  |  |  |  |  |  |  |  |  |  | | | | | | |
| Okamoto 2016 [30] | Ho'ouna Pono | School universal | USA | <3 months | N/S | 6 months | Curriculum, video depicting drug-related problem situations and examples of resistance, group discussion, interactive activities | Mean = 11.7 (SD=0.65) | Drugs and alcohol use | No |  |  |  |  |  |  |  |
|  |  |  |  |  |  |  |  |  |  |  |  | | | | | | |
| Ringwalt 2009* [31] | ALERT Ringwalt | School universal | USA | 12-24 months | Health belief, social learning and self-efficacy | 12 months | Guided class discussions, small group activities, role playing exercises, videos. | Sixth grade | Cigarettes, alcohol, marijuana and inhalants | Yes, quantitative and qualitative methods. Acceptability, reach and fidelity data. |  |  |  |  |  |  |  |
|  |  |  |  |  |  |  |  |  |  |  |  | | | | | | |
| Sanchez 2017* [32] | #Tamojunto Drug Prevention Program | School universal | Brazil | <3 months | Complex model that integrates theories such as social learning theory, problem behaviour theory, the health belief model, the theory of reasoned action, and social norms theory | 21 months | Curriculum | 58.3% between 11 and 12, 41.7% between 13-15.  Means age of the adolescents was 12.5 (SD 0.7) | Alcohol, tobacco, inhalant, marijuana, cocaine, and crack use | Yes, quantitative methods only. Acceptability, reach and fidelity data. |  |  |  |  |  |  |  |
|  |  |  |  |  |  |  |  |  |  |  |  | | | | | | |
| Seal 2006 [33] | LST - Seal 2006 | School universal | Thailand | Unclear | N/S. However it is a LST intervention, which is based on theory | 6 months | Instruction, demonstration, feedback, role-playing, presentation, and games. The program materials also included videotapes and a life skill booklet. | Intervention: 15.62 Control: 15.49 | Tobacco and drugs | No |  |  |  |  |  |  |  |
|  |  |  |  |  |  |  |  |  |  |  |  | | | | | | |
| Sloboda 2009* [34] | ASAP | School universal | USA | 24+ months | Constructivist learning theory | 4 years | Discussion, problem solving, curriculum, role-play, small group activities and homework | Mean: Intervention: 12.4 (SD 0.66) years, control 12.5 (SD0.68) years | Tobacco, alcohol, marijuana and other drug use | Yes, both quantitative and qualitative  methods. Acceptability, reach and fidelity data. |  |  |  |  |  |  |  |
|  |  |  |  |  |  |  |  |  |  |  |  | | | | | | |
| Smith 2004 [35] | LST - Smith | School universal | USA | 24+ months | N/S. However it is a LST intervention, which is based on theory | 3 years | Intervention 1: Standard LST curriculum  Intervention 2: infused LST - Subject-specific lessons with all core LST principals and methods which were integrated in at least one subject specific lesson | N/S | Tobacco, alcohol and marijuana | Yes, both quantitative and qualitative  methods. Acceptability, reach and fidelity data. |  |  |  |  |  |  |  |
|  |  |  |  |  |  |  |  |  |  |  |  | | | | | | |
| Snow 1992* [36] | Adolescent Decision Making Programme | School universal | USA | <3 months | Skills enhancement and social cognitive approach | 2 years | Curriculum, presentation (didactic information), brainstorming exercises, discussions, role plays and group tasks | N/S | Tobacco, alcohol, marijuana | Yes, unclear methods. Reach measured. |  |  |  |  |  |  |  |
|  |  |  |  |  |  |  |  |  |  |  |  | | | | | | |
| St Pierre 2005*   [37] | ALERT St Pierre | School universal | USA | 6-12 months | N/S | 12 months | Curriculum and small group activities | Seventh grade | tobacco, alcohol, marijuana, inhalants | Yes, both quantitative and qualitative  methods. Acceptability, reach and fidelity data. |  |  |  |  |  |  |  |
|  |  |  |  |  |  |  |  |  |  |  |  | | | | | | |
| Sun 2008* [38] | Towards no drug abuse D | School universal | USA | <3 months | Cognitive misperception correction and behavioural skills instruction | 12 months | Curriculum, didactic information and activities | Intervention 1: 15.17 Intervention 2: 15.43 Control: 15.18 | Tobacco, alcohol, marijuana and hard drugs | Yes, methods unclear. Acceptability and reach data. |  |  |  |  |  |  |  |
|  |  |  |  |  |  |  |  |  |  |  |  | | | | | | |
| Sussman* 2012 [39] | Project Towards no Drug Abuse | School universal | USA | <3 months | Social influence theory and health motivation decision making model | 12 months | Curriculum, didactic information and listening  MI component: up to three brief collaborative MI sessions | Aged 14 to 21 years. Mean 16.8 (SD: 0.93) | Alcohol, tobacco, marijuana and hard drug use | Yes, quantitative methods only. Acceptability, reach and fidelity data. |  |  |  |  |  |  |  |
|  |  |  |  |  |  |  |  |  |  |  |  | | | | | | |
| Vogl 2014 [40] | Climate schools | School universal | Australia | <3 months | Social influence approach | 10 months | Curriculum, individual interactive study (internet based), class activities | Mean 15.44 (SD, 0.41) | Psychostimulants and marijuana use | Yes, quantitative methods only. Acceptability, reach and fidelity data. |  |  |  |  |  |  |  |
|  |  |  |  |  |  |  |  |  |  |  |  | | | | | | |
| Weichold 2012 [41] | IPSY | School universal | Germany | <3 months | WHO's life skills approach as well as on theories and empirical findings concerning the aetiology of adolescent substance use | 3 years | Curriculum, group discussion, roleplay | Mean 10.74 (SD=0.48) | Alcohol and tobacco use | Yes, quantitative and qualitative methods. Acceptability, reach and fidelity data. |  |  |  |  |  |  |  |
|  |  |  |  |  |  |  |  |  |  |  |  | | | | | | |
| White 2017*  [42] | Assist+FRANK | School universal | Wales | 12-24 months | Diffusion of innovations theory | 18 months | Informal conversations between peers on drug-use harms | Mean 12.6 (SD=0.3) | Illicit drug use (marijuana, cocaine, amphetamines, crack, ecstasy, aerosols, gas, glue, legal highs, poppers, nitrous oxide, sedatives or sleeping pills, LSD, magic mushrooms, ketamine, steroids, methadrone, other prescription drugs, and opioids) | Yes, quantitative and qualitative methods. Acceptability, reach and fidelity data. |  |  |  |  |  |  |  |
|  |  |  |  |  |  |  |  |  |  |  |  | | | | | | |
| **School targeted** | | | | | | | | | | |  | | | | | | |
| Hallfors 2006   [43] | Reconnecting youth | School targeted | USA | 3-6 months | N/S | 6 months | Curriculum and practising skills. Students practiced skills related to the 4 main themes and their goals and also practiced giving  and receiving positive social support | N/S | Alcohol, marijuana and cigarettes | Yes, quantitative methods only. Acceptability, reach and fidelity data. |  |  |  |  |  |  |  |
|  |  |  |  |  |  |  |  |  |  |  |  | | | | | | |
| Horan 1982 [44] | Assertion training | School targeted | USA | <3 months | Classical decision theory | 3 years | Instruction, role play with training stimuli. Didactic information, live modelling, role-play and feedback | N/S | Alcohol, tobacco and marijuana | Yes, quantitative methods only. Acceptability and reach data. |  |  |  |  |  |  |  |
|  |  |  |  |  |  |  |  |  |  |  |  | | | | | | |
| McCambridge  2005* [45] | Motivational interviewing 2005 | School targeted | UK | <3 months | N/S | 12 months | Highly interactive discussions, reflective listening, affirmation, open questions  and summaries, in order to elicit ‘change talk’ | Aged 16 to 20 years | Alcohol, tobacco, cannabis and other illicit drugs | No |  |  |  |  |  |  |  |
|  |  |  |  |  |  |  |  |  |  |  |  | | | | | | |
| McCambridge  2008* [46] | Motivational interviewing 2008 | School targeted | UK | <3 months | N/S | 6 months | Discussions, 'empathic' listening and issue resolution. Eliciting participants view of their situation, consideration of costs and benefits, self-monitoring. | Mean: Intervention: 18 (1.0) Control: 17.9 (1.7) | Alcohol, tobacco, cannabis and other illicit drugs | Yes, quantitative and qualitative methods. Acceptability, reach and fidelity data. |  |  |  |  |  |  |  |
|  |  |  |  |  |  |  |  |  |  |  |  | | | | | | |
| Sussman 1998* [47] | Towards no drug abuse A | School targeted | USA | 3-6 months | Social Influence theory | 13.5 months | Listening skills, information on chemical dependency, coping skills, self-control skills. Thinking of 'school as a community'. Both: Curriculum, didactic information and listening; Intervention 2: School meetings, events and community newsletters | Aged 14 to 19. 93% were 16 to 18 years. Mean = 16.7 (SD=0.8) | Tobacco, alcohol, marijuana and hard drugs | Yes, methods unclear. Acceptability, reach and fidelity data. |  |  |  |  |  |  |  |
|  |  |  |  |  |  |  |  |  |  |  |  | | | | | | |
| Sussman 2003* [48] | Towards no drug abuse B | School targeted | USA | Unclear | Motivation-skills-decision-making model | 2 years | Both: Curriculum and didactic information. Intervention 2: Self-instruction workbook. | N/S | Tobacco, alcohol, marijuana and hard drugs | Yes, methods unclear. Acceptability and reach data. |  |  |  |  |  |  |  |
|  |  |  |  |  |  |  |  |  |  |  |  | | | | | | |
| Valente 2007* [49] | Towards no drug abuse C | School targeted | USA | <3 months | Social influence theory and motivation-skills-decision-making model | 12 months | Both: Curriculum and review game; Intervention 1: Interactive discussion; Intervention 2: Small-group activities, peer leader, group discussion and role-play | Aged 14 to 17 years | Tobacco, alcohol, marijuana and cocaine | Yes, methods unclear. Acceptability and reach data. |  |  |  |  |  |  |  |
|  |  |  |  |  |  |  |  |  |  |  |  | | | | | | |
| **Family universal** | | | | | | | | | | |  | | | | | | |
| Bauman 2002* [50] | Family matters | School universal | USA | <3 months | Value expectancy theory, Fishbein's theory of reasoned action, Bandura's social learning theory, health belief model, socialisation theory, social control theory, social development theory, family interaction theory and social inoculation theory | 12 months | Four booklets to adult family members and  follow up telephone contacts by health educators | Mean: 13.9 years (SD =0.9) | Tobacco and alcohol | Yes, qualitative and quantitative measures. Acceptability, reach and fidelity. |  |  |  |  |  |  |  |
|  |  |  |  |  |  |  |  |  |  |  |  | | | | | | |
| Byrnes 2012 [51] | Family matters vs SFP | Family universal | USA | Unclear | For SFP: biopsychosocial vulnerability model; FM: Social learning theory, Theory of reasoned action, value expectation theory | 2 years | SFP: Curriculum, group discussion, quizzes and games (child sessions), skills practice; FM: programme booklet, monitoring by professionals, parent-child discussions and activities, homework | Mean = 11.54 (SD=0.53) | Alcohol, tobacco and other drug use | Yes, quantitative methods only. Acceptability, reach and fidelity data. |  |  |  |  |  |  |  |
|  |  |  |  |  |  |  |  |  |  |  |  | | | | | | |
| Schinke 2009* [52] | Mother-Daughter - Non-Specific | Family universal | USA | <3 months | Family interaction theory | 12 months | Joint mother-daughter participation of web-based programme: Voice-over narration,  skills demonstrations and interactive exercises. animated vignettes and video demonstrations | Mean 12.76 (1.0) | Alcohol, cigarettes, marijuana, illicit prescription drugs and inhalants | Yes, methods unclear. Acceptability, reach and fidelity data. |  |  |  |  |  |  |  |
|  |  |  |  |  |  |  |  |  |  |  |  | | | | | | |
| Skeer 2016 [53] | SUPPER | Family universal | USA | <3 months | Eco-developmental Theory | 6 months | Psycho-education, handbook, brief intervention | 56.3% children in 3/4 grade, 43.7% in 5/6 grade | Alcohol, marijuana and other drug use | Yes, quantitative and qualitative methods. Acceptability and reach |  |  |  |  |  |  |  |
|  |  |  |  |  |  |  |  |  |  |  |  | | | | | | |
| **Family targeted** | | | | | | | | | | |  | | | | | | |
| Fang 2010* [54] | Mother-Daughter - Asian-American | Family targeted | USA | <3 months | Family interaction theory | 2 years | Voice over narration,  animated graphics, and games, session content involved skill demonstrations and interactive exercises that required the joint participation of mothers and daughters | Mean: Whole sample 13.1 (0.96) (Control: 13.18  (0.96) Intervention: 13.03 (0.95)) | Alcohol, cigarettes, marijuana and illicit prescription drugs | No |  |  |  |  |  |  |  |
|  |  |  |  |  |  |  |  |  |  |  |  | | | | | | |
| **Individual interventions** | | | | | | | | | | | | | | | | | |
| Schwinn 2010* [55] | RealTeen 2010 | Individual universal | USA and Canada | <3 months | N/S | 6 months | 12 intervention sessions which were guided by an older female animated character. Plus a homepage which provided access to chat forums, news feeds etc. Didactic information, practise exercises, goal-setting, quizzes, blogs, pen pal assignment, public chat forum and diary | Average age 14 years | Alcohol, cigarettes, marijuana, cocaine, inhalants, methamphetamines and ecstasy | Yes, quantitative methods only. Acceptability, reach and fidelity data. |  |  |  |  |  |  |  |
|  |  |  |  |  |  |  |  |  |  |  |  | | | | | | |
| Schwinn 2018* [56] | RealTeen 2018 | Individual universal | USA | 3-6 months | Social learning theory, resiliency framework | 12 months | Interactive internet-based programme - taught content with interactive exercises and quizzes | Mean 13.7 years (SD=0.67) | Alcohol, tobacco, marijuana, other illicit drug use | Yes, quantitative methods only. Acceptability and reach. |  |  |  |  |  |  |  |
|  |  |  |  |  |  |  |  |  |  |  |  | | | | | | |
| **Individual targeted** | | | | | | | | | | |  | | | | | | |
| D'Amico 2013* [57] | Free Talk | Individual targeted | USA | 3-6 months | N/S | 180 days | Group motivational interviewing, psycho-education | Mean 16.6 years (SD = 1.05) | Alcohol and other drug use | Yes, quantitative methods only. Acceptability, reach and fidelity data. |  |  |  |  |  |  |  |
|  |  |  |  |  |  |  |  |  |  |  |  | | | | | | |
| **Combination universal** | | | | | | | | | | |  | | | | | | |
| Komro 2008   [58] | Project Northland | School and family universal | USA | 24+ months | Triadic influence and Perry's planning model for adolescent health promotion progarmmes integrate factors from several socio-behavioural theories | 3 years | Peer-led curriculum, parent involvement and education, peer leadership, youth-led community service projects and community organising and environmental neighbourhood change. Components are implemented consecutively across time. | N/S | Alcohol and drugs | Yes, quantitative and qualitative methods. Acceptability, reach and fidelity data. |  |  |  |  |  |  |  |
|  |  |  |  |  |  |  |  |  |  |  |  | | | | | | |
| Marsiglia 2019 [59] | Keepin' it Real + FPNG | School and family universal | USA | <3 months | Narrative theory, theory of norms, ecological risk and resilience approach, communication competence theory. FPNY guided by Eco-developmental theory | 18 months | School-based: curriculum, role-play; parent-based: didactic teaching, small group discussion, role-play, video, reflection, homework to complete with adolescent | Mean: Control: 12.32 (SD 0.54); YO: 12.14 (sd 0.43), PY: 12.13 (SD 0.43) (study total: 12.20, SD 0.48) | Alcohol, tobacco and other drug use | Yes, quantitative methods only. Acceptability and reach data. |  |  |  |  |  |  |  |
|  |  |  |  |  |  |  |  |  |  |  |  | | | | | | |
| Roberts* 2011 [60] | Aussie optimism | School and family universal | Australia | 12-24 months | Social and cognitive life skills | 12 months | Curriculum, student and parent workbooks, didactic information, interactive activities, role-play, games, behavioural experiments, co-operative learning, families and parents self-directed booklet | Mean (SD) - AOP: 10.91 (0.32) AOP+: 10.91 (0.34) Control: 10.93 (0.33) | Tobacco and alcohol use | Yes, methods unclear. Acceptability, reach and fidelity data. |  |  |  |  |  |  |  |
|  |  |  |  |  |  |  |  |  |  |  |  | | | | | | |
| Schaps 1982   [61] | Drug education program | School and family universal | USA | Unclear | Lasswell's framework for motives and needs Value/Decision-making and Knowledge/Attitudes approaches | 12 months | Curriculum, didactic information, tasks, anonymous questions and parent evening course | N/S | Tobacco, alcohol, marijuana | Yes, quantitative and qualitative methods. Acceptability, reach and fidelity data. |  |  |  |  |  |  |  |
|  |  |  |  |  |  |  |  |  |  |  |  | | | | | | |
| Skarstrand 2013* [62] | Swedish SFP | School family universal | Sweden | <3 months | The bio psychosocial vulnerability model, a resiliency model and a family process model linking economic stress and adolescent adjustment | 3 years | Youth sessions: role play, practical skills training, games, homework. Parent component: modelling, homework. Family sessions: skills practice, family activities | Average age 12 years | Substance use (ATOD) | Yes, quantitative methods only. Acceptability, and reach. |  |  |  |  |  |  |  |
|  |  |  |  |  |  |  |  |  |  |  |  | | | | | | |
| **Combination targeted** | | | | | | | | | | |  | | | | | | |
| Clark 2010 [63] | Success | School, individual, family and community targeted | USA | Unclear | N/S | 12 months | Didactic information, individual/group counselling, parent communication and community agency referrals. Project SUCCESS consists of four components: (1) the Prevention  Education Series, a four-topic substance use prevention program taught to small groups in 6 to 8 weekly sessions; (2) individual and group counselling; (3) communications with parents; and (4) referrals to community agencies | Mean: Control: 16.64 Intervention: 16.79 | Alcohol, marijuana, cigarettes and other illegal drugs | Yes, quantitative methods only. Acceptability, reach and fidelity data. |  |  |  |  |  |  |  |
|  |  |  |  |  |  |  |  |  |  |  |  | | | | | | |
| Elder 2002 [64] | Migrant education | School and family targeted | USA | <3 months | N/S | 2 years | Presentation of information, modelling, and behavioural rehearsal. Financial incentive, booster sessions, newsletter, homework. First aid/home safety educational and skills-training programmes: Didactic information, modelling, behavioural rehearsal, role-playing, group leader-led discussions, videos, demonstrations, skills practise, "booster" telephone calls and newsletters | N/S | Tobacco and  alcohol use | Yes, unclear methods. Acceptability and fidelity data. |  |  |  |  |  |  |  |
|  |  |  |  |  |  |  |  |  |  |  |  | | | | | | |
| Rohrbach 1994 [65] | Midwestern prevention project (Project STAR) | School and family targeted | USA | 12-24 months | Social influence theory | 3.5 years | Establishment of a parent program implementation committee at each school and student peer leaders, two parenting skills workshops, participation in community wide programs. Peer and environmental pressure resistance skills training, problem solving skills for difficult situations, social norm correction, information about health consequences, public statement/affirmation to avoid drugs. Mass media programming, education programme (curriculum, didactic information, parent education parent-child homework exercises, parent-child role-play, organisation at school sites, workshops and participation in broader-based community organisation activities), community organisation and health policy | N/S | Alcohol and tobacco | Yes, methods unclear. Acceptability, reach and fidelity data. |  |  |  |  |  |  |  |
|  |  |  |  |  |  |  |  |  |  |  |  | | | | | | |
| Gilchrist 1987 [66] | Skills enhancement programme | School and community targeted | USA | Unclear | Skills enhancement approaches | 6 months | Didactic information, discussion, activities, games, films, handouts, posters, guest speaker, report of personal experiences, role-play with use of cartoons and stick puppets and advert development | Mean: Intervention: 11.22 Control: 11.46 Overall: 11.34 | Alcohol, marijuana, tobacco and inhalants | No |  |  |  |  |  |  |  |
|  |  |  |  |  |  |  |  |  |  |  |  | | | | | | |

^a^ Risk of bias assessment referring to seven domains. See figure legend for details of each domain and colour coding.

*Legend: Descriptive characteristics of studies included in the systematic review: Primary author names and reference; study name; level of intervention (school, family, individual, combination and universal or targeted); country; duration of intervention; theory; intervention components; follow-up duration; age of participants; behaviours targeted; process evaluation; the risk of bias assessment. Table 1 key for the risk of bias assessment:*

| *Low risk of bias* |  |
| --- | --- |
| *Unclear risk of bias* |  |
| *High risk of bias* |  |

*Risk of bias assessment refers to the following seven domains, in the order they appear in the table, as instructed by Cochrane:*

*Random sequence generation (selection bias)*

*Allocation concealment (selection bias)*

*Blinding of participants and personnel (performance bias)*

*Blinding of outcome assessment (detection bias)*

*Incomplete outcome data (attrition bias)*

*Selective reporting (reporting bias)*

*Other bias*

1. Bell, R.M., P.L. Ellickson, and E.R. Harrison, Preventive medicine, 1993. **22**(4): p. 463-483

2. Botvin, G.J., et al., Addictive behaviors, 1990. **15**(1): p. 47-63

3. Botvin, G.J., et al., Journal of Consulting and Clinical Psychology, 1990. **58**(4): p. 437-446

4. Botvin, G.J., et al., Psychology of addictive behaviors, 1994. **8**(2): p. 116

5. Botvin, G.J., et al., Prevention Science, 2001. **2**(1): p. 1-13

6. Botvin, G.J. and K.W. Griffin, 2015

7. Butzer, B., et al., Journal of youth and adolescence, 2017. **46**(3): p. 603-632

8. Calafat, A., et al., Adicciones, 1989. **1**(2): p. 96-111

9. Clayton, R.R., A.M. Cattarello, and B.M. Johnstone, Preventive Medicine, 1996. **25**(3): p. 307-318

10. D'Amico, E.J., et al., Psychology of Addictive Behaviors, 2012. **26**(4): p. 994

11. de la Rosa López, Á., *La Prevención de las drogodependencias en el ámbito escolar: una experiencia práctica evaluada*. 1995: Publicacions Universitat de Barcelona.

12. Dent, C.W., S. Sussman, and A.W. Stacy, Preventive Medicine, 2001. **32**(6): p. 514-20. 11394955

13. Donaldson, S.I., J.W. Graham, and W.B. Hansen, Journal of Behavioral Medicine, 1994. **17**(2): p. 195-216

14. Eisen, M., et al., Addictive Behaviors, 2002. **27**(4): p. 619-632

15. Ellickson, P.L., et al., American journal of public health, 2003. **93**(11): p. 1830-1836

16. Evers, K.E., et al., Addictive Behaviors, 2012. **37**(9): p. 1009-1018

17. Faggiano, F., et al., Preventive medicine, 2008. **47**(5): p. 537-543

18. Guo, J.-L., et al., Journal of Adolescent Health, 2015. **56**(3): p. 314-322

19. Hall, B.W., T.P. Bacon, and J.M. Ferron, Journal of drug education, 2013. **43**(3): p. 277-300

20. Hecht, M.L., et al., Prevention Science, 2003. **4**(4): p. 233-248

21. Hecht, M.L., et al., Journal of drug education, 2008. **38**(3): p. 225-251

22. Hernández Serrano, O., et al., 2013

23. Huang, C.M., et al., Journal of school health, 2012. **82**(7): p. 328-335

24. Malmberg, M., et al., Addiction, 2014. **109**(6): p. 1031-1040

25. Marsiglia, F.F., et al., The journal of primary prevention, 2015. **36**(2): p. 93-104

26. McCambridge, J., et al., Drug and alcohol dependence, 2011. **114**(2-3): p. 177-184

27. Midford, R., et al., International Journal of Drug Policy, 2014. **25**(1): p. 142-150

28. Moskowitz, J.M., et al., Journal of Drug Education, 1984. **14**(1): p. 9-22

29. Newton, N.C., et al., Preventive Medicine, 2009. **48**(6): p. 579-84. 19389420

30. Okamoto, S.K., et al., Journal of health care for the poor and underserved, 2016. **27**(2): p. 815

31. Ringwalt, C.L., et al., Archives of pediatrics & adolescent medicine, 2009. **163**(7): p. 625-632

32. Sanchez, Z.M., et al., Prevention Science, 2017. **18**(7): p. 772-782

33. Seal, N., Nurs Health Sci, 2006. **8**(3): p. 164-8. 16911176

34. Sloboda, Z., et al., Drug Alcohol Depend, 2009. **102**(1-3): p. 1-10. 19332365

35. Smith, E.A., et al., Journal of Alcohol and Drug Education, 2004. **48**(1): p. 51

36. Snow, D.L., et al., J Drug Educ, 1992. **22**(2): p. 101-14. 1625111

37. Pierre, T.L.S., et al., Prevention Science, 2005. **6**(4): p. 305

38. Sun, P., et al., Preventive Medicine, 2008. **47**(4): p. 438-42. 18675294

39. Sussman, S., et al., Health Psychol, 2012. **31**(4): p. 476-85. 21988096

40. Vogl, L.E., et al., Substance abuse treatment, prevention, and policy, 2014. **9**(1): p. 1-14

41. Weichold, K. and R.K. Silbereisen, Sucht, 2012. **58**(4): p. 247-258

42. White, J., et al., Public Health Research, 2017. **5**(7): p. 1-126

43. Hallfors, D., et al., American Journal of Public Health, 2006. **96**(12): p. 2254-2259

44. Horan, J.J. and J.M. Williams, American Educational Research Journal, 1982. **19**(3): p. 341-351

45. McCambridge, J. and J. Strang, Addiction, 2005. **100**(4): p. 470-8. 15784061

46. McCambridge, J., R.L. Slym, and J. Strang, Addiction, 2008. **103**(11): p. 1809-18. 18778385

47. Sussman, S., et al., Preventive Medicine, 1998. **27**(4): p. 632-642

48. Sussman, S., et al., Preventive Medicine, 2003. **37**(2): p. 155-162

49. Valente, T.W., et al., Addiction, 2007. **102**(11): p. 1804-15. 17784893

50. Bauman, K.E., et al., Prevention Science, 2002. **3**(1): p. 35-42

51. Byrnes, H.F., B.A. Miller, and N. Laborde, Health education & behavior, 2013. **40**(2): p. 206-215

52. Schinke, S.P., L. Fang, and K.C. Cole, Preventive Medicine, 2009. **49**(5): p. 429-35. 19682490

53. Skeer, M.R., et al., Journal of child and family studies, 2016. **25**(12): p. 3739-3748

54. Fang, L., S.P. Schinke, and K.C. Cole, Journal of Adolescent Health, 2010. **47**(5): p. 529-32. 20970090

55. Schwinn, T.M., S.P. Schinke, and J. Di Noia, Prev Sci, 2010. **11**(1): p. 24-32. 19728091

56. Schwinn, T.M., et al., Journal of youth and adolescence, 2018. **47**(3): p. 490-500

57. D’Amico, E.J. and K. Fromme, Addiction, 2002. **97**(5): p. 563-574

58. Komro, K.A., et al., Addiction, 2008. **103**(4): p. 606-18. 18261193

59. Marsiglia, F.F., et al., Prevention Science, 2019. **20**(7): p. 1125-1135

60. Roberts, C., et al., Advances in Mental Health, 2011. **10**(1): p. 72-82

61. Schaps, E., et al., Journal of Drug Education, 1982. **12**(4): p. 353-364

62. Skärstrand, E., K. Sundell, and S. Andréasson, European journal of public health, 2013. **24**(4): p. 578-584

63. Clark, H.K., et al., Addictive Behaviors, 2010. **35**(3): p. 209-17. 19914003

64. Elder, J.P., et al., American Journal of Preventive Medicine, 2002. **23**(4): p. 269-275

65. Rohrbach, L.A., et al., Journal of Research on Adolescence, 1994. **4**(2): p. 295-317

66. Gilchrist, L.D., et al., International Journal of the Addictions, 1987. **22**(9): p. 869-879
