# Supplementary material for: Individual-, family- and school-based interventions to prevent multiple risk behaviours relating to alcohol, tobacco and drug use in young people aged 8-25 years: a systematic review and meta-analysis
Source: BMC Public Health. 2022 Jun 3;22:1111. doi: 10.1186/s12889-022-13072-5 (PMC9165543; doi:10.1186/s12889-022-13072-5)

**Additional File 8 – Sensitivity analyses for highest and lowest ICCs and analyses using fixed effects models**

**Sensitivity analyses – ICC**

Additional Figure 8.1 short term alcohol use outcome with lowest reported ICC


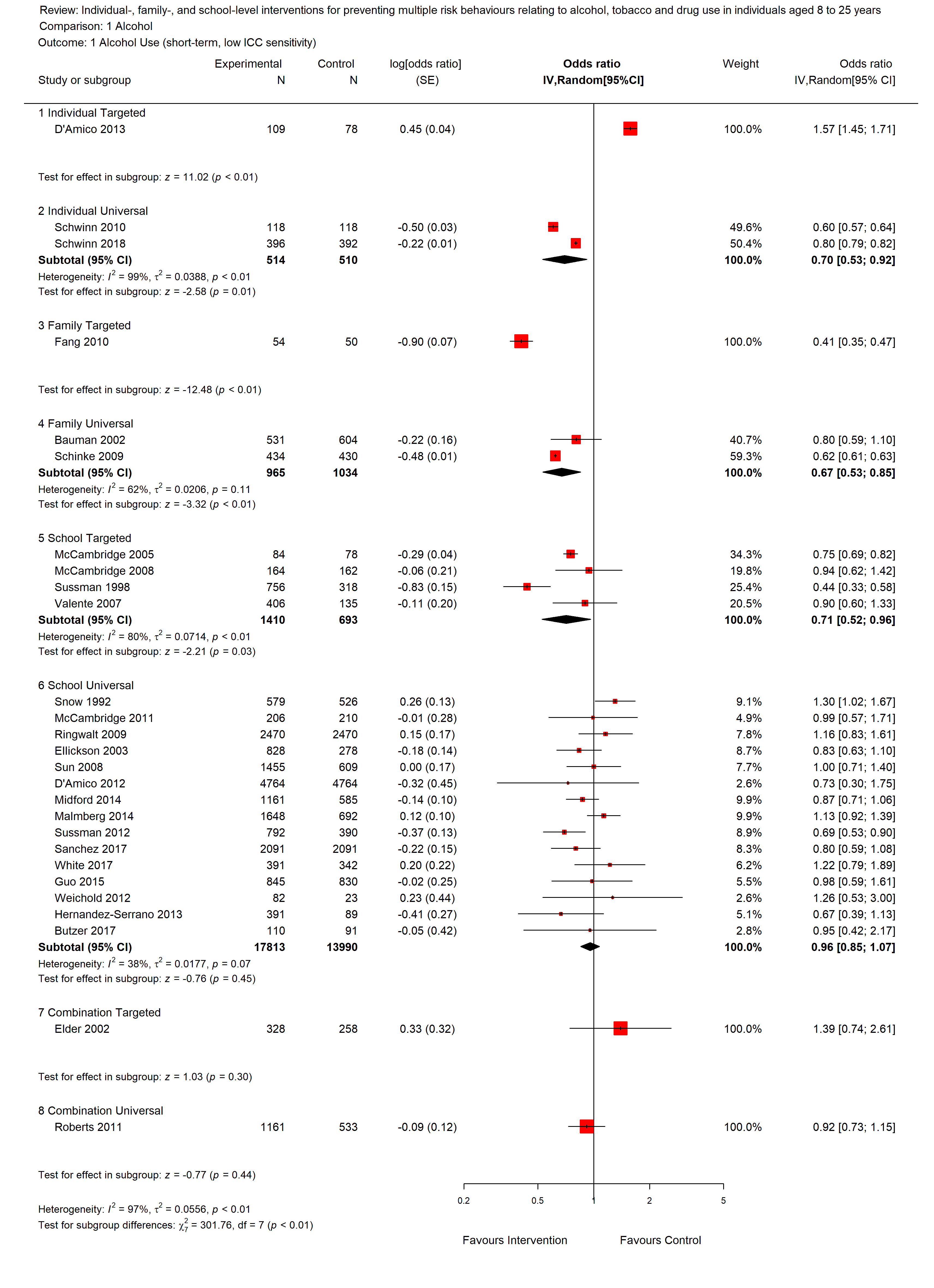


Additional Figure 8.2 short term alcohol use outcome with highest reported ICC


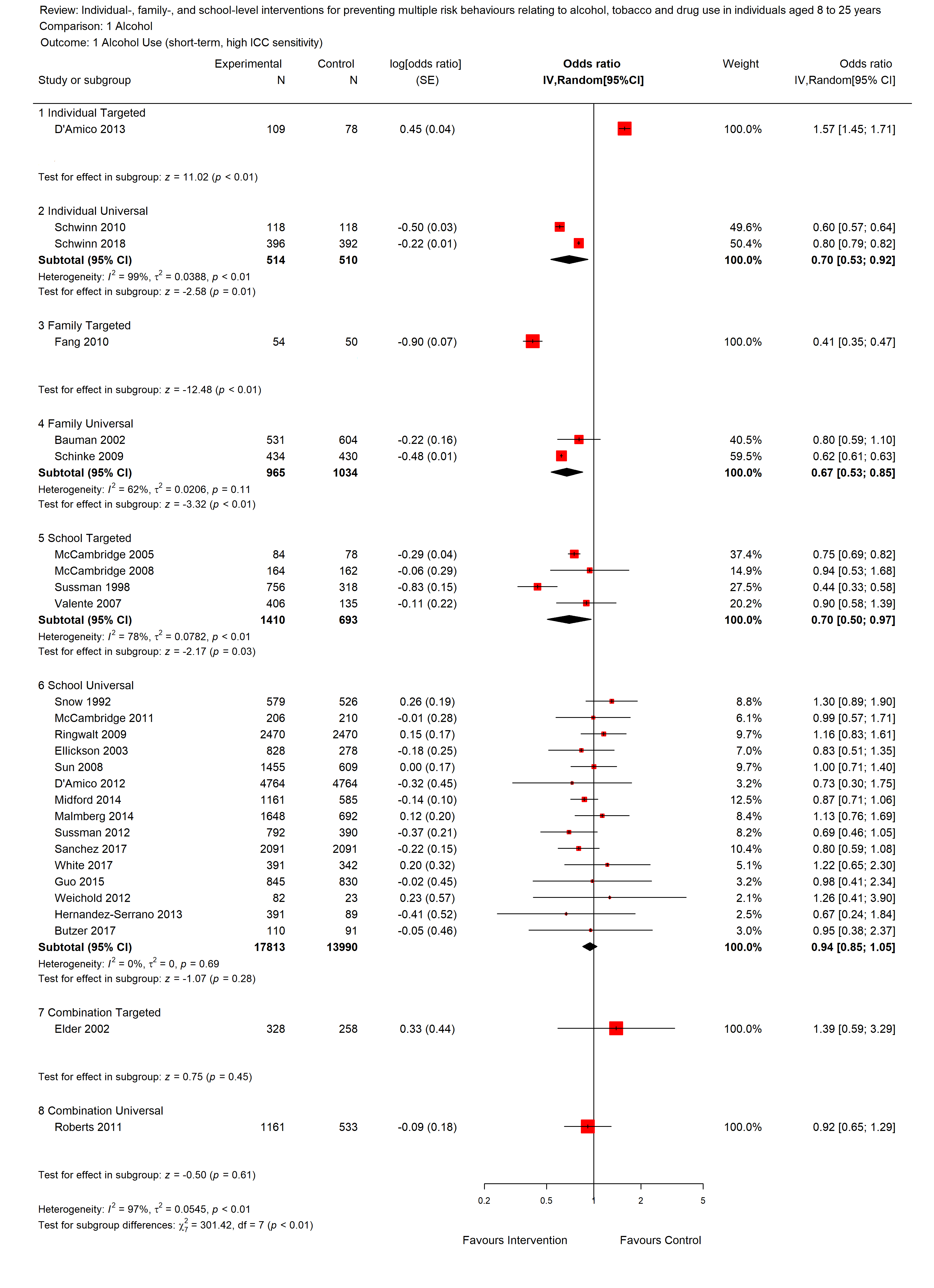


Additional Figure 8.3 long term alcohol use outcome with lowest reported ICC


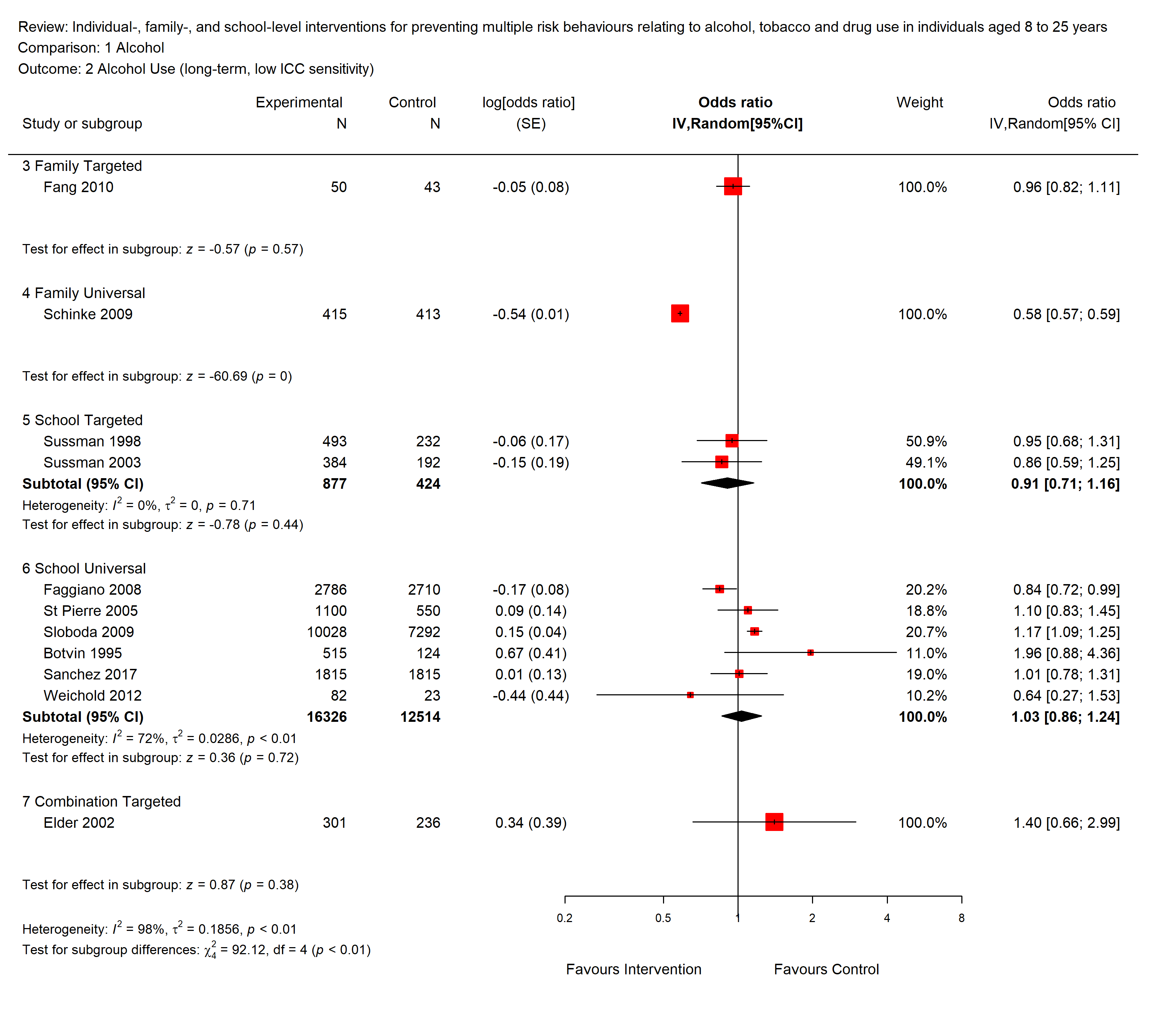


Additional Figure 8.4 long term alcohol use outcome with highest reported ICC


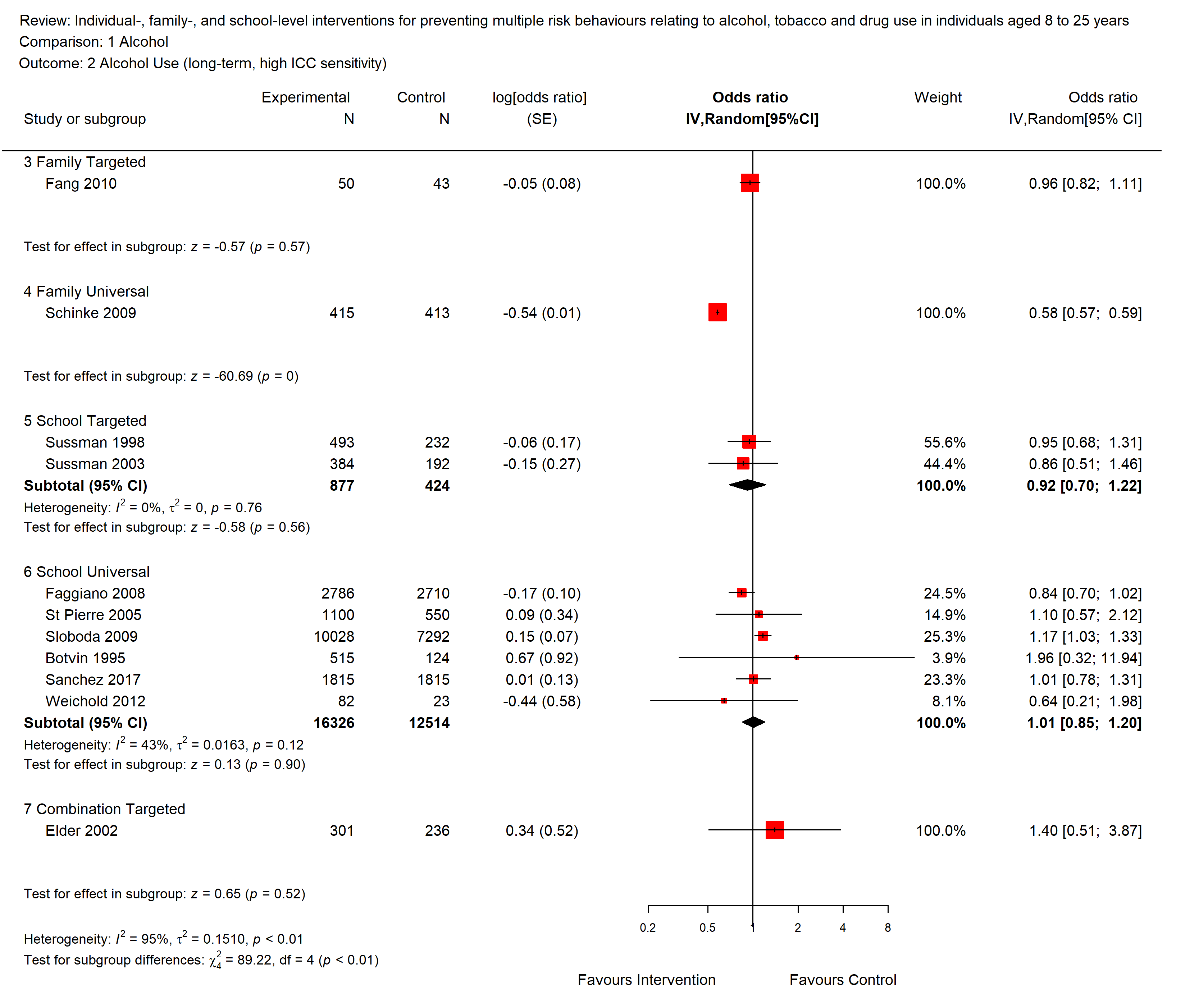


Additional Figure 8.5 short term heavy alcohol use (binge drinking) outcome with lowest reported ICC


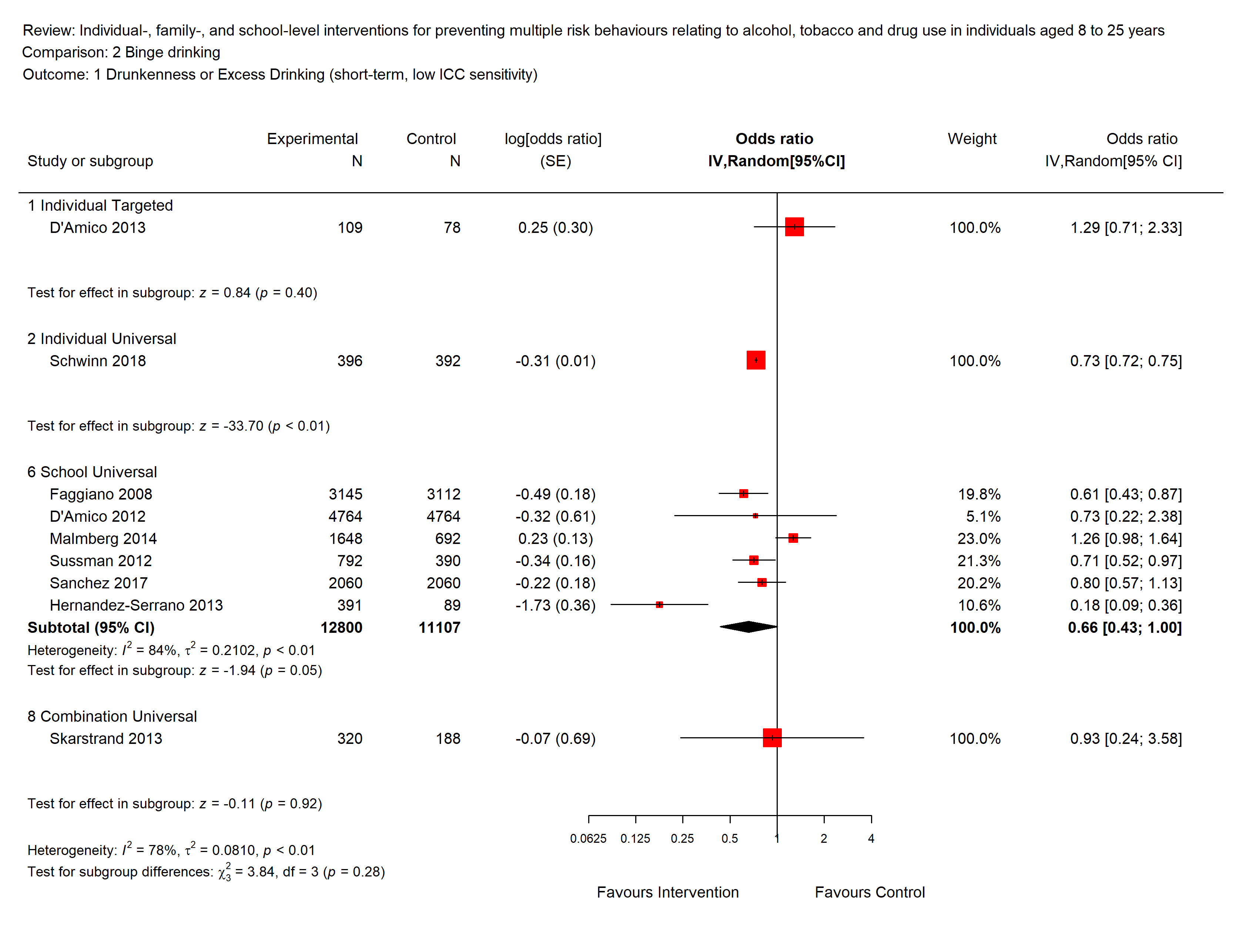


Additional Figure 8.6 short term heavy alcohol use (binge drinking) outcome with highest reported ICC


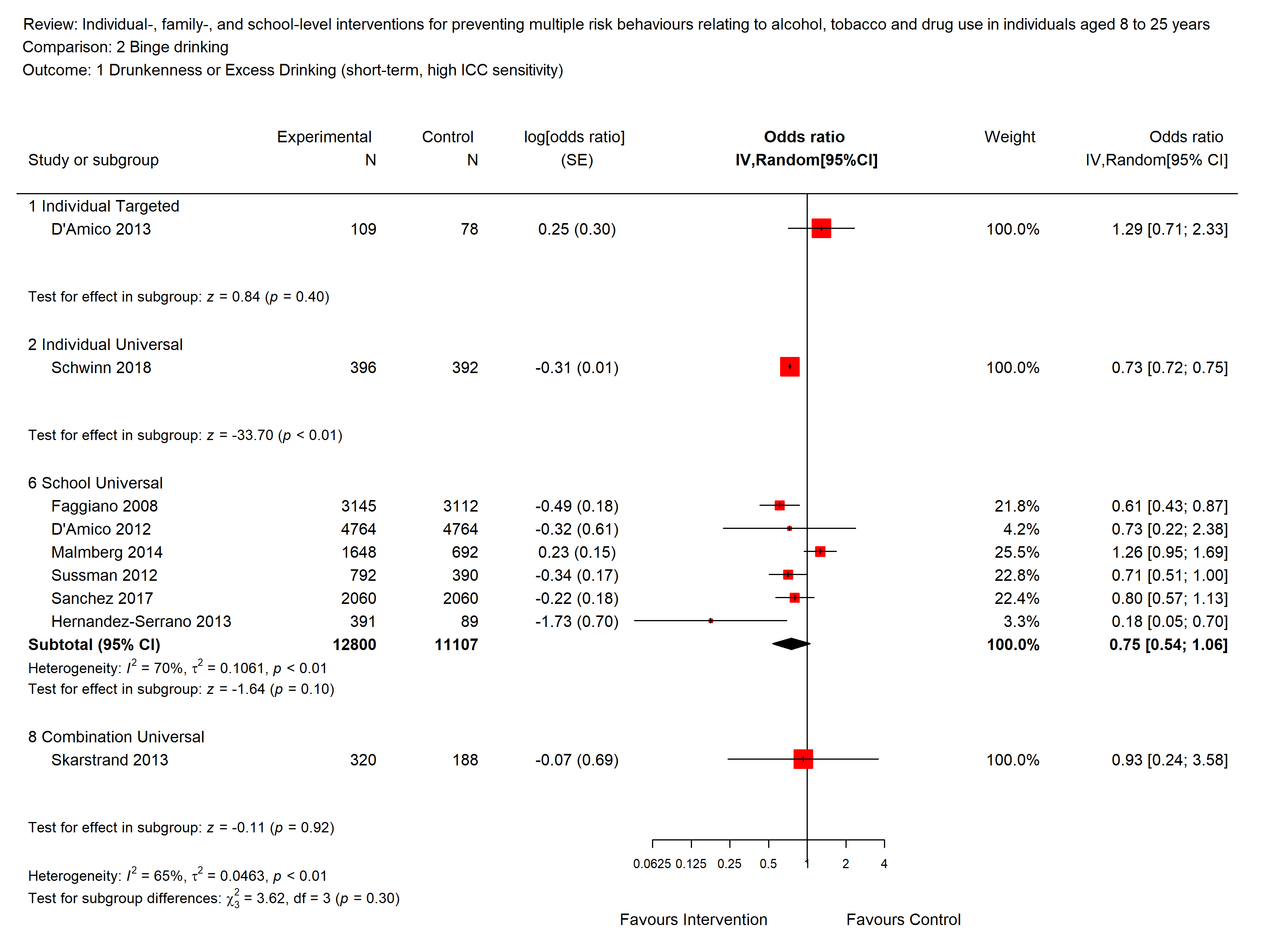


Additional Figure 8.7 long term heavy alcohol use (binge drinking) outcome with lowest reported ICC


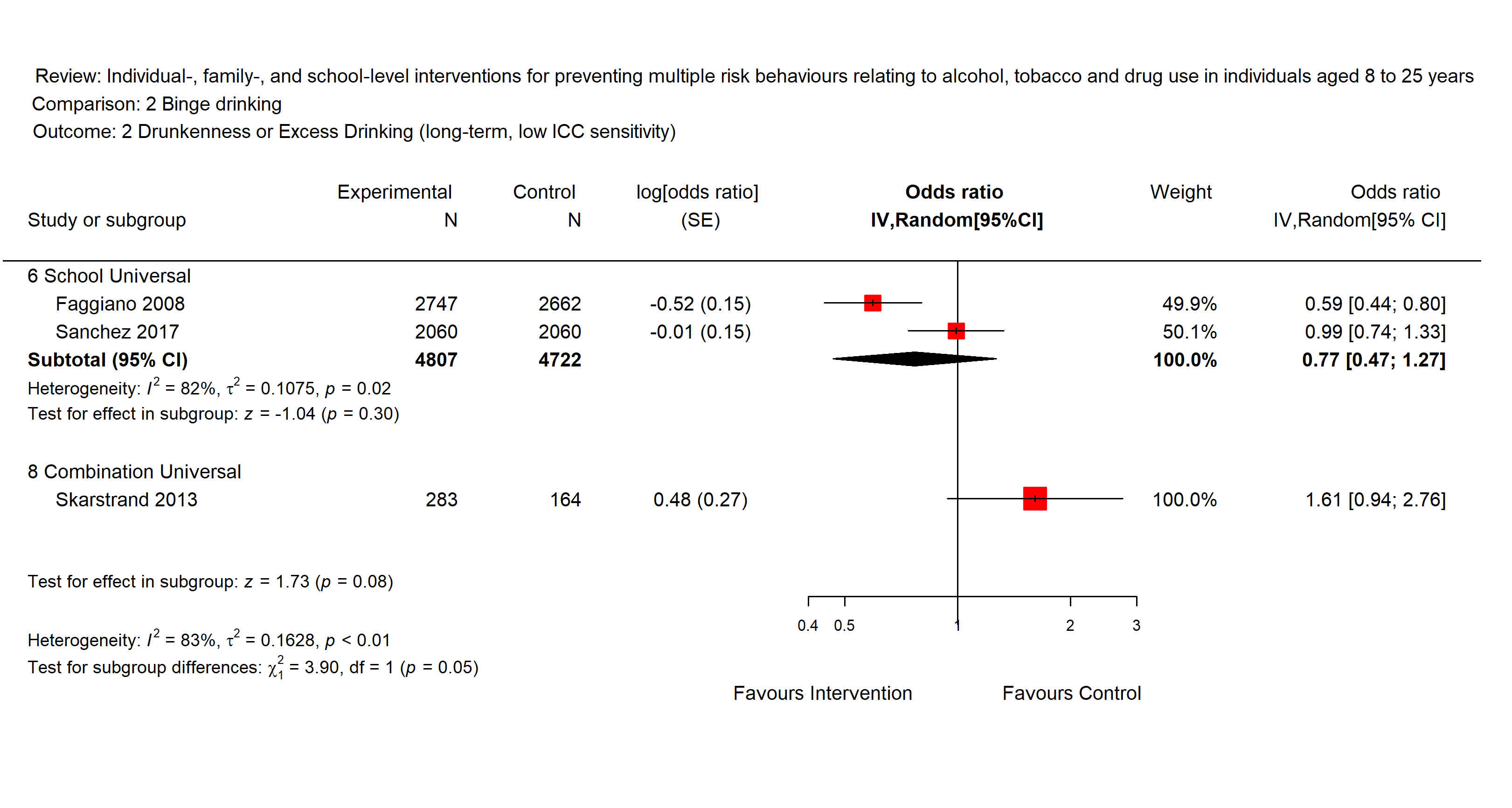


Additional Figure 8.8 long term heavy alcohol use (binge drinking) outcome with highest reported ICC


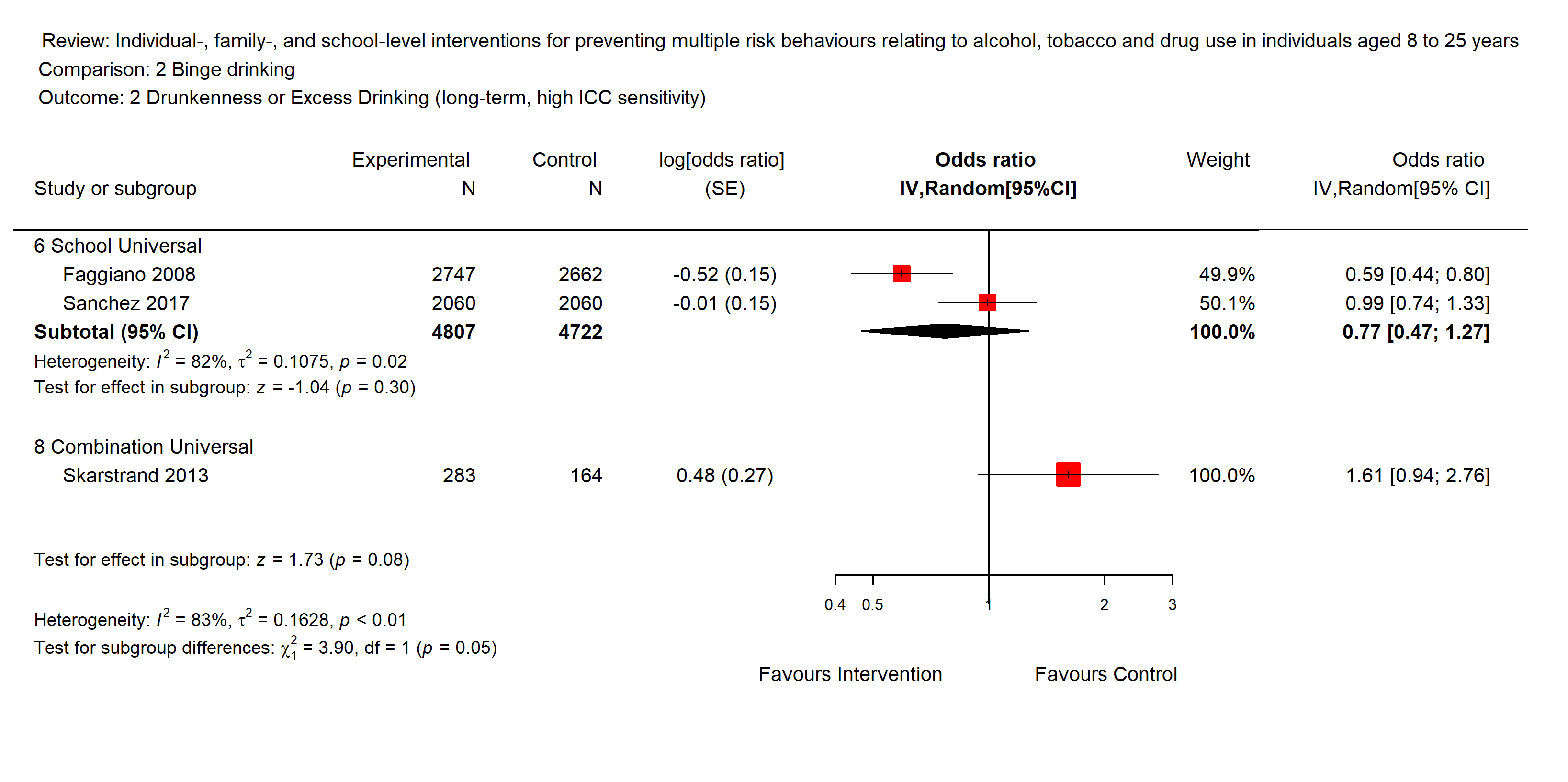


Additional Figure 8.9 short term tobacco use outcome with lowest reported ICC


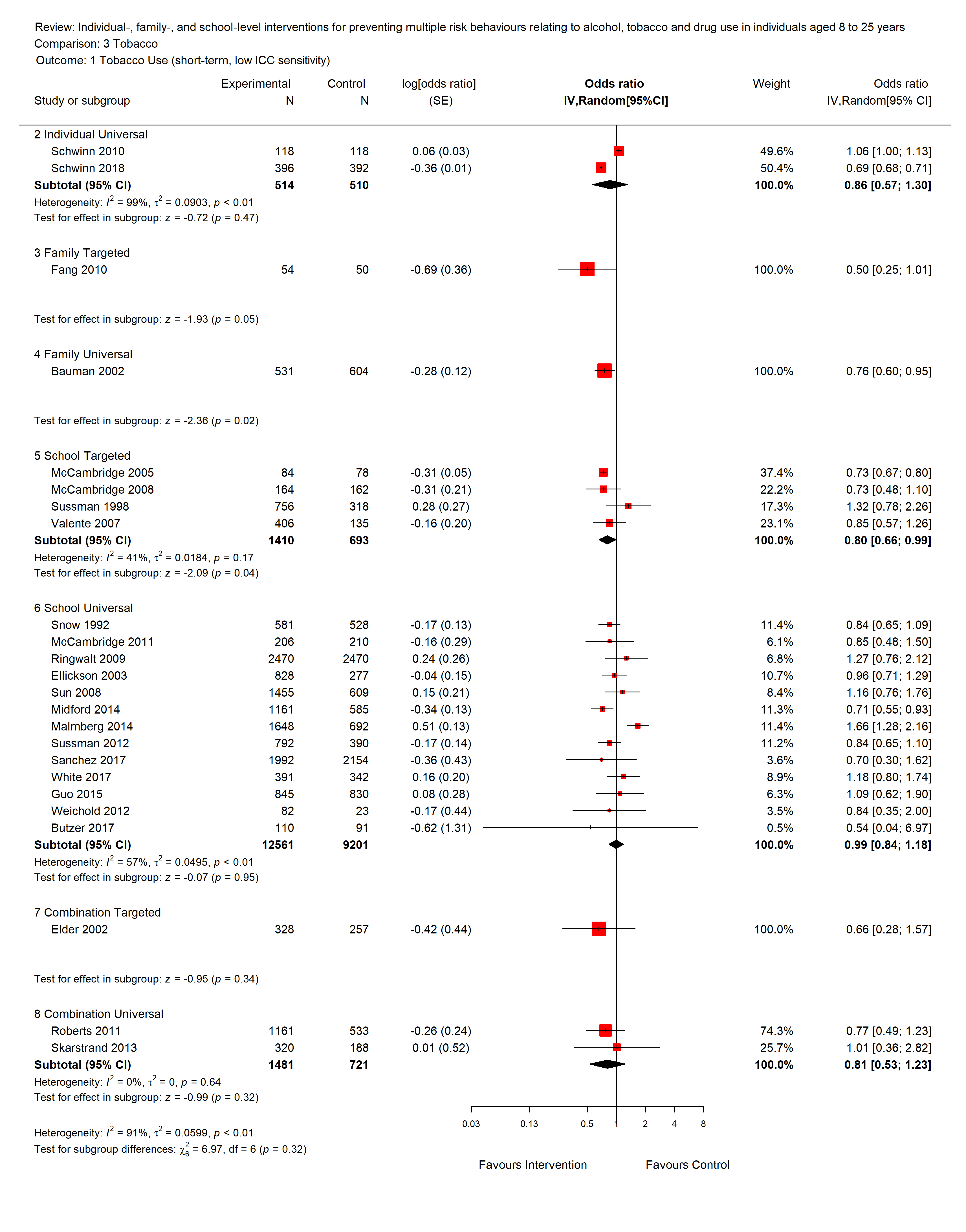


Additional Figure 8.10 short term tobacco use outcome with highest reported ICC


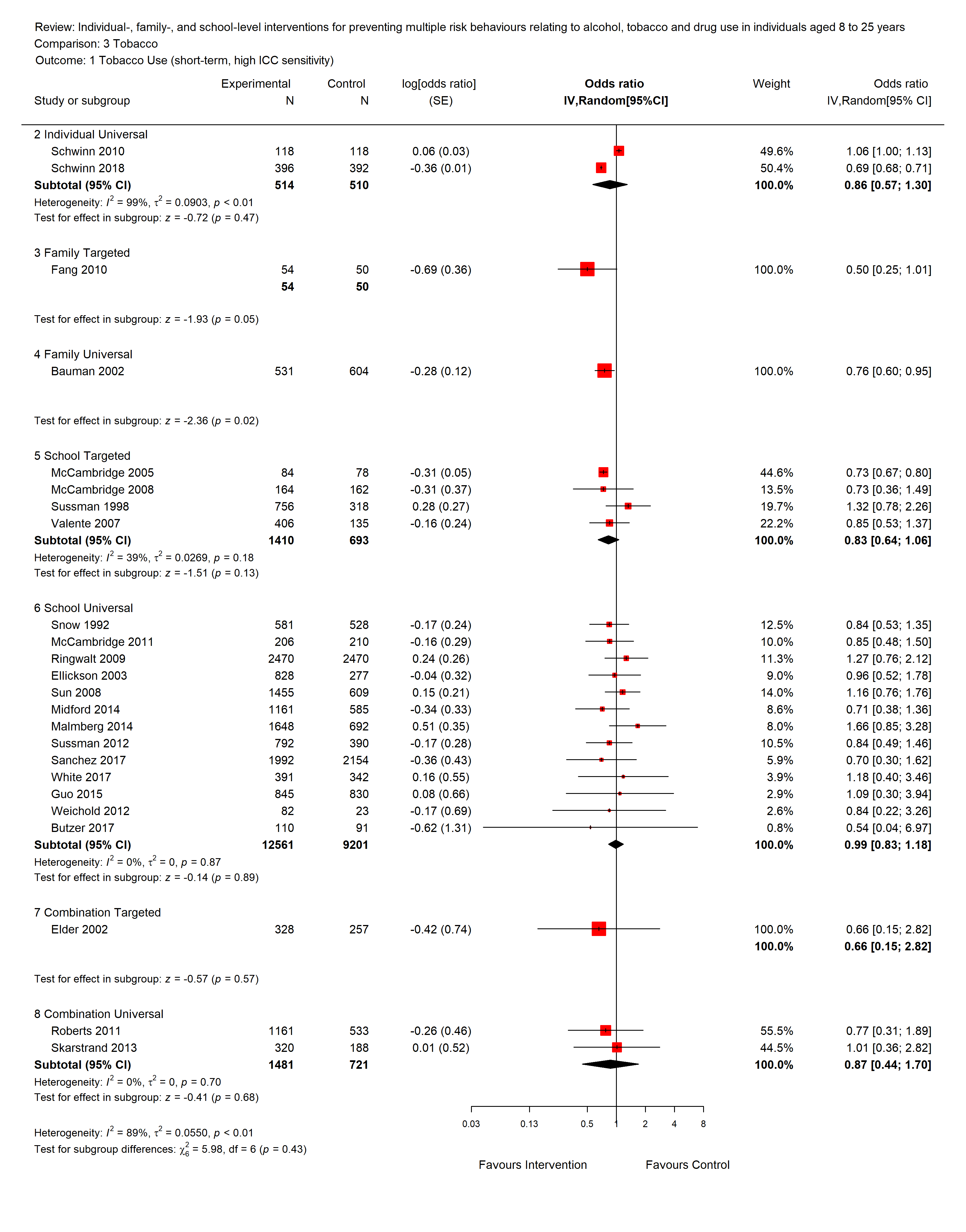


Additional Figure 8.11 long term tobacco use outcome with lowest reported ICC


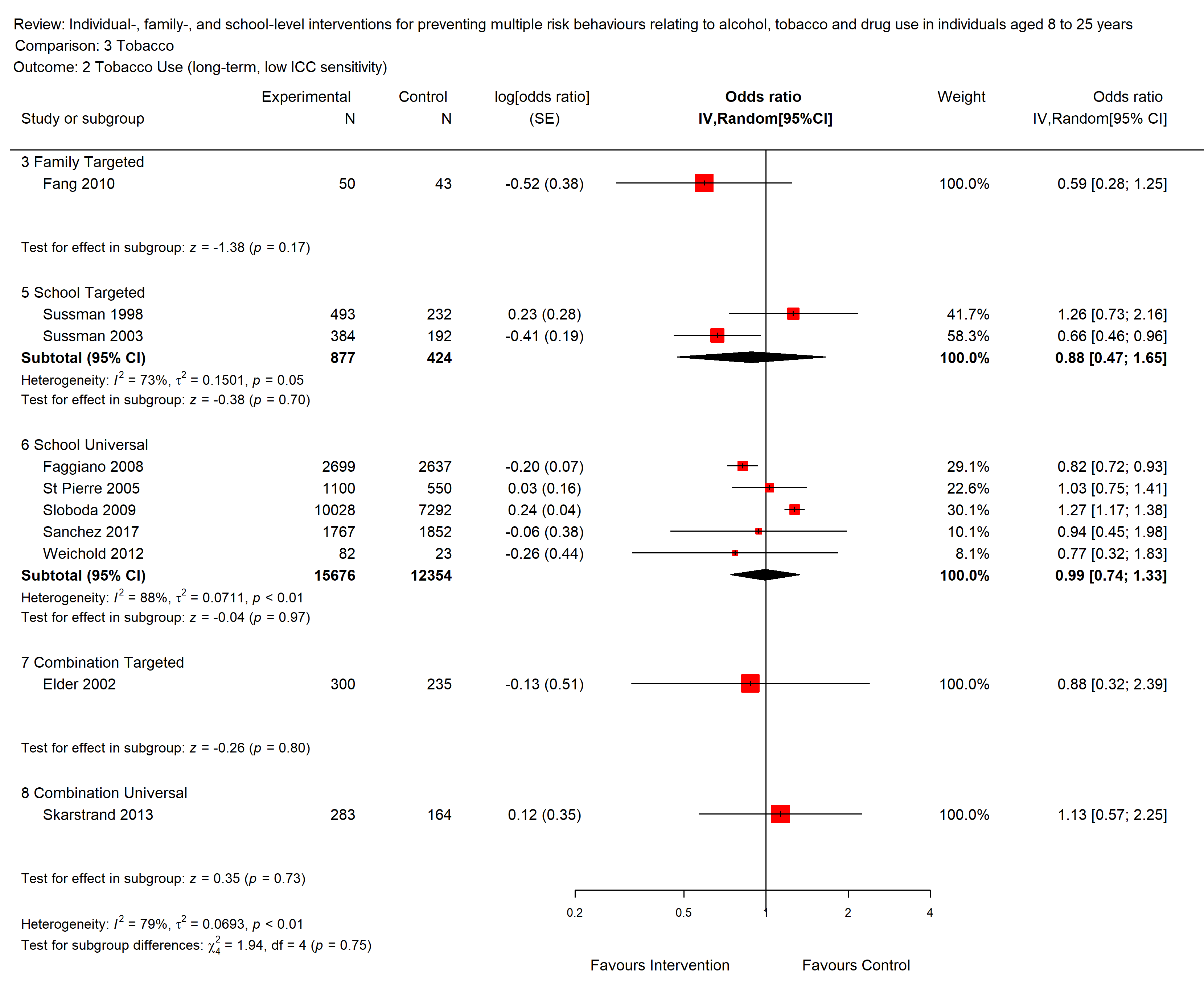


Additional Figure 8.12 long term tobacco use outcome with highest reported ICC


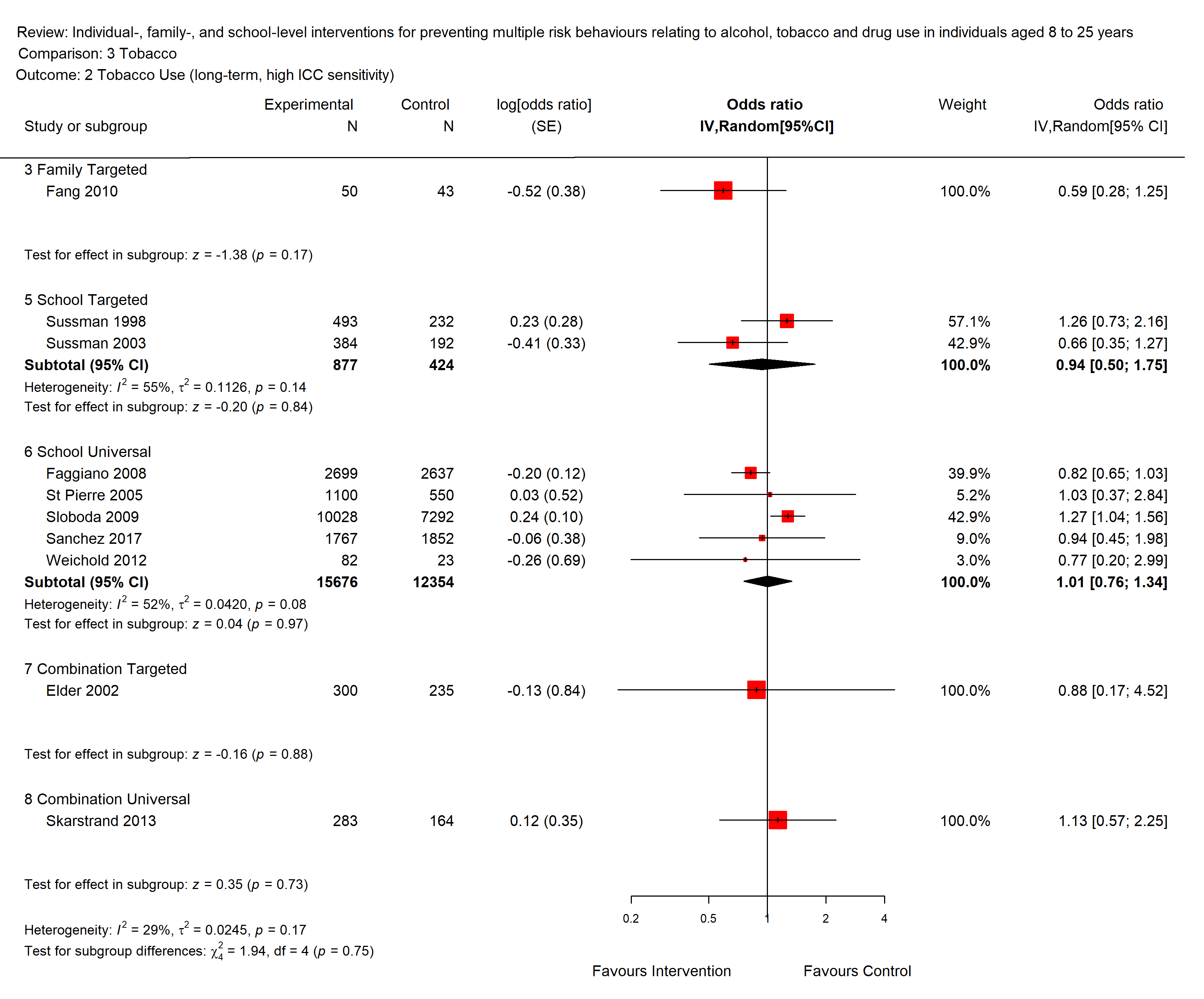


Additional Figure 8.13 short term illicit drug use outcome with lowest reported ICC


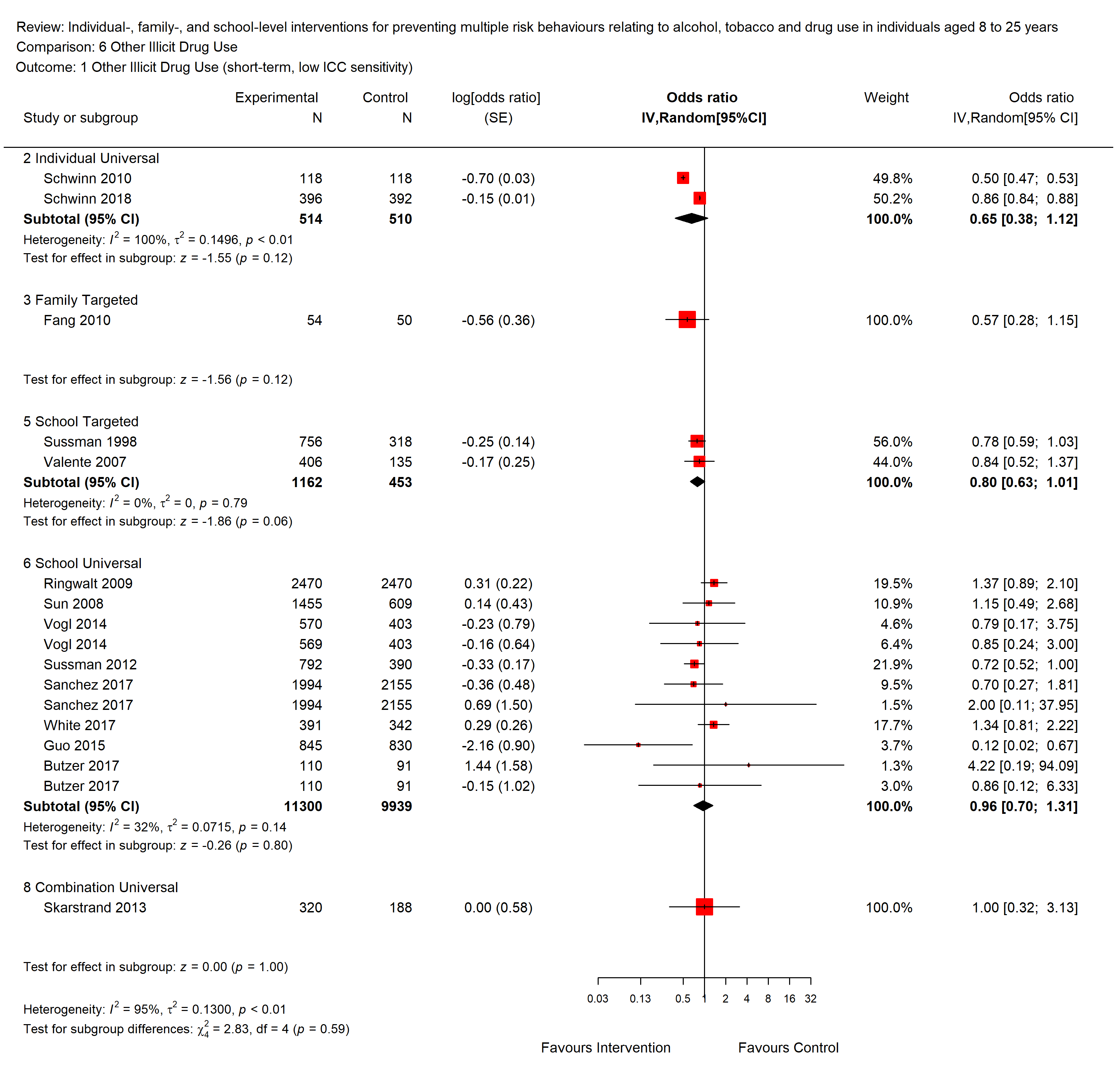


Additional Figure 8.14 short term illicit drug use outcome with highest reported ICC


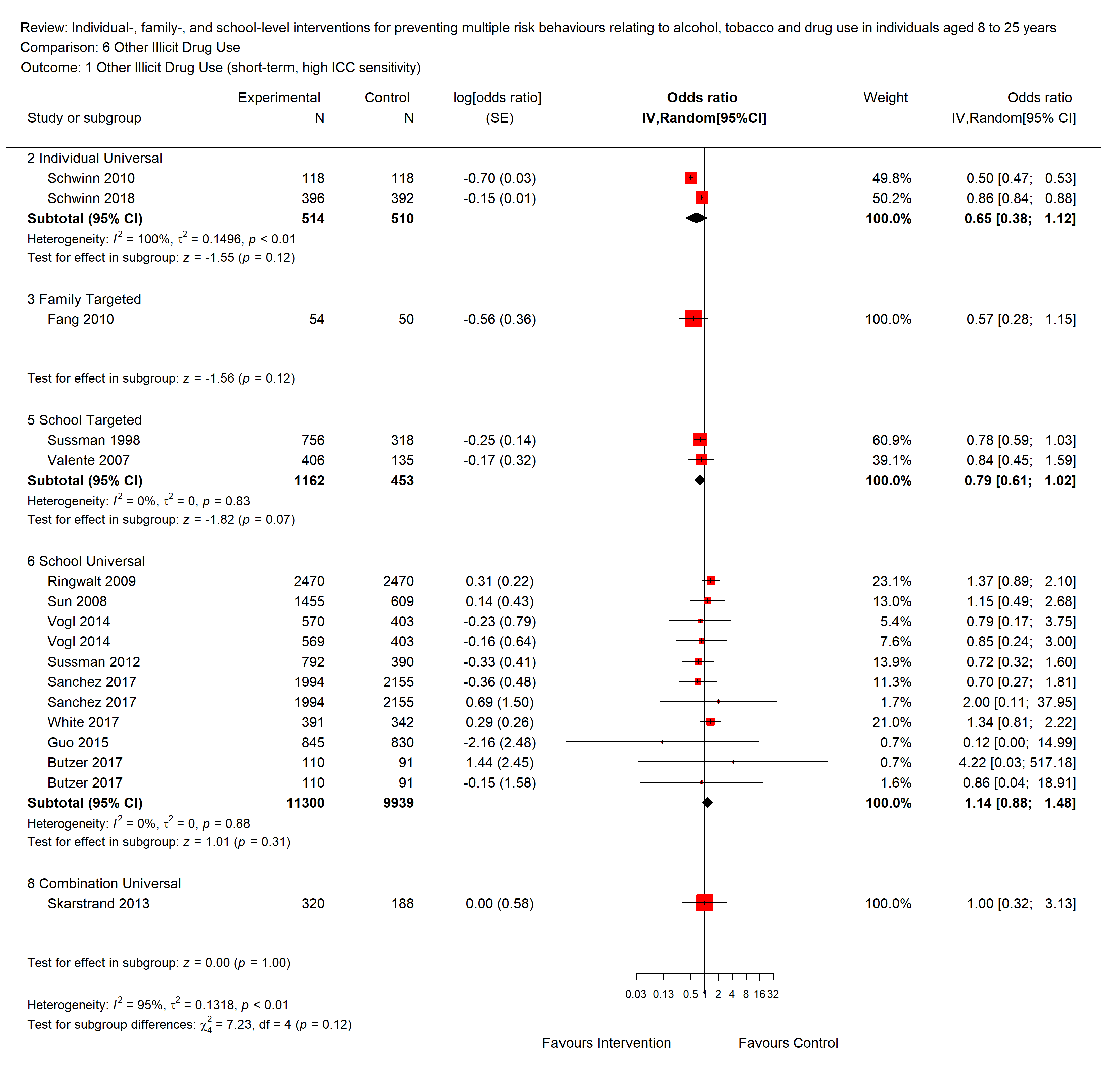


Additional Figure 8.15 long term illicit drug use outcome with lowest reported ICC


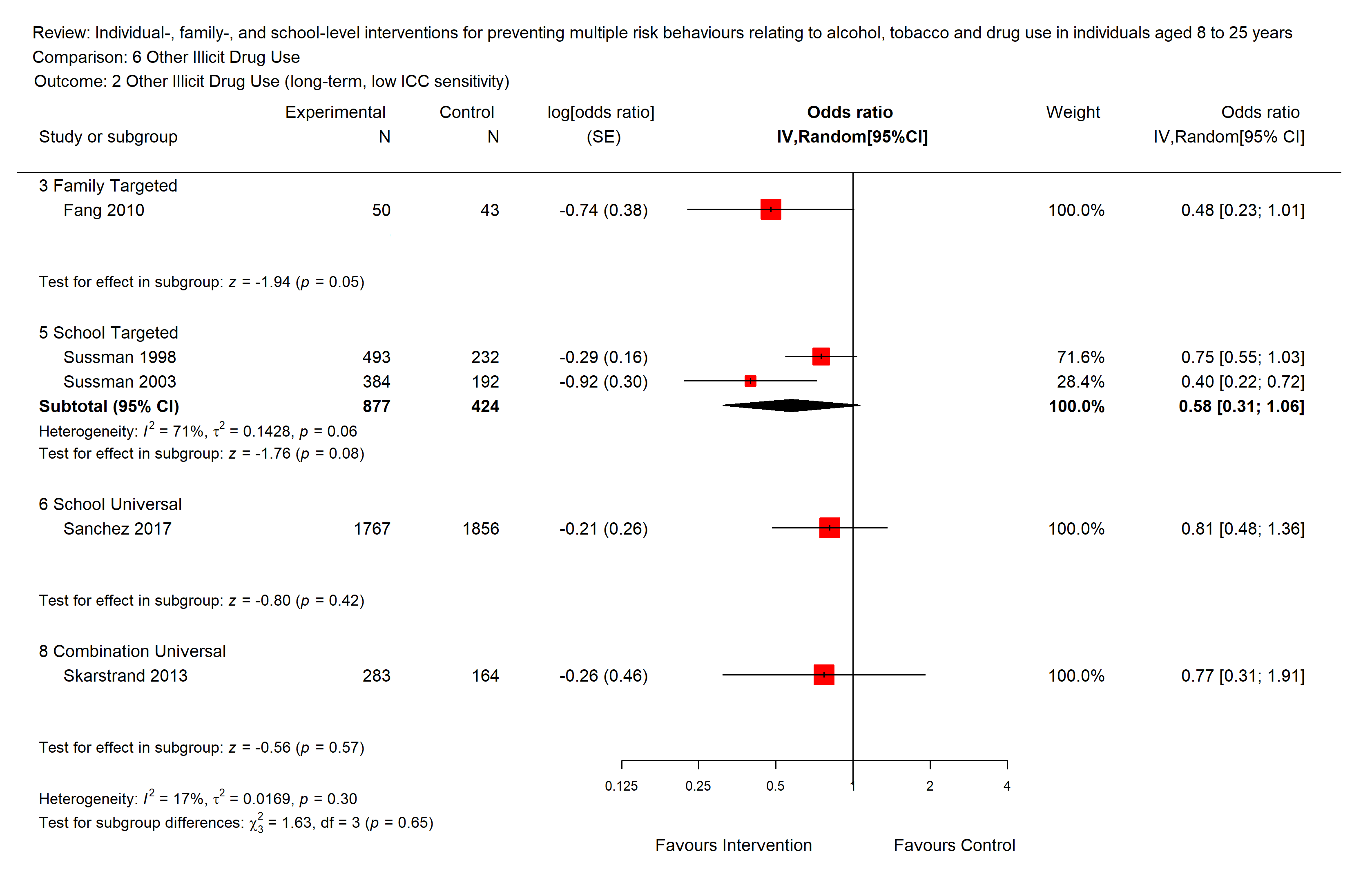


Additional Figure 8.16 long term illicit drug use outcome with highest reported ICC


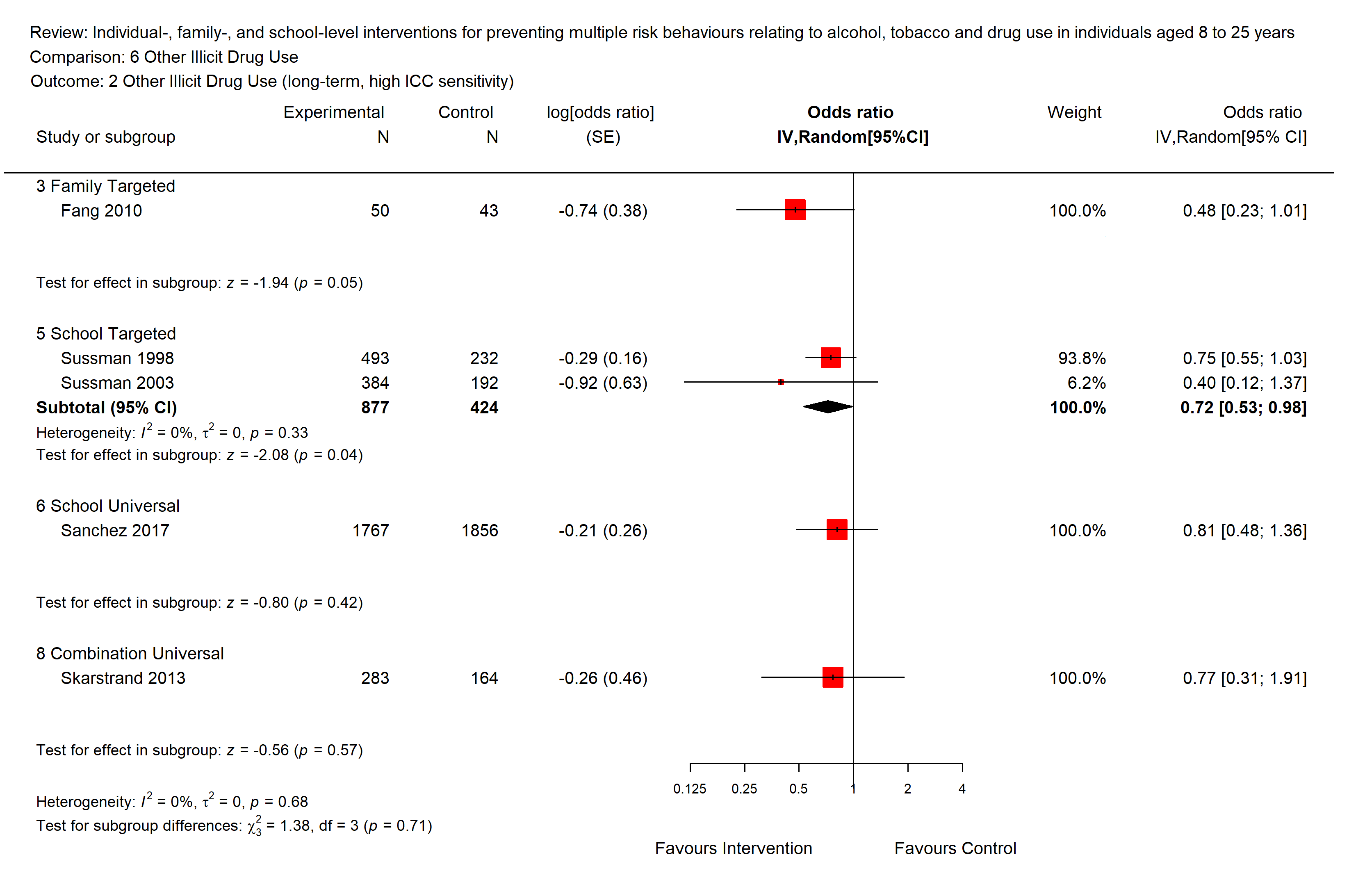


Additional Figure 8.17 long term cannabis use outcome with lowest reported ICC


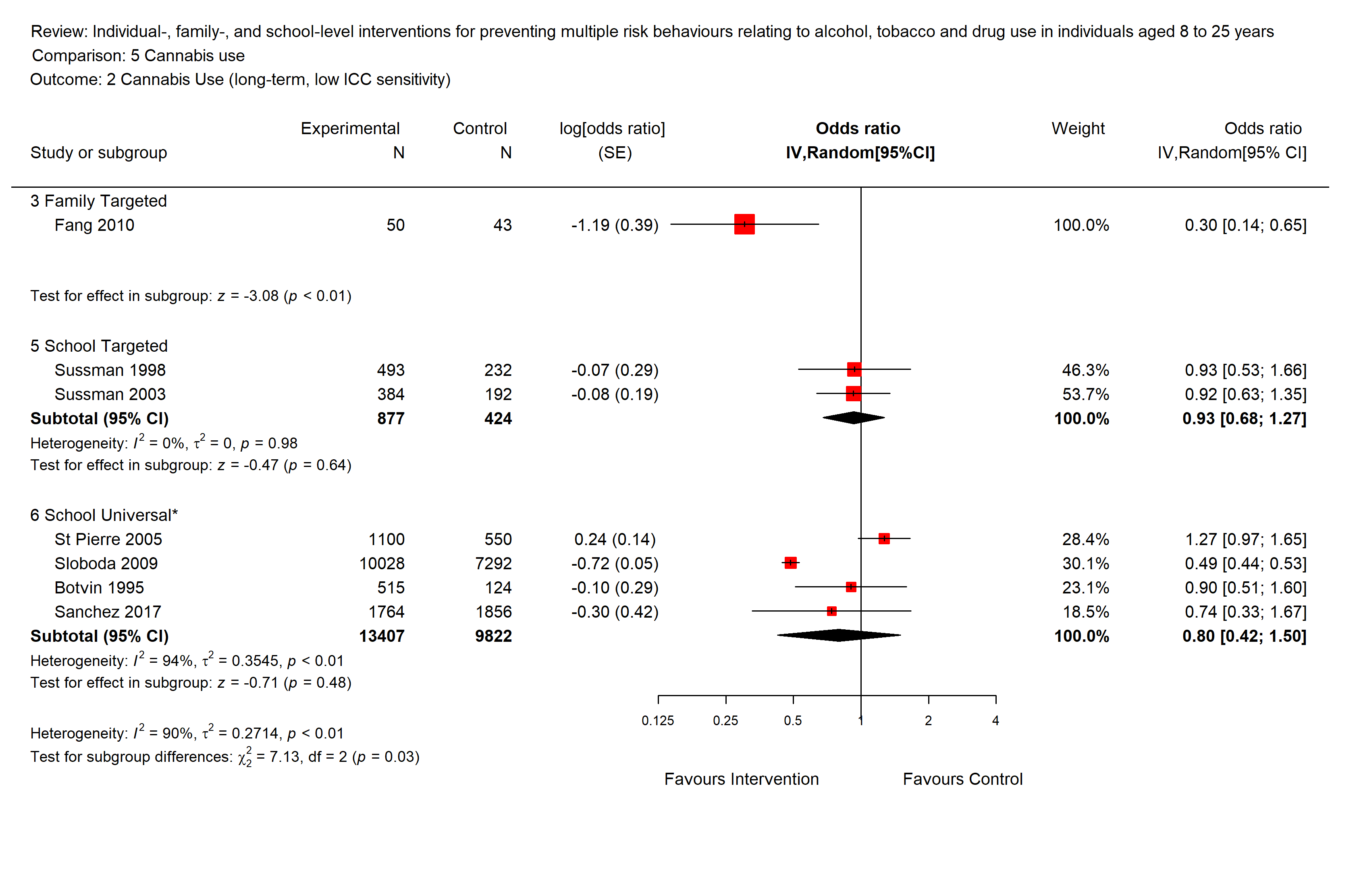


Additional Figure 8.18 long term cannabis use outcome with highest reported ICC


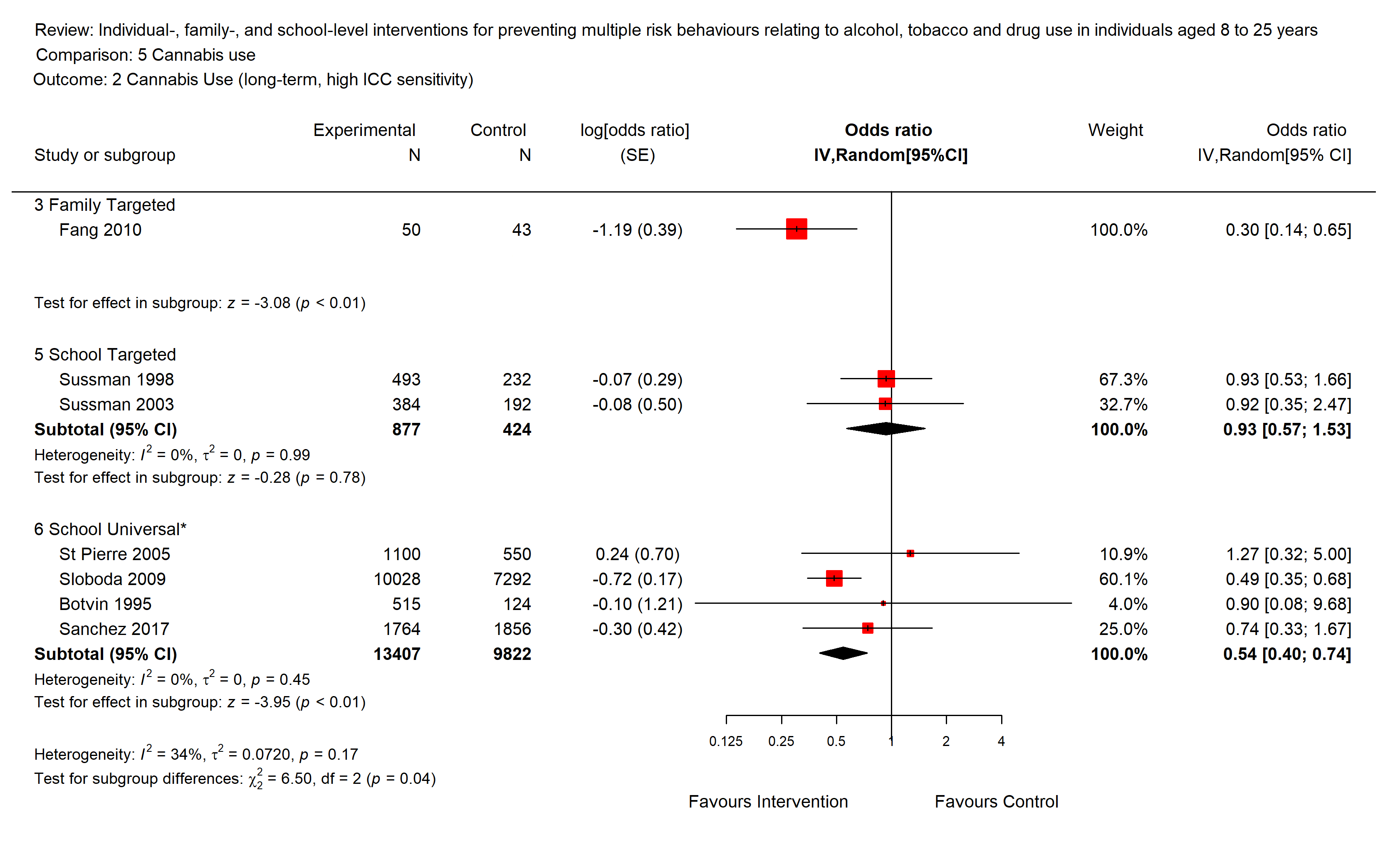


Additional Figure 8.19 short term cannabis use outcome with lowest reported ICC


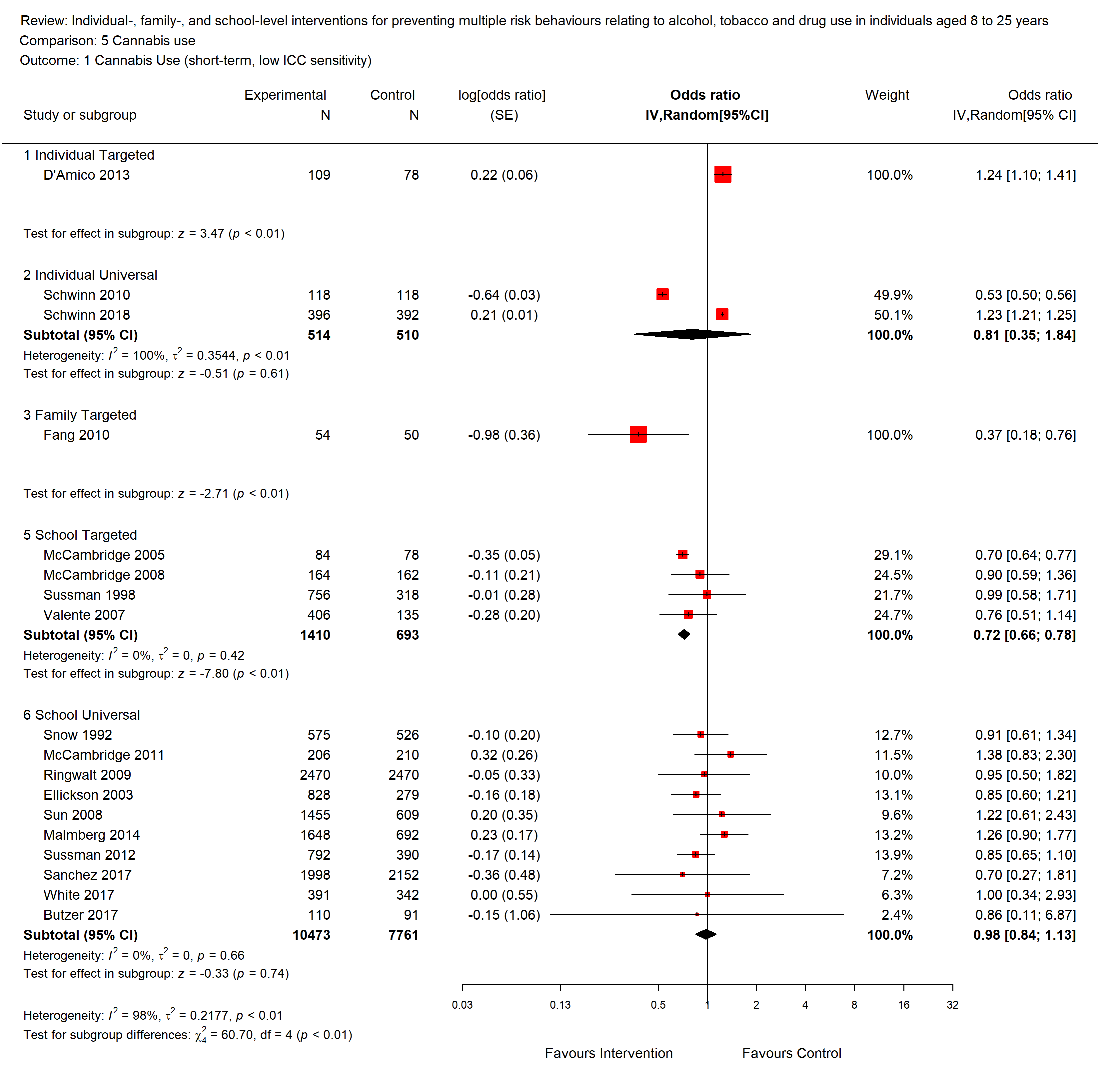


Additional Figure 8.20 short term cannabis use outcome with highest reported ICC


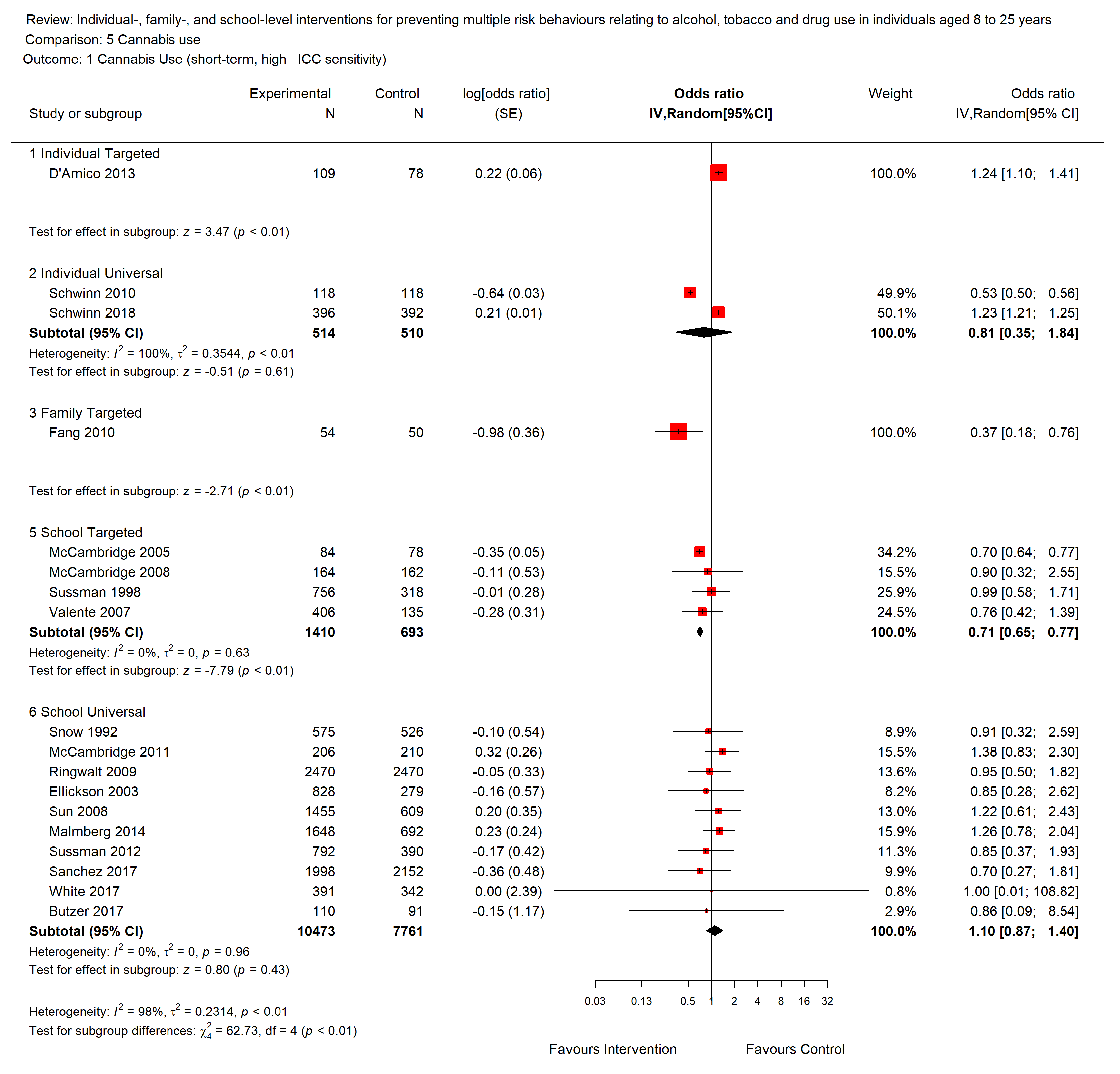


**Sensitivity analyses – fixed effects models**

Additional Figure 8.21 short term alcohol use outcome with fixed effects model


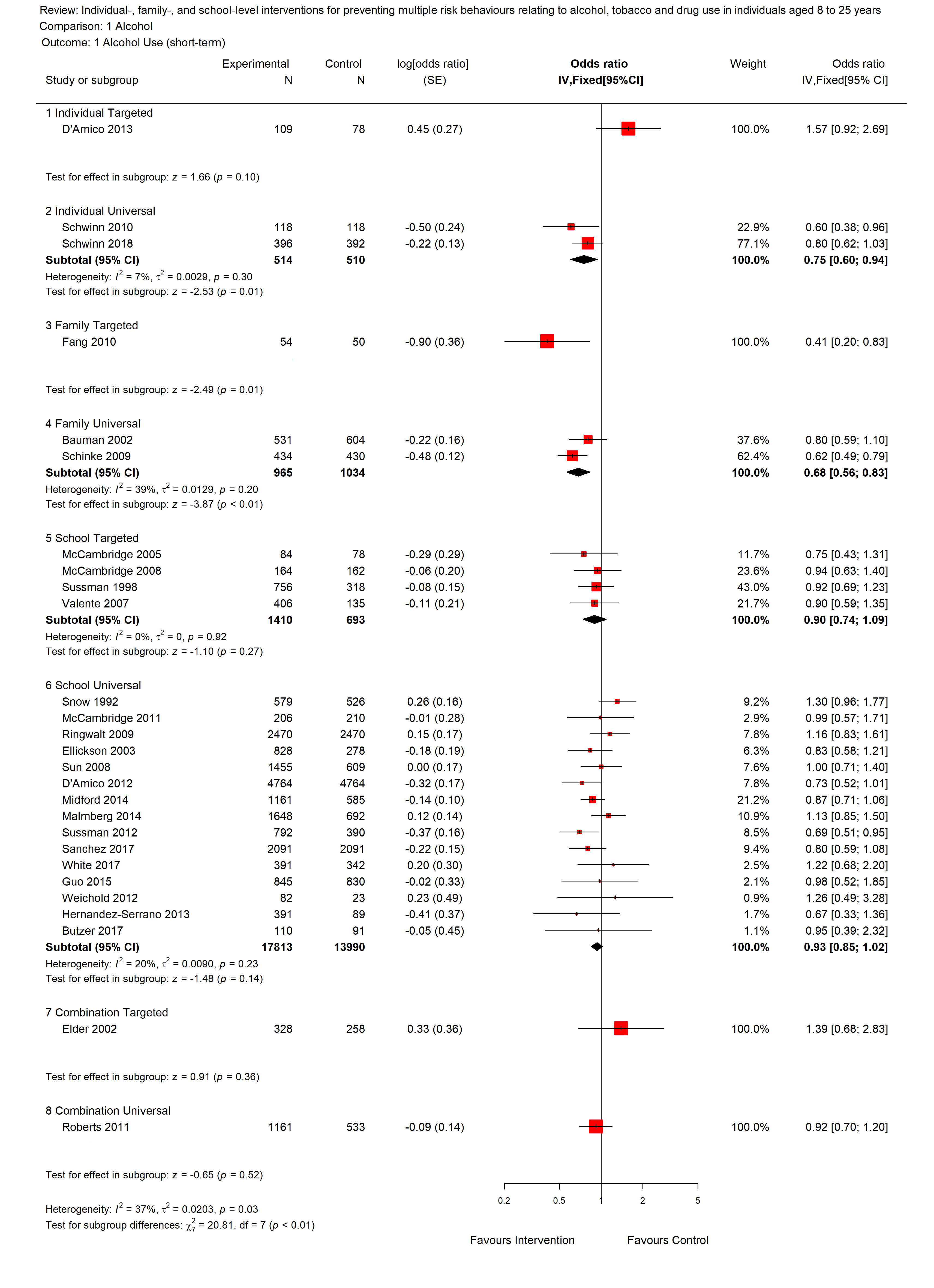


Additional Figure 8.22: Short term heavy alcohol use (binge drinking) outcome with fixed effects model


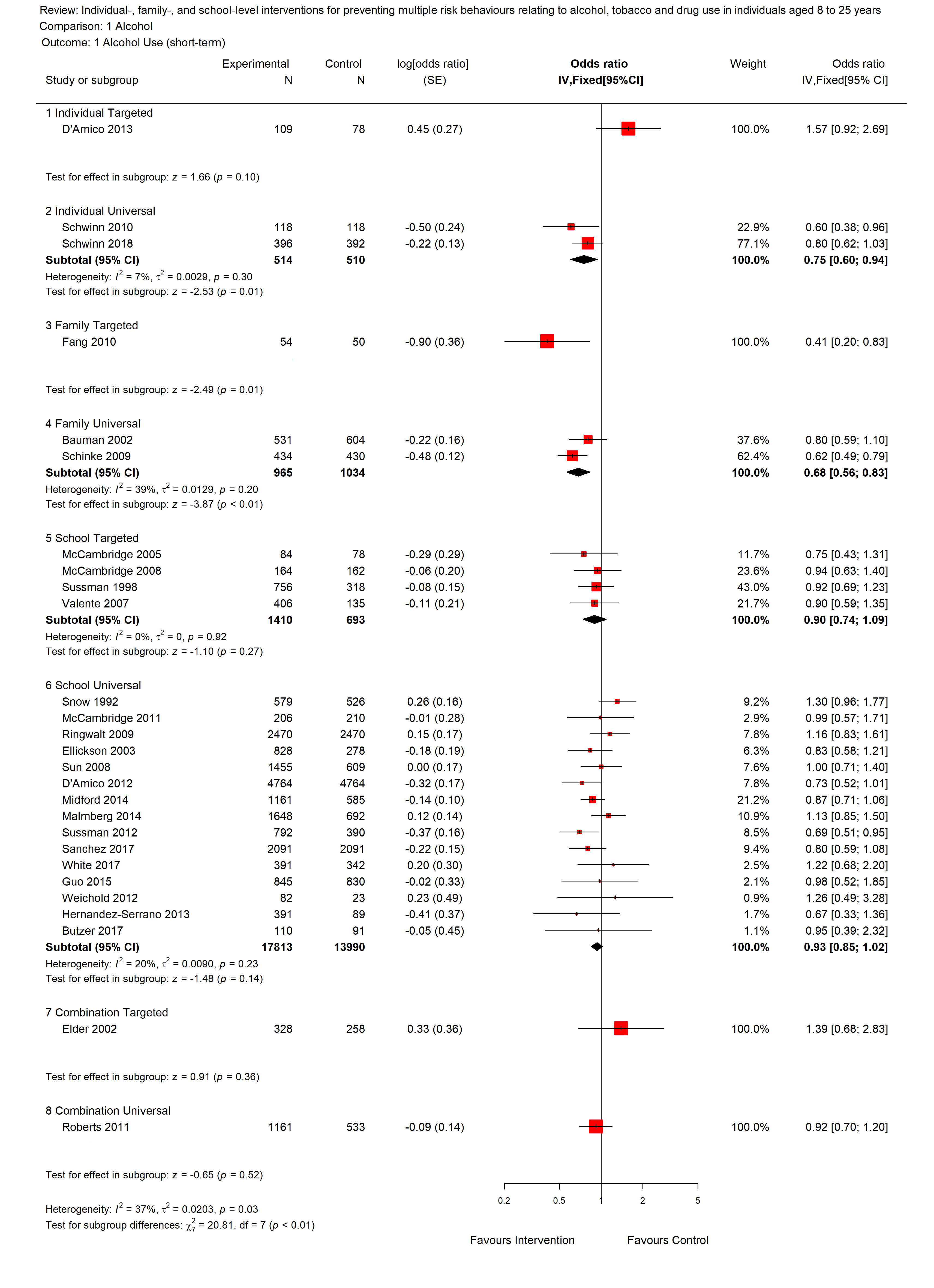


Additional Figure 8.23 short term tobacco use outcome with fixed effects model


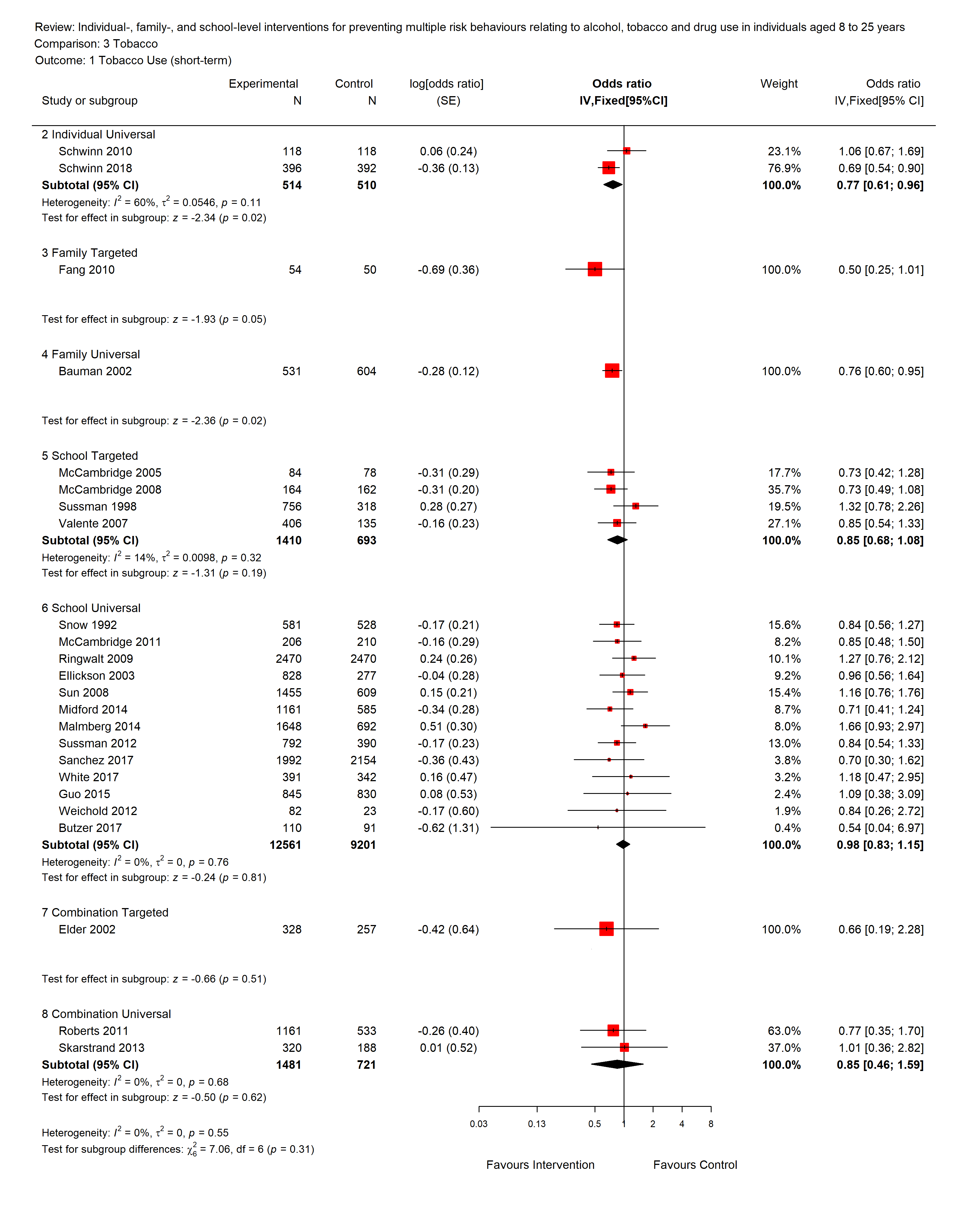


Additional Figure 8.24 short term cannabis use outcome with fixed effects model


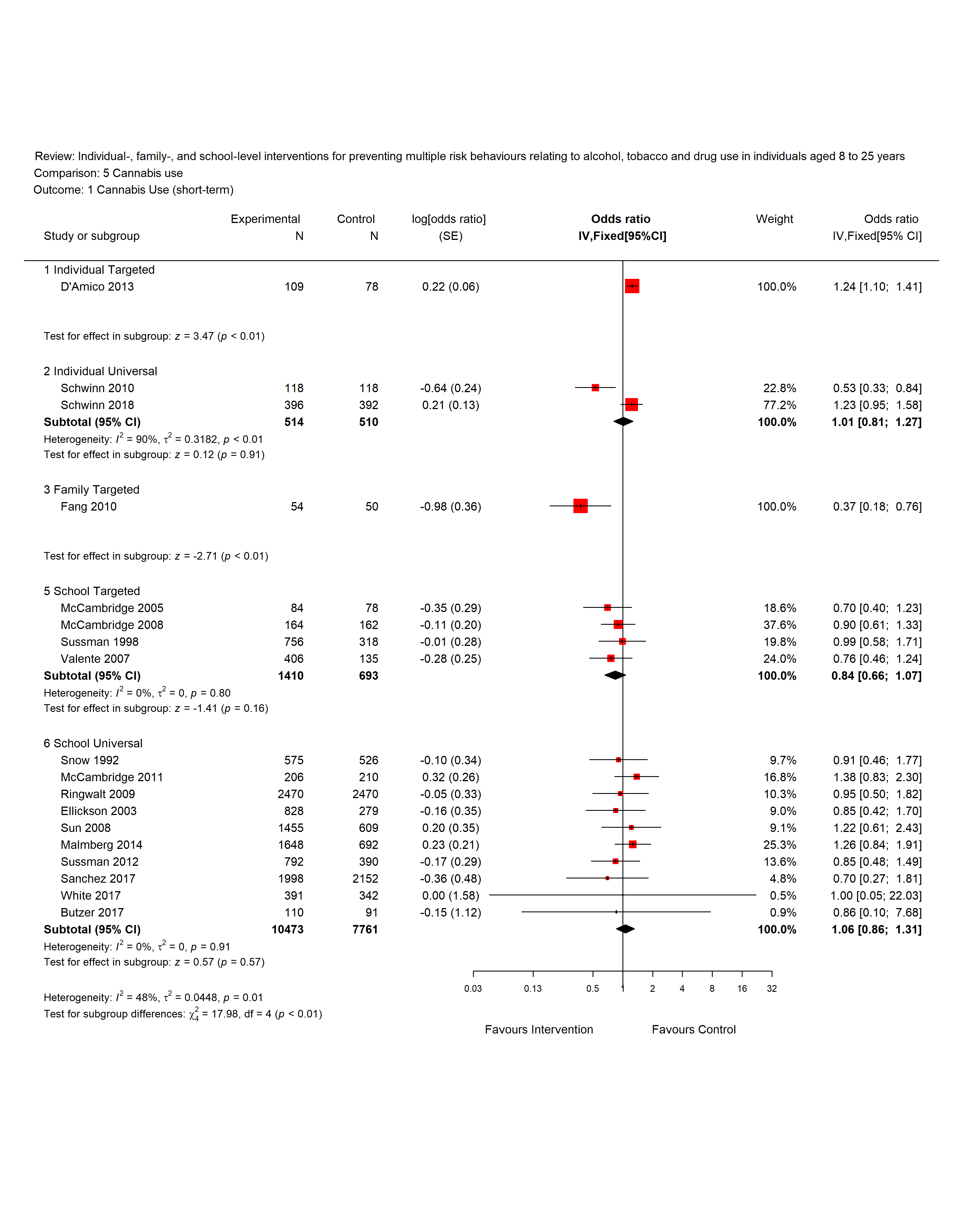
Additional Figure 8.25: Short term illicit drug use outcome with fixed effects model


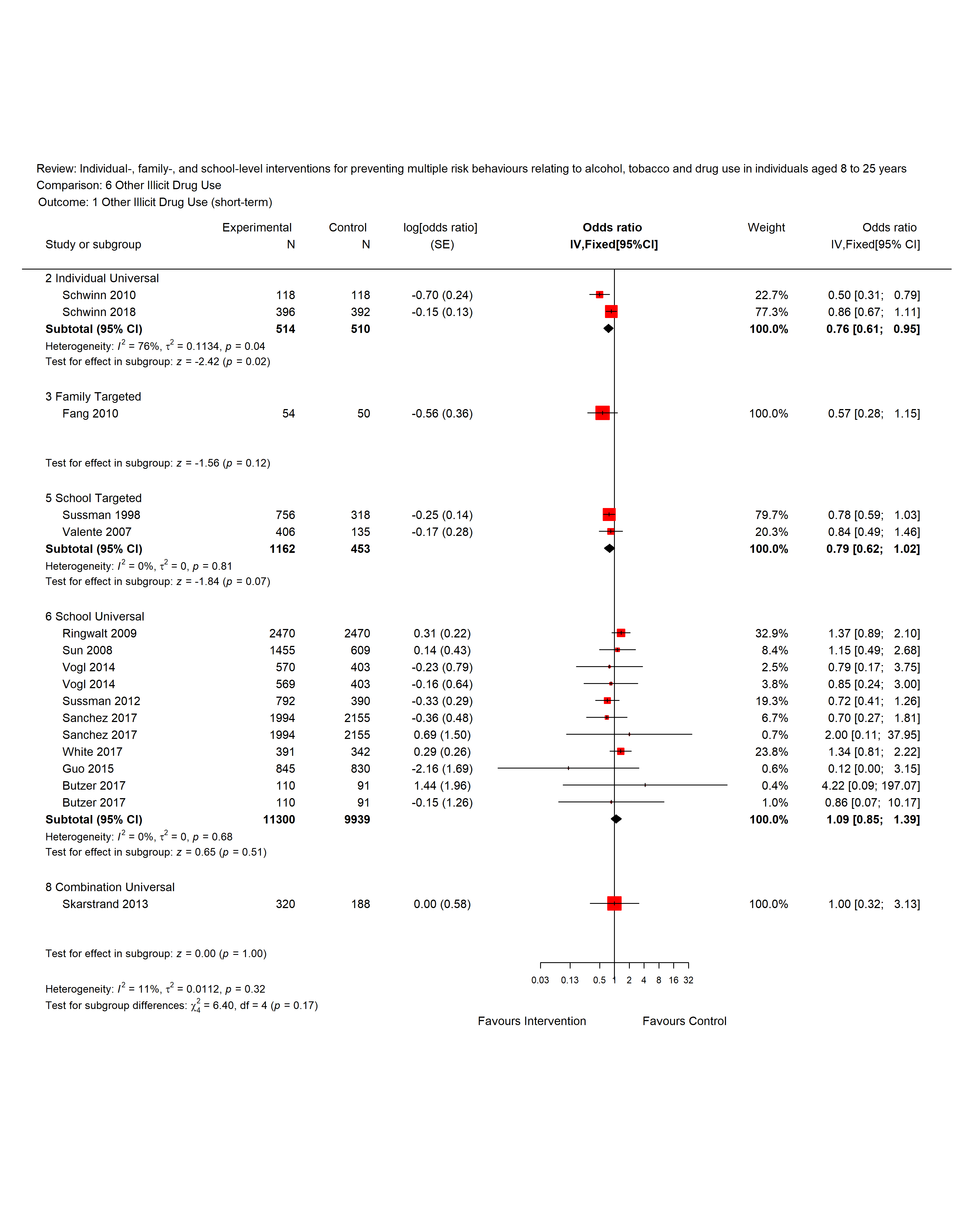

Supplement: Supplementary file 8 — Additional file 8. Sensitivity analyses. [file 12889_2022_13072_MOESM8_ESM.docx]
