# Supplementary material for: Individual-, family- and school-based interventions to prevent multiple risk behaviours relating to alcohol, tobacco and drug use in young people aged 8-25 years: a systematic review and meta-analysis
Source: BMC Public Health. 2022 Jun 3;22:1111. doi: 10.1186/s12889-022-13072-5 (PMC9165543; doi:10.1186/s12889-022-13072-5)

Additional File 9: Forest plots showing long term substance use outcomes

**
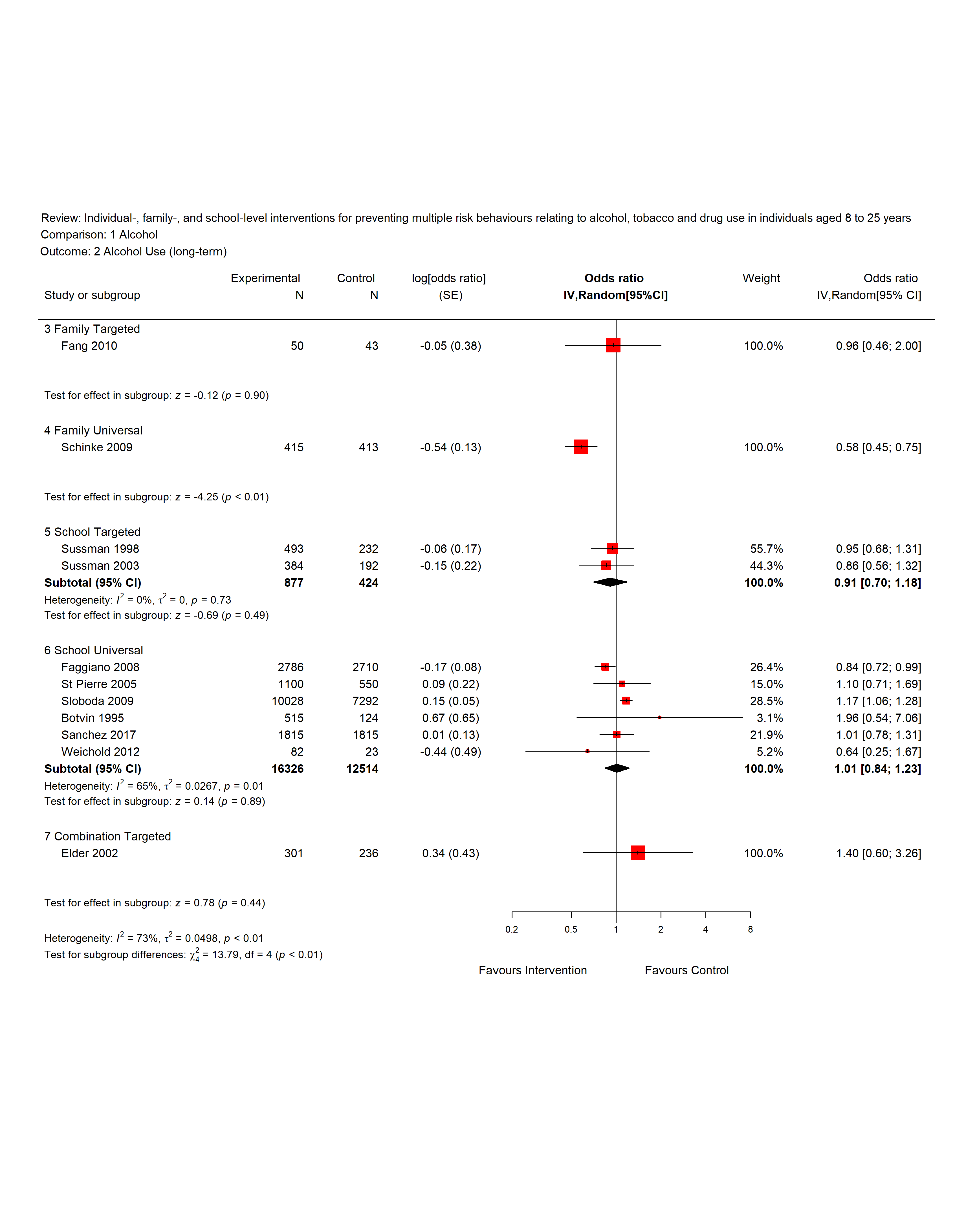
Figure 1: Alcohol use long term outcome**


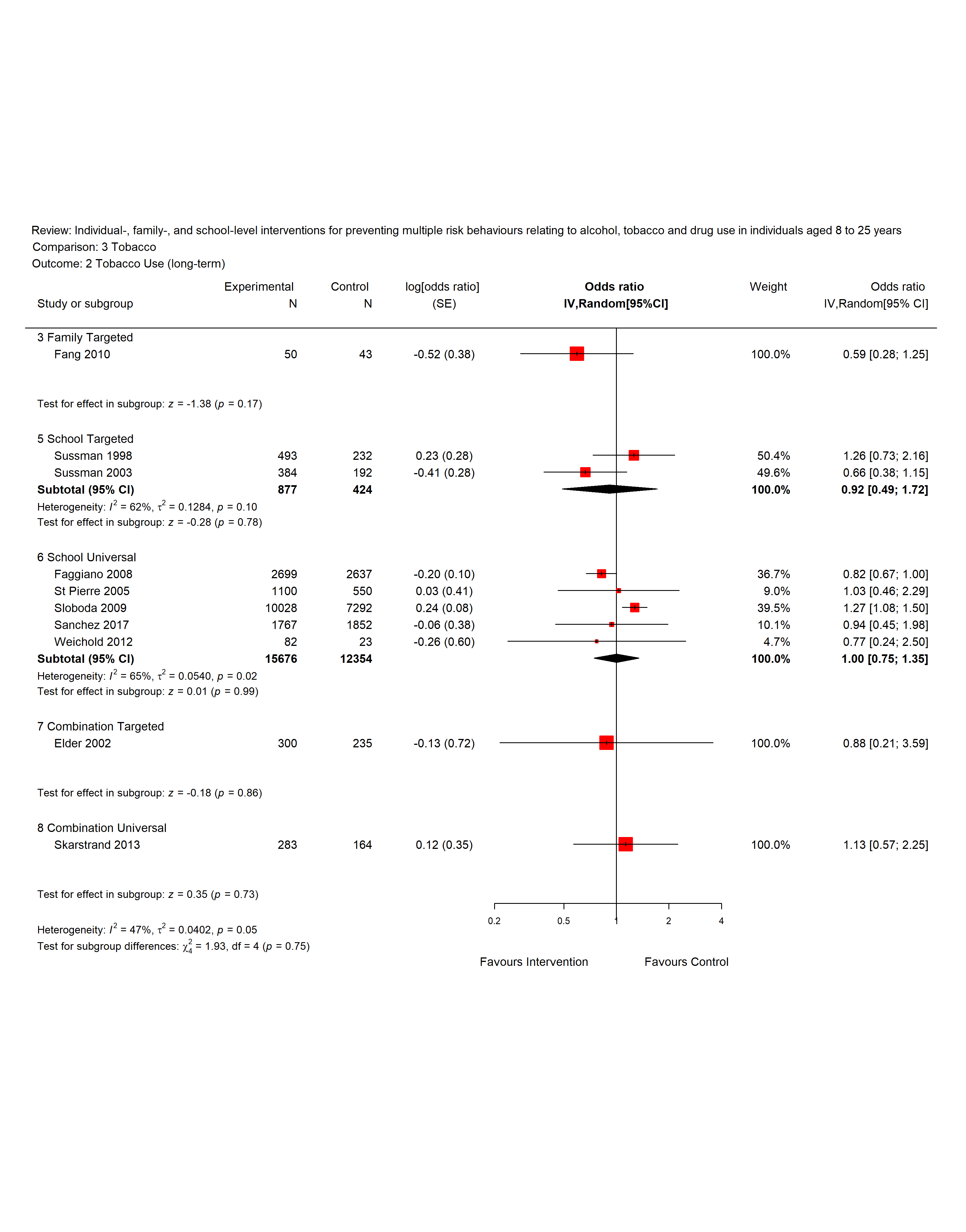


**Figure 2: Tobacco long-term use**


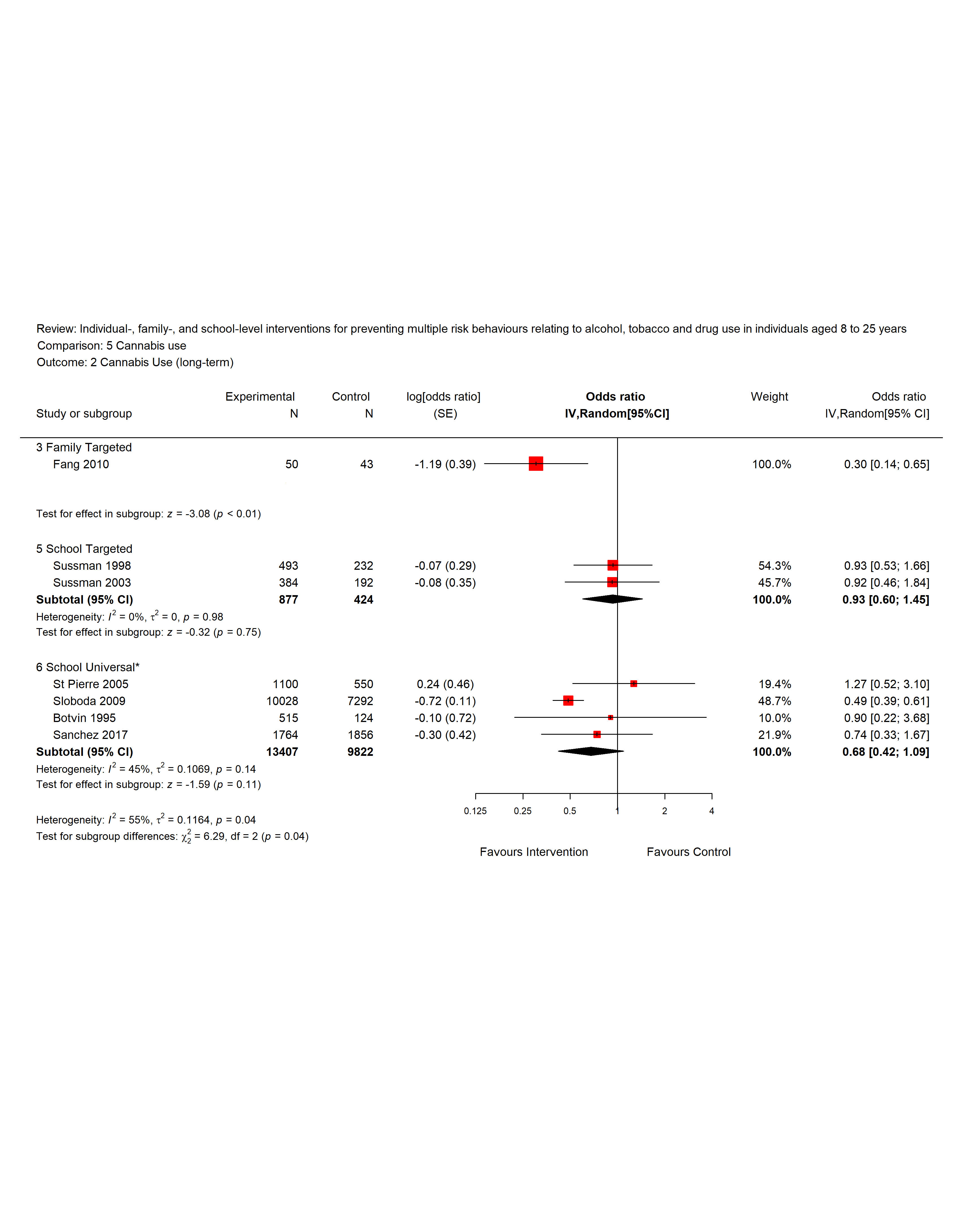


**Figure 3: Cannabis long-term use**


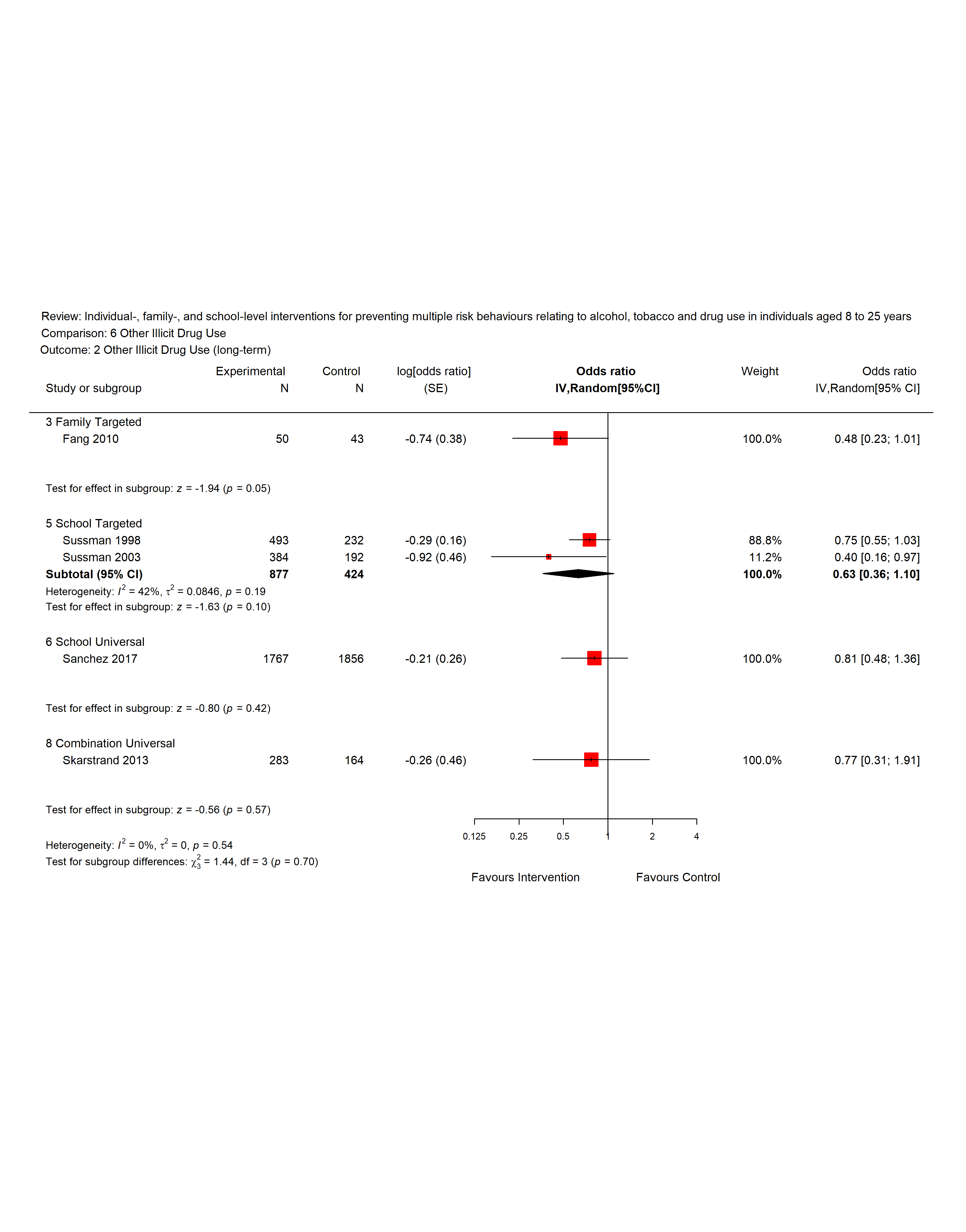


**Figure 4: Other illicit drug long-term use**

**Figure 5: Heavy alcohol (binge drinking) long term**


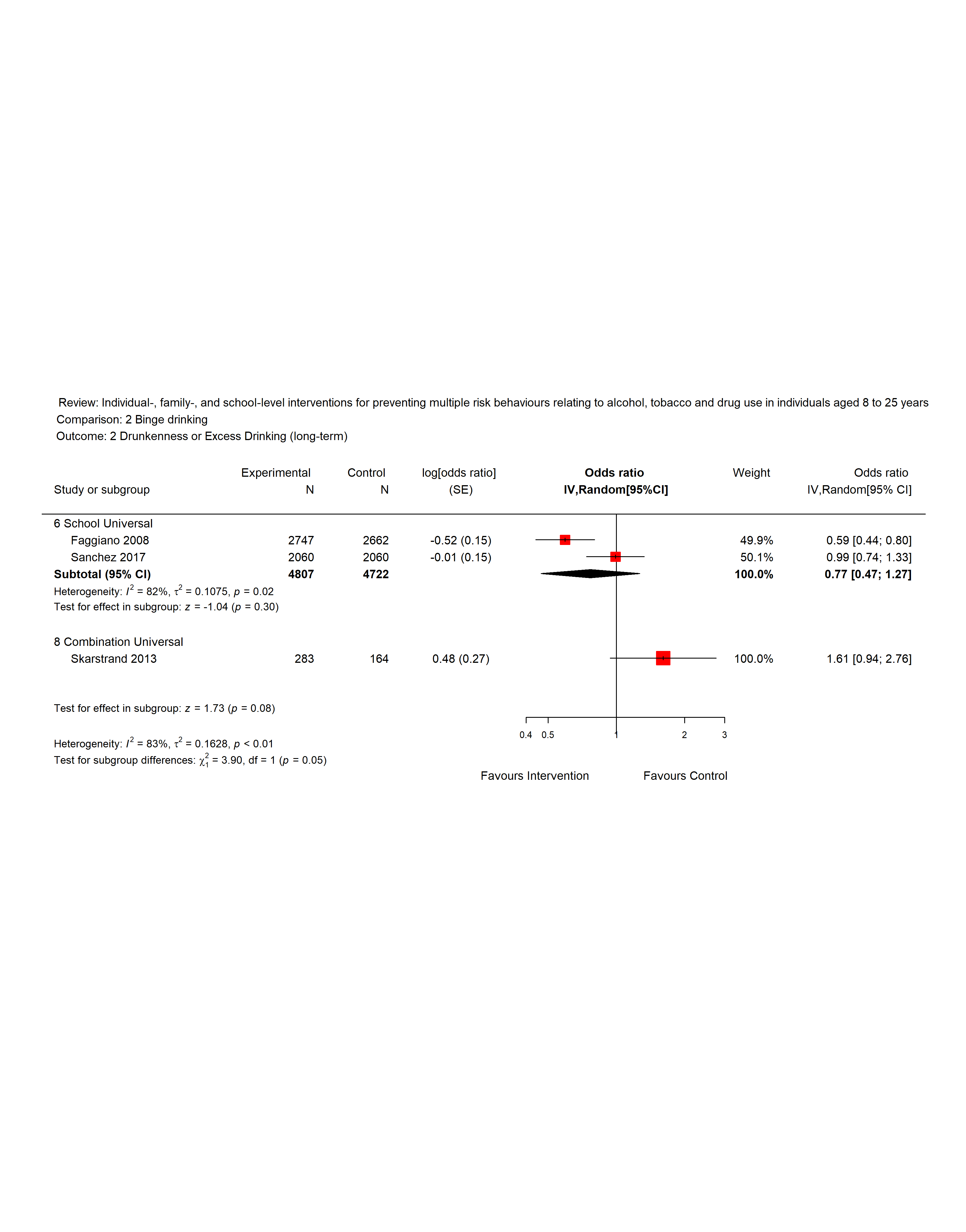

Supplement: Supplementary file 9 — Additional file 9. Forest plots for long term substance use outcomes. [file 12889_2022_13072_MOESM9_ESM.docx]
