# Supplementary material for: Individual-, family- and school-based interventions to prevent multiple risk behaviours relating to alcohol, tobacco and drug use in young people aged 8-25 years: a systematic review and meta-analysis
Source: BMC Public Health. 2022 Jun 3;22:1111. doi: 10.1186/s12889-022-13072-5 (PMC9165543; doi:10.1186/s12889-022-13072-5)

**Additional File 10: Funnel plots for assessing publication bias**

**Figure 10.1: Funnel plot for short term alcohol use outcome in school universal interventions**


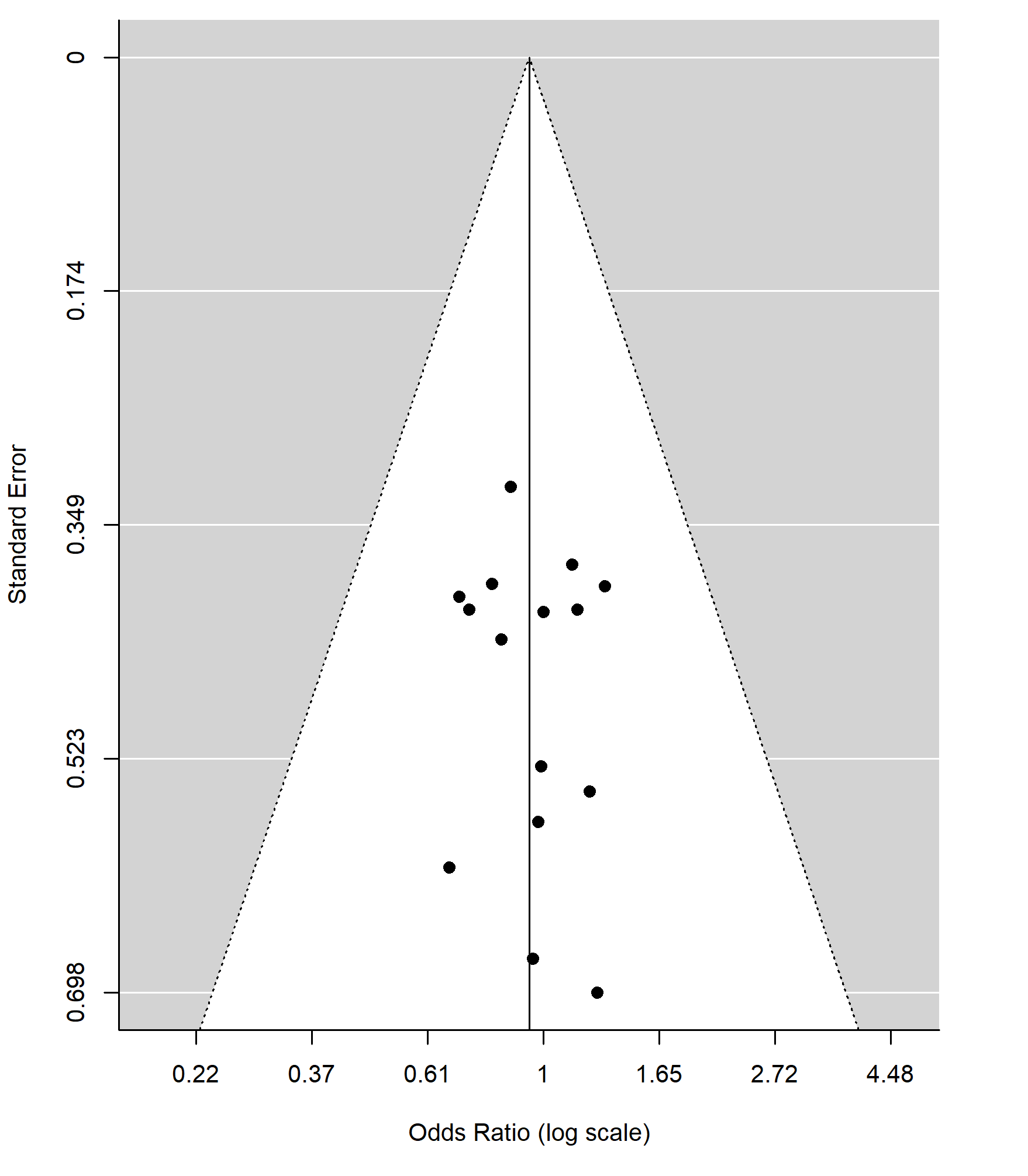


**Figure 9.2: Funnel plot for short term tobacco use outcome in school universal interventions**


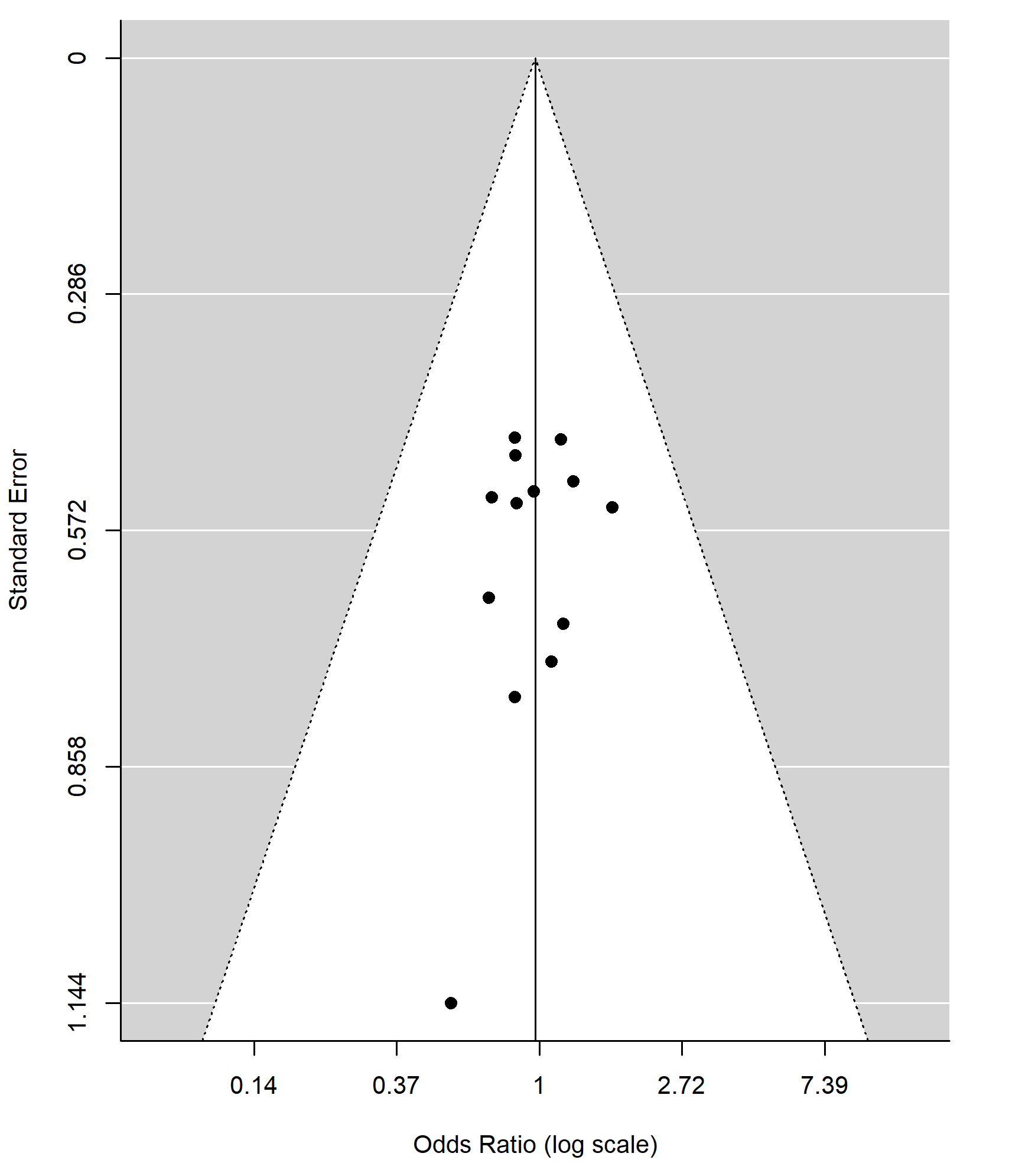


**Figure 9.3: Funnel plot for short term illicit drug use outcome in school universal interventions**


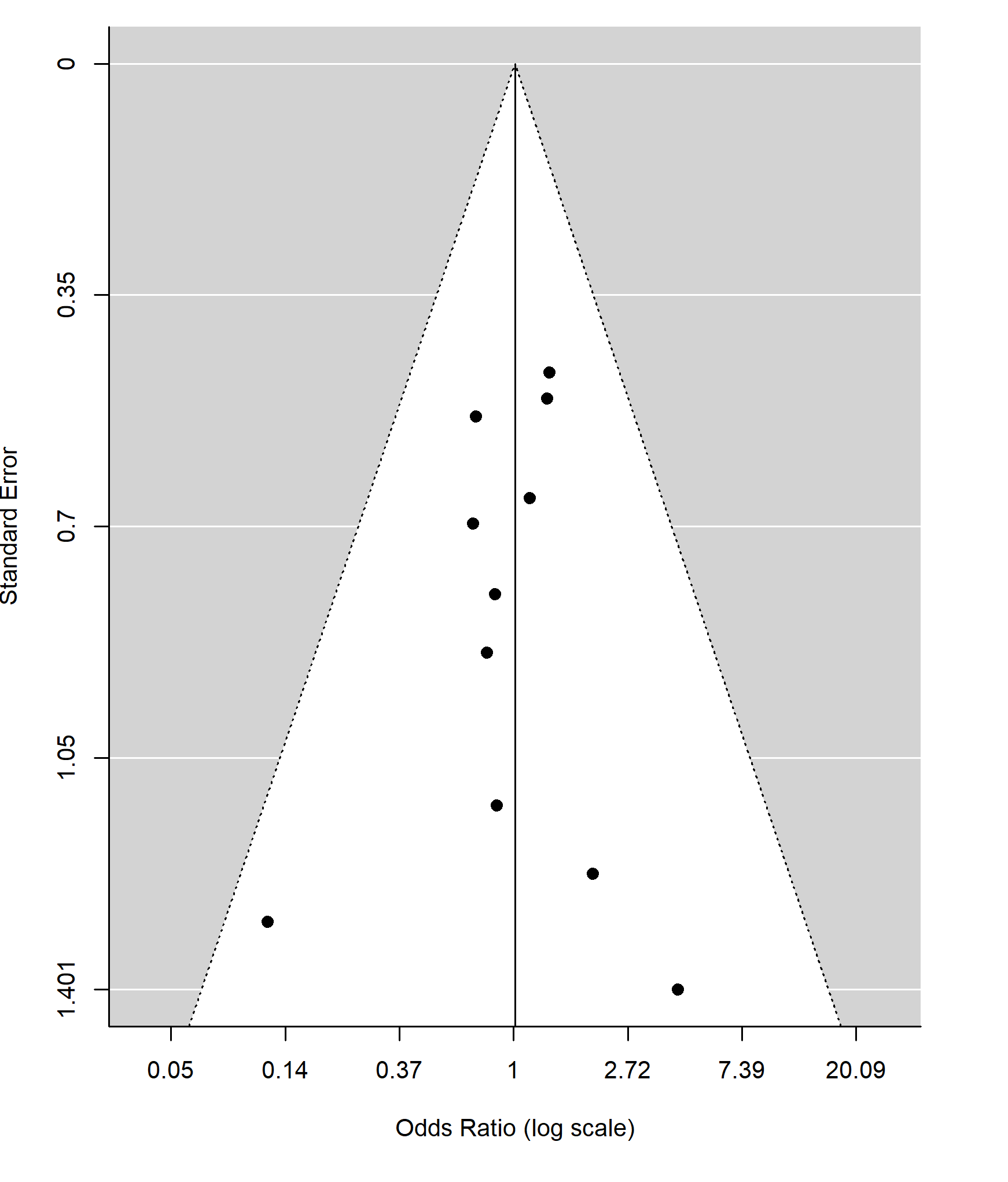

Supplement: Supplementary file 10 — Additional file 10. Funnel plots. [file 12889_2022_13072_MOESM10_ESM.docx]
