# Supplementary material for: Individual-, family- and school-based interventions to prevent multiple risk behaviours relating to alcohol, tobacco and drug use in young people aged 8-25 years: a systematic review and meta-analysis
Source: BMC Public Health. 2022 Jun 3;22:1111. doi: 10.1186/s12889-022-13072-5 (PMC9165543; doi:10.1186/s12889-022-13072-5)
Supplement: Supplementary file 11 — Additional file 11. Secondary outcomes. [file 12889_2022_13072_MOESM11_ESM.docx]

**Additional File 11: Secondary outcomes**

**Additional Table 11.1: Secondary outcomes measured by each study**

| **Trial name** | **Lead paper** | **Education, school exclusion, employment, benefit recipient** | **Crime record** | **Long term addictive behaviours** | **Health outcomes** | **Dependence/harmful substance use** | **Other** | **Adverse events on health/substance use of intervention** | **Cost effectiveness** |
| --- | --- | --- | --- | --- | --- | --- | --- | --- | --- |
| **2012-2018 searches** | | | | | | | | | |
| CHOICE | D'Amico 2012 |  |  | Intention to drink in the next six months; Resistance self-efficacy |  |  | Perceived alcohol use among peers at school | NR | NR |
| Climate schools (Vogl et al. 2014) | Vogl 2014 |  |  | Knowledge and attitudes (cannabis and psychostimulants) |  |  |  | NR | NR |
| DEVS | Midford 2014 |  |  | Knowledge, attitudes, communication with parents |  | Smoking harms experienced; Alcohol harms experienced | Lessons remembered; Alcohol consumption for risky drinkers and those who drank only | Control students found smoking lessons more memorable vs intervention - no other aversive outcomes reported | NR |
| Healthy school and drugs | Malmberg 2014 |  |  |  |  |  | Alcohol/tobacco/marijuana use among never users at baseline | NR | NR |
| Ho'ouna Pono | Okamoto 2016 | Engagement in community activities |  |  |  | Drug resistance strategies; perceived risk of drugs | Cultural affiliation | Favourable consequences of alcohol considered in decision to drink to greater extent in intervention group. | NR |
| Keepin' it real + FPNG | Marsiglia 2016 |  |  | Initiation |  | Anti-alcohol and anti-tobacco norms |  | Stated in Williams 2016 paper that "There were no harms or unintended effects." | NR |
| Project towards no Drug Abuse | Sussman 2012 |  |  |  | Risky sexual behaviour only baseline data given | Average number of different types of hard drugs in past 30d | Substance use index (sum of all substances in past 30d) | NR | NR |
| RealTeen | Schwinn 2018 |  |  |  |  |  |  | NR | Costs of recruitment (via Facebook ads), but not the intervention |
| Too Good for Drugs | Hall 2013 | FCAT Mathematics achievement; FCAT Reading achievement; Bond with prosocial peers |  | Intention to use; ATOD attitudes; Self-efficacy |  | Goals and decisions; Peer resistance; Harmful effects |  | NR | NR |
| Your decisions count | Evers 2012 |  |  |  |  | Use in last 30d in subgroup who had previously used at baseline |  | NR | NR |
| Tamojunto Drug Prevention Program | Sanchez 2017 |  |  |  |  |  | Grouped drug use | A potential iatrogenic effect on first alcohol use. Of all the results, the most concerning for the implementation of this program in schools as part of Brazilian public policy was the increase in RR of first alcohol use, suggesting an anticipation effect of alcohol use in these adolescents. This analysis suggests that the program has a persistent negative eﬀect for ﬁrst use. | NR |
| Life Skills + TPD Taiwan - Huang 2013 | Huang 2013 | Life skills |  | Intention to use; Subjective norms surrounding not using drugs; Perceived behavioural control; Attitudes towards not using drugs |  |  |  | NR | NR |
| Assist+FRANK | White 2017 |  |  |  | Health-related quality of life | Perceived prevalence of drug use in year group | Any drug offers in past year; Visited talk to FRANK website; Conversations with friends about drugs | "the pattern of results across proposed secondary outcomes (cannabis, smoking, glues, gases, aerosols, smoking, alcohol use) was mixed" | NR |
| Life Skills + TPD Taiwan - Guo 2015 | Guo 2015 |  |  | Attitudes towards illicit drug use; Norms concerning illicit drug use; Perceived behavioural control over illicit drug use; Intention to use illicit drug use; Life skills directed towards not using illicit drugs |  |  |  | NR | NR |
| IPSY | Weichold 2012 |  |  | Expectation to smoke in following year; Resistance skills (alcohol and smoking) |  |  |  | Against the expectations, the peer-led intervention seems to have an unexpected negative effect on the substance use behaviors of the students (in particular regarding alcohol use). In addition, students of the peer-led condition showed a higher increase in  expectations for regular smoking and lower resistance to cigarette offerings as compared to those of the teacher-led condition (but they did not exceed the normative development controls showed on these variables over time) | NR |
| SUPPER | Skeer 2016 |  |  |  |  |  | Conversations with parents about alcohol/smoking/drugs; Frequency and duration of parent/child meals | NR | NR |
| Saluda | Hernandez-Serrano 2013 | Social skills; Participation in leisure activities |  | Perceived norms and knowledge alcohol and illicit drugs; Intention to use substances |  |  |  | NR - although addition of leisure component actually resulted in less favourable effects on norms, knowledge and social skills. | NR |
| Family matters vs SFP | Byrnes 2012 |  |  |  |  | Decision making on drugs consumption; Attitudes towards personal and others consumption | Parent-child communication; Peer-pressure discussions | NR | NR |
| Programa Tu Decides | Calafat 1989 |  |  |  |  |  | Sources of information on drugs; People who students spend leisure time with; Activities in which students spend money; Leisure time use of the students | NR | NR |
| Programa Tu Decides | de la Rosa 1995 |  |  |  |  |  | Physical activity; Communication with parents; Money spent on drugs |  |  |
| Yoga | Butzer 2017 |  |  | Substance use willingness; Negative urgency; lack of premeditation; lack of perseverance; sensation seeking; positive urgency; future time perspective | Perceived stress; Mood (anger, confusion, depression, fatigue, vigour, tension) | Emotional self-control and self-regulation | Ever taken steroids or prescription drugs; present time perspective | Negative effects on time perspective outcomes for males (i.e. worsening) | NR |
| Free Talk | D'Amico 2013 | Delinquency |  |  | Number used alcohol or drugs before sex | Alcohol/Marijuana consequences |  | NR - although it does rather look like intervention associated with increased substance use (though effects very small) | NR |
| Swedish SFP | Skarstrand 2013 |  |  | Norm-breaking behaviours lifetime |  |  |  | Reported in results sections that at some time points males in the intervention group are reported to engage in substance use to a greater extent. However in discussion this is not portrayed. | NR |
| **Up to 2012 searches** |  |  |  |  |  |  |  |  |  |
| AAPT Donaldson | Donaldson 1995 |  |  | Resistance Skills |  |  | Prevalence of Offers. | "This suggests that resistance training by itself may lead adolescents to believe that drug use among their peers is prevalent" - Donaldson 1994. "The potentially harmful effect examined in this research wasthat resistance-skills training increases estimates of alcohol, cigarette and marijuana offers...for adolescents attending public school but not for those attending private Catholic school" - Donaldson 1995. | NR |
| Drug education course | Schaps 1982 |  |  |  |  |  |  | Unclear | NR |
| Gersick | Snow 1992 |  |  |  |  |  |  | Unclear - unintended outcome that intervention students showed greater experimentation with alcohol than control students | NR |
| Aussie optimism | Roberts 2011 |  |  |  |  |  |  | Unclear | NR |
| ASAP | Sloboda 2009 |  |  |  |  |  |  | Negative intervention effects for alcohol and cigarette use which significantly occurred among nonusers at baseline, females and white students | NR |
| Assertion training | Horan 1982 |  |  |  |  |  |  | Unclear | NR |
| Climate schools | Newton 2009 | Moral disengagement; Truancy |  | Cannabis harms (knowledge); Alcohol harms (knowledge) | Psychological distress |  |  | Unclear | NR |
| DARE | Clayton 1996 |  |  |  |  |  |  | Unclear | NR |
| ALERT Bell | Bell 1993 |  |  |  |  |  |  | Unclear | NR |
| ALERT + | Ellickson 2003 |  |  | normative perceptions, self-efficacy | Risky Sexual Behaviour | Consequences of use (beliefs about); Risk of dependency |  | Unclear | NR |
| ALERT Ringwalt | Ringwalt 2009 |  |  |  |  |  |  | "No adverse events or negative side effects from the program were reported" Ringwalt 2009 p627 | NR |
| ALERT St Pierre | St Pierre 2005 |  |  |  |  |  |  | NR | NR |
| EU_DAP | Faggiano 2008 |  |  | Attitudes, refusal skills, knowledge and perceptions of friends use |  |  |  | NR | "The low cost of the implementation of the program, estimated in 200 € per class, including teachers' training and materials, suggests a cost-effective investment." Faggiano 2008 p542 |
| Keepin it real | Hecht 2003 |  |  |  |  |  |  | NR | NR |
| Keepin it real plus | Hecht 2008 |  |  |  |  |  |  | NR | NR |
| Lions quest | Eisen 2002 |  |  |  |  |  |  | NR | NR |
| Migrant education | Elder 2002 |  |  | Susceptibility to drinking and smoking |  |  |  | NR | NR |
| Midwestern prevention project IND | Rohrbach 1994 |  |  |  |  |  |  | NR | NR |
| Mother-Daughter - Asian-American | Fang 2010 |  |  |  | Depression |  |  | NR | NR |
| Mother-Daughter - Non-Specific Population | Schinke 2009 |  |  |  | Depression |  | Mothers weekly alcohol use | NR | NR |
| Motivational interviewing 2005 | McCambridge 2005 |  |  |  |  | Overall AUDIT score |  | NR | NR |
| Motivational interviewing 2008 | McCambridge 2008 |  |  |  |  | Overall AUDIT score |  | "While the rates of refusal to provide a saliva sample are difficult to interpret, divergent self-reported outcomes among refusers suggest unreliability and are a cause for concern" | NR |
| Motivational interviewing 2011 | McCambridge 2011 |  |  |  |  | Overall AUDIT score |  | NR | NR |
| Drug education program 1980 (The Napa Project) | Moskowitz  1984 |  |  | Knowledge, benefits, attitudes, perceived peer use of hard and soft drugs |  |  |  | NR | NR |
| Project Northland | Komro 2008 |  | Alcohol purchase attempts | Norms supportive of use; Lack of resistance self-efficacy |  |  | The parent survey includes measures of neighbourhood and family constructs, including multi-item scales measuring: parental monitoring, family alcohol discussions, alcohol access in home, perceived community action, support of policies to prevent illegal sales, support of advertisement policies and perceived neighbourhood problems. Parental Involvement | NR | NR |
| RealTeen | Schwinn 2010 |  |  |  |  |  |  | NR | NR |
| Reconnecting youth | Hallfors 2006 | Academic performance GPA and truancy were obtained from school records; behaviour indicators including delinquent behaviors, prosocial weekend activities, and partying |  |  | emotional states including hopelessness, stress, anxiety, anger, self-esteem, depression, and perceived acceptability of suicide |  |  | NR | NR |
| Skills enhancement prog | Gilchrist 1987 | Interpersonal Behaviour. |  | Drug Knowledge and Attitudes. | Self-esteem | Self-identification as a user |  | NR | NR |
| SUCCESS | Clark 2010 |  |  |  |  |  | 30-day use of alcohol, marijuana, and illegal drugs excluding marijuana, and drinking to intoxication | NR | NR |
| Towards no drug abuse A | Sussman 1998 |  |  |  |  |  |  | NR | NR |
| Towards no drug abuse B | Sussman 2003 |  |  |  |  |  |  | NR | NR |
| Towards no drug abuse C | Valente 2007 |  |  |  |  |  |  | Peer use associated with increased substance use (total composite) in Intervention 2 | NR |
| Towards no drug abuse D | Sun 2008 |  |  |  |  |  |  | NR | NR |
| Towards no drug abuse E - universal | Dent 2001 |  |  |  |  |  |  | NR | NR |
| Family matters | Bauman 2002 |  |  |  |  |  | Smokeless tobacco, mediation analyses | NR | Costs of the resources used to implement the programme are provided and the cost per family has been calculated. Costs of programme development and evaluation excluded because they are not needed in replicating the programme. Office space and identifying eligible families costs are also not included. |
| LST - Botvin 6 | Botvin 1995 |  |  | Adjusted Follow-Up Behavioural Intention Means for Alcohol and Drug Use by Condition; Attitudes and normative beliefs, behavioural intentions, self-esteem, risk taking and self efficacy; Intention to use in next year, decision making and refusal skills |  |  |  | NR | NR |
| LST Botvin 10 | Botvin 1990 |  |  |  |  |  |  | NR | NR |
| LST Botvin 12 | Botvin 2015 |  |  |  |  |  |  | NR | NR |
| LST Botvin 29 | Botvin 2001 |  |  | Behavioural Intentions, Normative Expectations, Drug Attitudes and Knowledge, Social and Personal Competence |  |  | Implementation Fidelity | NR | NR |
| LST Botvin 56 | Botvin 1990a |  | Adolescent Risky Driving |  | HIV Risk Behaviour in Young Adulthood | High risk substance use subgroup analysis 13 years after baseline (1998) |  | NR | NR |
| LST - Seal 2006 | Seal 2006 |  |  | Life skills - refusal and decision making; Knowledge and attitudes |  |  |  | NR | NR |
| LST - Smith 2004 | Smith 2004 |  |  |  |  |  |  | NR | NR |
